# Genome-wide analysis of myxobacterial two-component systems: genome relatedness and evolutionary changes

**DOI:** 10.1186/s12864-015-2018-y

**Published:** 2015-10-13

**Authors:** David E. Whitworth

**Affiliations:** Institute of Biological Environmental and Rural Sciences, Aberystwyth University, Ceredigion, SY23 3DD UK

**Keywords:** Myxobacteria, *Myxococcus xanthus*, Histidine kinase, Response regulator, Mutation, Comparative genomics

## Abstract

**Background:**

Two-component systems (TCSs) are abundant prokaryotic signaling pathways, whose evolution is of particular importance because of their role in bacterial pathogenicity. Comparative genomics can provide important insights into the evolution of these genes, but inferences are dependent on the relatedness of the compared genomes. This study investigated the relationship between evolutionary distance and TCS evolution in myxobacterial genomes, of which there are several sequenced examples, of varying relatedness, and which encode large numbers of TCSs.

**Methods:**

Myxobacterial TCS gene sets were compared, orthologues defined, and changes in TCS properties such as gene organisation, domain architecture and size identified.

**Results:**

Genome relatedness/evolutionary distance was found to have a large effect on the apparent frequency of evolutionary events affecting TCS genes, but not on the relative dominance of different types of mutations. Large (≥1 gene) indels were the most common changes, often giving rise to gene organisation changes. Smaller indels were also common, sometimes changing domain architecture, and/or leading to pseudogene formation. Individuality of myxobacterial TCS gene sets seems primarily due to lineage specific gene loss. However, there is also evidence of extensive acquisition of genes by lateral transfer, with gene duplication also creating new TCS genes.

**Conclusions:**

This study provides catalogues of myxobacterial TCS gene sets and their orthology relationships, benchmarked against genome relatedness. It also provides insights into the relationship between evolutionary distance and the inference of TCS estudies of TCS evolution beyond the myxobacteriavolution, which may be important for studies of TCS evolutiThe online version of this articleon beyond the myxobacteria.

**Electronic supplementary material:**

The online version of this article (doi:10.1186/s12864-015-2018-y) contains supplementary material, which is available to authorized users.

## Background

Two-component systems (TCSs) are the dominant phosphorylation based signalling pathways in prokaryotes. The average eubacterial genome encodes ~50 TCS proteins [1, 2], and they regulate diverse and important behaviours, including pathComparative genomics and experimentalogenesis, cell cycle progression, sporulation and drug resistance [3]. Typical TCSs consist of two proteins – a histidine kinase (HK), which can sense an environmental parameter through an ‘input’ domain, and a response regulator (RR), which poComparative genomics and experimentalssesses an effector function (usually DNA-binding) via an ‘output’ domain. In response to an environmental signal the HK autophosphorylates within its ‘transmitter’ domain and the phosphoryl group is then transferred onto the ‘receiver’ domain of the partner RR, modulating its output domain effector activity. Common modifications to the basic scheme include hybrid kinases – proteins which contain both transmitter and receiver domains, and phosphotransfer proteins (PPs) - which can shuttle phosphoryl groups between receiver domains forming a phosphorelay. For comprehensive reviews of TCS biology, refer to the recent book by Gross and Beier [4] and the special issue of Current Opinion in Microbiology edited by Bourret and Silversmith [5].

Comparative genomics and experimental approaches have shed light on the general principles of TCS evolution [2, 6, 7]. For instance TCSs have evolved primarily by duplication and divergence of ancestral systems, with the contemporary diversity of TCS architecture generated by domain shuffling. This is important, as an understanding of natural variation due to evolution can inform the rational engineering of TCSs for biotechnological and research applications [8]. Nevertheless there are important questions left unanswered regarding TCS evolution and most studies have been forced to make several assumptions in their analyses. For instance, inferences regarding TCS evolution have been based on the comparison of various extant genomes. Common approaches are to use all available genomes, or to use multiple organisms from a single taxonomic unit. In both cases this typically results in comparisons between genomes which exhibit different relatedness to one another. Thus relatively distant and close genomes are considered equally, potentially distorting the conclusions drawn. This is particularly problematic for studies of TCS evolution, as it is thought that different evolutionary processes work at different timescales. For example, shuffling of HK input domains seems to occur rapidly post gene duplication, and gene gain/loss apparently dominates over gene fusion/fission at long timescales [9, 10]. In addition, studies are also often limited in power as there is sequencing bias within/between taxa, most organisms contain just dozens of TCS proteins, and at most taxonomic levels there is a relative shortfall in the number of available genomes, all of which can distort conclusions [1, 11–13].

Myxobacteria (order *Myxococcales*) are well known for their large numbers of TCSs, many of which have been characterised experimentally [14–16]. They exhibit complex developmental and predatory behaviours and are of ongoing importance to the biotechnology industry [17–20]. A major focus of myxobacterial research has been elucidation of the molecular mechanisms underpinning multicellular development, which are surprisingly plastic [21], and involve large numbers of TCS proteins [22].

At the time of analysis, genome sequences were available for 12 myxobacteria (Table 1). For ease of reference, genomes are abbreviated in the remaining text as shown in Table 1. Two of these genome sequences (*Pp* and *Ca*) are incomplete, but were included in the study anyway, as they were nearly complete, being composed of relatively small numbers of contigs (Table 1). *Bdellovibrio bacteriovorus* HD100 (*Bb*) was also included in the study as the bdellovibrionales are the closest known relatives of the myxobacteria.

**Table 1:** Order Myxococcales – genome properties, taxonomy and ecology

Organism	Size (Mbp)	Contigs	Proteins	%GC	Sub-order	Family	Fruits	Environment	Sequencing centre	Reference
*Myxococcus xanthus* DK1622 (*Mx*)	9.1	1	7316	68.9	Cystobacterineae	Myxococcaceae	Yes	Aerobic, terrestrial mesophile	The Institute for Genomic Research	[52]
*Myxococcus fulvus HW-1 (Mf)*	9.0	2^a^	7284	70.6	Cystobacterineae	Myxococcaceae	Yes	Aerobic, aquatic mesophile	Shandong University, China	[26]
*Corallococcus coralloides* DSM 2259 (*Cc*)	10.1	1	8033	69.9	Cystobacterineae	Myxococcaceae	Yes	Aerobic, terrestrial mesophile	Max Planck Institute, Marburg	[53]
*Stigmatella aurantiaca* DW4/3-1 (*Sa*)	10.3	1	8352	67.5	Cystobacterineae	Cystobacteraceae	Yes	Aerobic, terrestrial mesophile	Max Planck Institute, Marburg	[21]
*Anaeromyxobacter* sp. Fw109-5 (*AF*)	5.3	1	4466	73.5	Cystobacterineae	Myxococcaceae	No	Anaerobic, terrestrial mesophile	Department of Energy Joint Genome Institute	ncbi.nlm.nih.gov
*Anaeromyxobacter* sp. K (*AK*)	5.1	1	4457	74.8	Cystobacterineae	Myxococcaceae	No	Anaerobic, terrestrial mesophile	Department of Energy Joint Genome Institute	ncbi.nlm.nih.gov
*Anaeromyxobacter dehalogenans* 2CP-C (*AdC*)	5.0	1	4346	74.9	Cystobacterineae	Myxococcaceae	No	Facultative, terrestrial mesophile	Department of Energy Joint Genome Institute	[54]
*Anaeromyxobacter dehalogenans* 2CP-1 (*Ad1*)	5.0	1	4473	74.7	Cystobacterineae	Myxococcaceae	No	Facultative, terrestrial mesophile	Department of Energy Joint Genome Institute	[55]
*Sorangium cellulosum* So ce 56 (*Sc*)	13.0	1	9374	71.4	Sorangiineae	Polyangiaceae	Yes	Aerobic, terrestrial mesophile	Bielefeld University.	[56]
*Chondromyces apiculatus* DSM 436 (*Ca*)	9.4	128	7633	69.2	Sorangineae	Polyangiaceae	Yes	Aerobic, terrestrial mesophile	Institute of Microbial Technology	ncbi.nlm.nih.gov
*Haliangium ochraceum* DSM 14365 (*Ho*)	9.4	1	6719	69.5	Nannocystineae	Kofleriaceae	Yes	Aerobic, marine mesophile	Department of Energy Joint Genome Institute	[57]
*Plesiocystis pacifica* SIR-1 (*Pp*)	10.6	238	8450	70.7	Nannocystineae	Nannocystaceae	Yes	Aerobic, marine mesophile	J. Craig Venter Institute	[58]
*Bdellovibrio bacteriovorus* HD100 (*Bb*)	3.8	1	3586	50.6	Bdellovibrionaceae	Bdellovibrio	No	Aerobic, multiple habitat mesophile	Max Planck Institute, Tübingen	[59]

Myxobacterial genomes are generally large, with a characteristically high %GC. *Bdellovibrio bacteriovorus* HD100 is included as a representative of the bdellovibrios – the closest known relatives of the myxobacteria

^a^The *M. fulvus* genome includes a plasmid, which encodes no TCS proteins

This study sought to investigate how the apparent evolutionary mechanisms affecting myxobacterial TCSs change with timescale and/or phylogenetic distance. The analysis was restricted to myxobacterial genomes, as they possess large numbers of TCSs, and are available across a broad taxonomic range. Thus comparisons could be performed between organisms within the same species, genus, family, sub-order or order, with representative genomes available for all three sub-orders, and every family of the myxobacteria. Secondary aims of the study were to define myxobacterial TCS gene orthology relationships, and to identify core and accessory TCSs.

## Results

### Relatedness of sequenced genomes

**Fig. 1:**
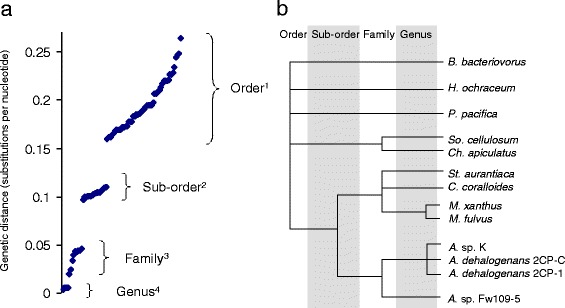
**a** Correlation between 16S rRNA gene sequence conservation and taxonomic classification. Pairwise genetic distances between Myxococcales 16S rRNA gene sequences (substitutions per nucleotide position), plotted in rank order. Distances cluster into four distinct groups: 1. *Ho/Pp* vs. *Sc/Ca* vs. *Mx/Mf/Cc/Sa/AK/AF/Ad1/AdC* and *Ho* vs. *Pp* (comparisons mainly between sub-orders), 2. *AK/AF/Ad1/AdC* vs. *Mx/Mf/Cc/Sa* (comparisons within a sub-order – across the extended Myxococcaceae family), 3. *Sc* vs. *Ca*, and *AK/Ad1/AdC* vs. *AF*, and *Mx/Mf* vs. *Cc* vs. *Sa* (comparisons within families), and 4. *AK* vs. *Ad1* vs. *AdC*, and *Mx* vs. *Mf* (comparisons within a genus). See text for abbreviations. **b** Phylogenetic tree with distances discretised into four vertical bands as grouped in part A

To obtain a measure of the divergence of the sequenced myxobacterial genomes, the 16S rRNA genes of each genome were aligned and distances (substitution frequencies per nucleotide) between each pair of sequences calculated, allowing construction of a phylogenetic tree (see Methods). There was strong bootstrap support for the tree (Additional file 1: Figure S1), which also agreed well with that of Garcia et al.*,* [23]. Distances clustered into four distinct groups, which largely shared particular taxonomic ranks (Fig. 1a). The ‘genus’ group is composed of distances between strains within the same genus (for example *Mx* vs. *Mf*, and *Ad1* vs. *AdC*). Comparisons between genomes in the same formally-defined family (see Table 1 for family and sub-order membership) were mainly distributed into two groups of genetic distances, here denoted ‘family’ and ‘sub-order’ in Fig. 1. The final ‘order’ group was dominated by distances between genomes from the same order, but different sub-orders (see the caption for Fig. 1a for details of group membership). A redrawn phylogenetic tree with distances discretised according to the groupings defined above is shown in Fig. 1b.

In several instances, distance-based groupings of genome pairs were not consistent with the formal taxonomy of the myxobacteria. For instance: 1, *Bb* is only as distant from the Myxococcales as *Pp* is from Myxococcales of different sub-orders, 2, *Ho* and *Pp* should be assigned to separate sub-orders, 3, *AF* should be in a separate genus to the other *Anaeromyxobacter*, 4, *Sa* belongs to the *Myxococcaceae* family (as opposed to the Cystobacteraceae family as stated by the taxonomy database of the NCBI), and 5, The *Anaeromyxobacter* should be a separate family from the other Myxococcaceae.

For the rest of this study we have classified inter-genome relationships according to Fig. 1 rather than using the current taxonomic classification. Thus for our purposes *AF* is considered to be in a different genus (but the same family) to *AK*, *Ad1* and *AdC*, while *Ho* and *Pp* are in separate sub-orders. *Ak*, *AF*, *Ad1* and *AdC* are considered to be in the same sub-order, but in a different family from *Mf*, *Mx*, *Cc* and *Sa*, a suggestion previously made by Garcia et al. [23].

### TCS protein sets

For each genome, a list of TCS proteins was inferred as described in the Methods section. Table 2 presents summary statistics for each TCS gene set, alongside myxobacterial mean values. Williams and Whitworth [24] provided a characterisation of the TCS genes from 1405 replicons as a pangenomic yardstick against which to compare TCS gene sets and these are included in Table 2 as bacterial mean values.

**Table 2:** Two-component gene sets of the myxobacteria

Genome^a^	TCS genes	Non-hybrid HKs	Hybrid HKs	PPs	RRs	% hybrid HKs	% TM HKs	% orphan	% paired	% complex	Intricate foci
*Mx*	282	103	37	5	137	26.4	47	35	35	30	8
*Mf*	288	98	49	3	138	33.3	41	34	33	33	9
*Cc*	306	106	56	2	142	34.6	38	34	35	31	8
*Sa*	338	124	70	4	142	36.1	40	34	34	32	9
*AdC*	188	68	17	1	102	20.2	48	43	35	22	2
*Ad1*	187	69	19	1	98	21.6	50	40	34	26	2
*AF*	207	72	35	0	100	33.3	50	28	33	39	5
*AK*	191	72	18	0	101	20.0	49	41	35	24	2
*Sc*	273	101	50	1	121	33.1	41	37	27	37	9
*Ca*	301	109	50	2	140	31.4	45	N/A	N/A	N/A	N/A
*Ho*	191	53	51	3	84	49.0	35	35	38	27	5
*Pp*	160	60	26	5	69	30.2	42	N/A	N/A	N/A	N/A
Myxobacterial mean:	242.7	86	39.8	2.3	114.5	30.8	44	36	34	30	5.9
*Bn*	92	32	16	2	42	33.3	73	37	48	18	4
Bacterial mean:^b^	31	14.8	3.3	0.6	15.6	18.2	63.5	46	40	14	0.3

For each genome, the numbers of TCS genes are presented, broken down by type. HKs are characterised according to the percentages that are hybrid proteins and that contain transmembrane helices. Genomic TCS gene organisation is also presented as the percentage of TCS genes which are orphaned, paired or in complex gene clusters. Values are presented for *Bdellovibrio bacteriovorus* (*Bb*), and the myxobacterial and bacterial means are also shown for comparison

^a^ *Ho* = *Haliangium ochraceum* DSM 14365, *Pp* = *Plesiocystis pacifica* SIR-1, *Sc* = *Sorangium cellulosum* So ce 56, *Ca* = *Chondromyces apiculatus* DSM 436, *Mx* = *Myxococcus xanthus* DK1622, *Mf* = *Myxococcus fulvus* HW-1, *Cc* = *Corallococcus coralloides* DSM 2259, *Sa* = *Stigmatella aurantiaca* DW3/4-1, *AK* = *Anaeromyxobacter* sp. K, *AF* = *Anaeromyxobacter* sp. Fw109-5, *Ad1* = *Anaeromyxobacter dehalogenans* 2CP-1, *AdC* = *Anaeromyxobacter dehalogenans* 2CP-C, and *Bb* = *Bdellovibrio bacteriovorus* HD100

^b^Calculated from Williams and Whitworth [24]

**Fig. 2:**
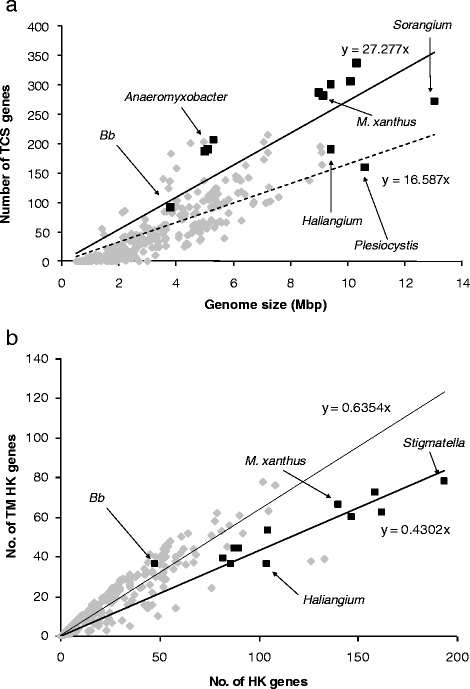
**a** Numbers of TCS genes as a function of genome size. Myxobacterial genomes (*black spots*) tend to have larger than expected numbers of TCS genes for genomes of their size. *Bdellovibrio bacteriovorus* HD100 (*Bb*) is also highlighted. A trendline is shown for all bacteria. **b** The proportion of transmembrane histidine kinases (TM HKs), as a function of total HK genes in myxobacterial (*black*) and bacterial (*grey*) genomes. *Bdellovibrio bacteriovorus* HD100 (*Bb*) is also highlighted. Trendlines and their equations are shown for bacteria (*dashed*) and myxobacteria (*solid*). Updated from [14]

Differences between the myxobacterial and bacterial means are apparent for some metrics. Myxobacteria are characterised by large numbers of TCS genes, greater than expected even for organisms with such large genomes. Figure 2a shows the number of TCS as a function of genome size for 316 bacteria and the 12 myxobacteria. Myxobacteria also tend to have a high proportion of hybrid HKs and a high proportion of TCS genes in complex foci (with a corresponding lack of TCS genes in orphan or paired foci). They also exhibit exceptionally low proportions of TM HKs (Table 2 and Fig. 2b), implying they sense their internal state to an unusual degree [25]. *Bb* exhibits values intermediate between the myxobacterial and bacterial means, except that (in contrast to the myxobacteria) it has a substantially greater proportion of paired TCS genes and TM HKs than the average bacterium (Table 2).

On inspection of Table 2, some myxobacteria stand out as unusual. The *Anaeromyxobacter* strains generally exhibit percentages of hybrid kinases more similar to bacteria than myxobacteria, and also possess relatively high percentages of TM HKs. *Sc*, *Ho* and *Pp* have relatively small numbers of TCS genes for myxobacterial genomes of their size. *Ho* and *Pp* are marine organisms, and marine organisms generally exhibit reduced environmental responsiveness compared to their terrestrial counterparts [25]. *Sc* could be thought of as having an exceptionally large genome for a myxobacterium, rather than having relatively low numbers of TCSs for a myxobacterial genome of its size.

### Orthology relationships between TCS proteins

TCS gene sets were clustered into groups of orthologous and paralagous proteins as described in the Methods section. Even at the lowest similarity cut-off used (30 % similarity), proteins clustered into pairs from *Mx* and *Mf* were also reciprocal highest-scoring BLAST hits, supporting the validity of this approach to define orthologues.

**Fig. 3:**
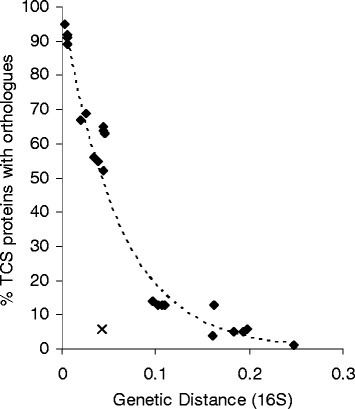
The percentage of orthologous TCS proteins varies exponentially with genetic distance. As genetic distance between two strains increases (substitution rate for the 16S rRNA gene), the percentage of proteins from either genome that are successfully clustered decreases exponentially. Each black diamond represents one pairwise comparison. Comparisons shown include all possible intra-genus and intra-family comparisons (ie. between *AF*, *Ad1*, *AdC* and *AK*, between *Mx*, *Mf*, *Sa* and *Cc*, and between *Sc* and *Ca*), and a representative sample of within sub-order and order comparisons (*Mf* vs. *Ho*/*AF*/*AK*, *Sc* vs. *Sa*/*Ho*/*Bb*, *Ho* vs. *Pp*, *Mx* vs. *Ad1*, *Sa* vs. *AdC* and *Ca* vs. *AF*). A notable departure from the trend is the comparison between *Ca* and *Sc* (*black cross*)

The percentage of proteins successfully grouped into clusters of orthologues decreased exponentially with increasing genetic distance (Fig. 3). A notable departure from the trend was the *Ca* and *Sc* comparison, which gave a remarkably low proportion of TCS genes being grouped as orthologues (6 %). This was the only pair-wise comparison which departed substantially from the trend, and the identified orthologues exhibited surprisingly low similarity. The cause of this extra variability is unclear.

#### Within-genus orthologues

**Fig. 4:**
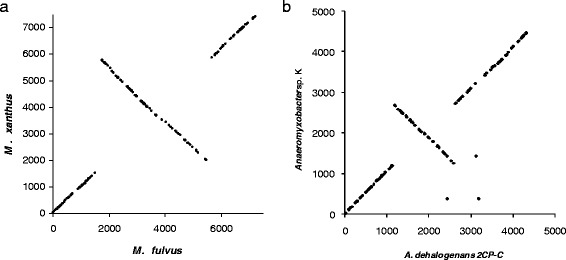
Dot-plots of TCS gene orthologues. Orthologues are plotted according to their genomic location (as inferred from the consecutive numbering of their locus tags) in both genomes. Each genome starts at the origin, with the *dnaA* gene being the first gene. A central line of orthologues extending from top left to bottom right indicates an inversion of a large section of the chromosome, centered around the origin/terminus. **a** Dot-plot of *Mx/Mf* TCS gene orthologues. **b** Dot-plot of *AdC*/*AK* orthologues

We began a detailed analysis of orthologues by comparing the gene set of *Mf* to that of *Mx*, the best characterised myxobacterium. *Mx* and *Mf* had 27 and 32 TCS genes respectively which were unique to that organism (singletons). The remaining TCS genes (89 %) all clustered into *Mx/Mf* pairs except for a single cluster of three proteins (LILAB_09435, LILAB_09385 and MXAN_7396), which seems to have arisen by a local duplication in the *Mf* lineage. A dot-plot of the relative location of TCS orthologues in the two genomes (Fig. 4a) recapitulated the global synteny observed between *Mx* and *Mf*, which is dominated by a single inversion [26]. There were no observable changes in relative genomic location of orthologues, suggesting that the relocation of individual TCS gene foci around a genome is a rare event, compared to re-location via large-scale chromosomal recombination. Five singleton TCS genes were found close to the putative inversion breakpoints (LILAB_00195, MXAN_1679/1680 and MXAN_5852/5853), indicating that gain/loss of those genes was potentially associated with the chromosomal inversion.

Within *Mx*/*Mf* paired clusters, TCS character (gene organisation, domain architecture, gene size and family membership) was conserved in the vast majority (247 of 254) of cases. In four pairs gene organisation was altered (MXAN_0259/LILAB_07355, MXAN_3738/LILAB_26865, MXAN_4580(Nla8)/LILAB_30920 and MXAN_5147(CheA3)/LILAB_33705), in two cases domain architecture/family was altered (MXAN_0727/LILAB_05150 and MXAN_6952/LILAB_11650A), and in one case both gene organisation and family membership changed between the two organisms (MXAN_4049/LILAB_28365A). Of the five observed changes in organisation, two were caused by gain/loss of intervening genes at a single focus, while the remaining three were caused by gain/loss of TCS genes. All three examples of family membership changes involved gain/loss of RR effector domains, and were associated with frameshift mutations. Intriguingly all three pairs included genes which were either mispredicted or annotated as pseudogenes, so it isn’t clear whether the observed differences represent true changes in family membership, or gene loss in progress.

There were seven cases of altered gene length (>100 aa difference) between *Mx* and *Mf* pairs, four of which involved gain/loss of effector/input domains. Two were changes of family membership mentioned previously (MXAN_4049/LILAB_28365A, MXAN_6952/LILAB_11650A), one was gain/loss of a duplicated PAS domain (MXAN_6586/LILAB_13600), and there was one instance of expansion/contraction of a poly-HAMP domain tract (MXAN_6735/LILAB_12790). The remaining three changes in gene length involved gain/loss of a C-terminal region (MXAN_4461(RomR)/LILAB_30440), extension/contraction of an N-terminal region (MXAN_0230/LILAB_07550), and gain/loss of an internal region (MXAN_4758/LILAB_31740), all of which may represent the gain/loss of currently uncharacterised domains.

Across the whole *Mx/Mf* genome, 23 % of protein-encoding genes were unique to either genome, whereas only 10–11 % of TCS genes were singletons, implying that TCS genes are a relatively conserved gene set. *Mx* TCS genes with names have published phenotypes, and historically *Mx* has been subjected to exhaustive genetic screens for developmental and motility mutations. The set of named TCS genes is therefore enriched for genes with significant phenotypes (particularly motility and/or development-related). *Mx*/*Mf* paired TCS genes were enriched for named genes –24 % of all *M. xanthus* TCS genes are named, however only two of the 27 singletons were named (7 %), and those two genes (*nla1* and *nla13*) have minor phenotypes [27], suggesting that singletons are not subject to a strong retentive selective pressure. The *Mx*/*Mf* singletons were very different in character - *Mx* encoded eight hybrid kinases, 11 TCS genes with complex gene organisations and 11 paired TCS genes, whereas the *Mf* singletons included no hybrid kinases, only three complex TCS genes, but 17 paired genes.

To see whether *Mx*/*Mf* differences were typical of species-level comparisons, Anaeromyxobacter strains *AK* and *AdC*, were also compared. The *AdC*/*AK* genetic distance (16S) is identical to that between *Mx* and *Mf*, however the Anaeromyxobacter species are anaerobic, and non-fruiting, so any commonality between the *AK*/*AdC* and *Mx*/*Mf* comparisons is likely to be due to genetic similarity and independent of lifestyle. No duplications were observed, while 9 % of genes were singletons (16 and 19 in the two genomes). Six changes in gene organisation were observed (AnaeK_0372/Adeh_3183, AnaeK_0373/Adeh_2444, AnaeK_1885/Adeh_1993, AnaeK_1886/Adeh_1992, AnaeK_1889/Adeh_1989, AnaeK_4383/Adeh_4250), one change in size and domain architecture (AnaeK_2395/Adeh_1473) and one other gene size change (AnaeK_0940/Adeh_0892). A dot-plot of *AK*/*AdC* orthologues (Fig. 4b) shows global synteny, including across a large chromosomal inversion, albeit with three pairs of orthologues demonstrating re-location of TCS genes within the chromosome (AnaeK_1417/Adeh_3134 and AnaeK_0372/Adeh_3183), including one causing a change in gene organisation (AnaeK_0373/Adeh_2444).

Insertions/deletions (indels) of ≥1 gene at three foci (Adeh_2443, AnaeK_4382 and AnaeK_1888), resulted in four singletons, and the remaining five changes in gene organisation. One small (<1 gene) indel (AnaeK_2395, AnaeK_2396/Adeh_1473) resulted in one singleton, and a domain architecture change and gene size change, as the consequences of changing between a TR,T system and a TT system (domain organisation of a locus is represented using R, T and H to represent receiver, transmitter and HisKA/Hpt domains respectively, with commas separating genes, as explained in the Methods section). Finally, an indel resulted in an apparent gain/loss of two input domains from one HK (AnaeK_0940/Adeh_0892). Unlike with the *Mx*/*Mf* singletons, there were no obvious differences in character between the *AdC* and *AK* singletons.

In summary (Table 3), these two within-genus comparisons (in agreement with the other two comparisons *Ad1* vs. *AK*/*AdC* – data not shown), suggest that inter-species differences are dominated by TCS gene gain/loss, occasionally causing changes in gene organisation. This also leads to ‘individuality’ within each genome, with organism-specific ‘accessory’ genes. Relatively infrequently, changes in domain architecture occur as a consequence of small indels or frameshift mutations. As expected, such alterations most frequently affect the output domains of RRs (changing family membership) and the sensory domains of HKs.

**Table 3:** Mutational events and their consequences for TCS gene sets

Apparent mutational event	Within-genus frequency (%)	Within-family frequency (%)	Typical consequences
Chromosomal Rearrangements	~1-2 %	~3 %	Gene gain/loss.
Large (≥1 gene) indels	~10 %	~20	Gene gain/loss, with occasional changes in gene organisation (in ~10 % of cases).
- Duplications	<<1 %	~3 %	Gene gain.
- Horizontal transfer	~2 %	~4 %	Gene gain.
Small (<1 gene) indels	~1-2 %	~3 %	Domain gain/loss, changes in family.
- Frameshifts	<1 %	~1 %	Change in family (often through changes in RR output domains), pseudogene formation.

Percentages of orthologous clusters which show evidence of evolutionary changes when comparing genomes from the same genus (within-genus) and the same family (within-family). Percentages obtained from pairwise comparisons between *Cystobacterineae* members

#### Within-family orthologues

To assess TCS gene changes between genera, three ‘within-family’ groups of comparisons were made; *Cc*/*Sa*/*Mx*/*Mf*, *Ad1*/*AdC*/*AK*/*AF*, and *Sc*/*Ca*.

In *Cc*/*Sa*/*Mx*/*Mf* comparisons, some clusters of homologues were observed with >4 members. One cluster of six proteins was observed due to an apparent double-duplication in *Sa* (STAUR_0029, STAUR_4565, STAUR_7210), while single duplications in *Cc* and *Mf* gave rise to two clusters of five proteins (COCOR_07667/COCOR_07814 and LILAB_09385/LILAB_09435 respectively). A final cluster of five proteins was composed entirely of *Sa* proteins (STAUR_1021, STAUR_1353, STAUR_5099, STAUR_7439 and STAUR_7639). 572 proteins were found in clusters of four (143), all but two of which included a single representative from each genome (one cluster contained a *Mf* duplication LILAB_11650A/LILAB_19280 while the second contained duplicated proteins from *Cc* and *Sa* COCOR_01454/COCOR_02086/STAUR_6958/STAUR_8144). Another 204 proteins were found in groups of three, with members from different genomes, however in two cases there was an apparent duplication in one of the genomes (COCOR_1905/COCOR_04361 and COCOR_07670/COCOR_07671). 206 proteins were paired, including five pairs of duplicated proteins from *Sa* (STAUR_0493/STAUR_2886, STAUR_1273/STAUR_4462, STAUR_1775/STAUR_5256, STAUR_1837/STAUR_4550 and STAUR_4571/STAUR_4572). This left 211 singletons, of which the majority belonged to *Sa* (124, 59 %), with large numbers also in *Cc* (64, 30 %), and relatively few in *Mx* (13) or *Mf* (10). The pattern of protein orthology can be seen in Additional file 2: Figure S2 as a Venn diagram, and numbers are consistent with genetic distance, with closer genomes sharing more orthologues and possessing fewer singletons. The same trends were observed for *Ad1*/*AdC*/*AK*/*AF* orthologues (Additional file 2: Figure S2).

Across the 318 *Cc*/*Sa*/*Mx*/*Mf* clusters with two or more members, in addition to the *Mx*/*Mf* changes described earlier, there were 43 cases of altered gene organisation (highlighted in Additional file 3), and three changes of domain architecture, all three of which involved hybrid kinases. A small indel caused a RRT/RT change (MXAN_6966 is the *Mx* orthologue), a TRT/TRTTRT change appears associated with a tandem duplication and fusion (STAUR_6958), while a TR/T change is associated with a large indel, also involving gain/loss of an adjacent gene (STAUR_7785). In addition to these changes in domain architecture, 21 cases of genes changing size by >100 aa were observed, of which the majority (16) affected HKs, mainly in their N-terminal regions (highlighted in Additional file 3).

For *Ad1*/*AdC*/*AK*/*AF* orthologues (196 clusters of two or more proteins), 17 instances of changes in gene organization were observed (highlighted in Additional file 3). These were mainly (15 cases) associated with large (>1 gene) indels, with five cases also involving gene rearrangements (adjacent to *Ad1* genes A2cp1_1514, A2cp1_2989, A2cp1_2499 and A2cp1_3138 and A2cp1_3377), and two of the 17 were due to frameshift mutations generating pseudogenes (the *AdC* orthologue of A2cp1_1973, and the *Ad1* homologue of Adeh_1992). Four changes in domain architecture were observed, each of which was associated with a change in gene size. In one case an N-terminal PilZ domain was gained/lost from a RR (Anae109_3862), while the other changes were due to gene fusion/fission events (T,TR/TT and T,R/TR changes involving Adeh_1473 and A2cp1_0976 respectively). Seven gene duplications at five loci were apparent (Anae109_0467/Anae109_3535, Anae109_0469/Anae109_3533, Anae109_0470/Anae109_3532, Anae109_1027/Anae109_2976, Anae109_1440/Anae109_3416, Anae109_1441/Anae109_3417, A2cp1_3106/A2cp1_3138), with an additional duplication also associated with a change in gene size (Anae109_1040/Anae109_2356). Finally, two additional changes in gene size were seen, both of which were due to gain/loss of C-terminal regions (one HK and one RR; Adeh_3180 and Anae109_0769).

The final within-family comparison possible is between *Ca* and *Sc*, however that comparison is characterised by an unusually low proportion of orthologous proteins (Fig. 3) and low similarity between orthologues. Of the 18 clusters of orthologues which included members from both organisms, there were no apparent changes in domain architecture, gene organisation or gene size. Further comparisons between more distant genomes were also uninformative due to the small number of orthologous protein clusters identified by CD-HIT or by bidirectional best-scoring BLAST hits. Thus, in summary, comparisons between same-family members show similar trends to those from same-genus comparisons albeit at higher frequencies (Table 3).

### Complexity

Another notable feature of myxobacterial TCS proteins is their complexity – both in terms of their domain architectures, and their gene organisation [14, 15].

We define here the most complex systems as ‘intricate’, where at least two receiver and at least two transmitter domains are found in the same protein and/or genetic focus. TRTR proteins are encoded as singletons in four genomes (*Cc, Ho, Sa* and *Sc;* COCOR_05524, Hoch_3141, STAUR_7959 and sce3507), while *Sa* also encodes a TRTTRT protein (STAUR_6958). According to our analysis, the TRTR proteins are not orthologous to each other or other myxobacterial TCS proteins, while the TRTTRT protein appears to be a tandem duplication and fusion of two adjacent TRT proteins. In addition, myxobacterial genomes possessed between two and nine intricate multi-gene TCS foci (Table 2), considerably more than the bacterial mean.

The intricate foci of *Ho*, *Sc* and *AF* were largely unique to those genomes, otherwise orthologous relationships between the intricate foci were relatively clear at the genus and family level (Table S1). The foci highlighted exhibit several interesting properties. There seems to be a great deal of plasticity in these foci with many exhibiting orthology with non-intricate TCS foci as a consequence of gene gain/loss and/or recombination (Additional file 4: Table S1). In addition, a large proportion of the TCS domains encoded at intricate foci are found in hybrid kinases (111 of 145 domains, 77 %).

The proportion of foci containing large numbers of TCS domains are similar between bacteria and myxobacteria (data not shown). Nevertheless, when considering all TCS proteins (not just intricate foci/proteins), those of myxobacteria are generally more complex than other bacteria. Table 4 shows the relative frequencies of TCS proteins with different domain compositions for myxobacteria and bacteria. Myxobacteria possess far greater proportions of TCS proteins with two or more transmitter domains, and hybrid kinases with three or more receiver domains than other bacteria.

**Table 4:** Transmitter and receiver domain composition of TCS proteins

% myxobacteria / % bacteria	0 R	1 R	2 R	3 R	4+ R
0 T	0.41 (28)	0.92 (1340)	1.58 (32)	2.13 (2)	^a^(0)
1 T	0.97 (1026)	1.42 (373)	1.37 (54)	3.73 (22)	14.91 (4)
2+ T	7.45 (1)	11.75 (26)	5.73 (5)	0† (0)	0^b^ (0)

Values are the % of myxobacterial TCS proteins exhibiting the number of receiver (R) and transmitter (T) domains in question divided by the % of all such bacterial TCS proteins [24]

^a^There are no such proteins in bacteria

^b^No such proteins in myxobacteria (only 0.009 % of all TCS proteins in bacteria have 2+ transmitter and 3+ receiver domains). Numbers of such myxobacterial proteins are shown in parentheses

### TCS gene expansion

Nearly two thirds (59 %) of all *Mx*/*Mf* singletons were found to have orthologues in *Cc* and/or *Sa*, implying that the formation of the majority of singletons was through progressive gene loss. However, genes can also be gained by duplication and divergence. Yet duplication is responsible for a relatively small amount of differences between TCS gene sets. For instance, clustering reveals only 12 lineage-specific duplications in *Sa*, four cases in *Cc* and two in *Mf*. Eight duplications were apparent within the *Anaeromyxobacter* strains (highlighted in Additional file 3).

Nevertheless, it appears that certain TCS families have been expanded hugely in the myxobacteria and this occurred relatively recently. For instance, NtrC family members are unusually numerous [14]. Indeed, eight of the 28 *Mx* NtrC family members exhibited more similarity to other *Mx* NtrC paralogues than to homologues in other organisms, suggesting origin by duplication after the divergence of contemporary genera of myxobacteria.

Another mechanism that can increase gene number is horizontal gene transfer (HGT). To test the frequency of horizontal acquisition, the *Mx*/*Mf* singletons lacking homologues in *Cc*/*Sa*/*Mx*/*Mf* were queried against the nr protein database using BLAST. Eight of the ten *Mf* singletons and six of the 13 *Mx* singletons gave BLAST hits across the *Myxococcales*, implying linear descent. However nine of the 23 *Mx*/*Mf* genes gave best non-self BLAST hits to non-*Myxococcales* genomes, implying horizontal acquisition. Such evidence of HGT is also abundant in the singletons of other myxobacteria (eg. for 34 of the 78 singletons of *Ad1*/*AdC*/*AK*/*AF* (Additional file 5: Table S2)).

## Methods

### Phylogenetic analysis

16S rRNA gene sequences were aligned using ClustalW [28], using default parameter settings, and neighbour-joining trees were created using PHYLIP [29] with 1000 bootstraps, excluding columns containing gaps.

### Inference of TCS proteins sets

Eight myxobacterial genomes (*Mx, Mf, Sc, Ho, AF, AK, Ad1* and *AdC*) and that of *Bb* are included within the latest version of the P2CS (Prokaryotic 2-Component Systems) database, available at www.p2cs.org [30, 31], which provides an automated and consistent pipeline for the identification and annotation of TCS genes. The same categorisation pipeline is available for unpublished sequences at www.p2rp.org [32] and this service was used to characterise the TCS gene sets for *Sa*, *Cc, Pp* and *Ca* nucleotide sequence files from www.ncbi.nlm.nih.gov. TCS gene sets were compared with those previously published for *Mx, Sa, AdC* and *Sc* [14], and discrepancies manually curated. For the TCS gene sets available through P2CS, potentially ‘mispredicted’ TCSs were identified, and these were validated manually and added to the TCS datasets (named after the closest gene, and given an ‘A’ suffix). These two processes resulted in the addition of five, six and 14 TCS proteins to the gene sets for *Mx, Sc* and *AdC* respectively, as originally described by Whitworth and Cock [14]. Three, one, one and three ‘mispredicted’ TCS genes were also validated for *Mf, Ho, Ad1* and *AF* respectively. TCS datasets for each genome are available as additional data (Additional file 6).

### Characterisation of TCS proteins

Domain architectures and the presence of transmembrane (TM) helices within TCS proteins were determined as described by Barakat et al. [33]. TCS gene organisation was defined using a classification scheme based on TCS domains (receiver, transmitter, Hpt/HisKA) encoded unidirectionally at a ‘focus’, as proposed by Williams and Whitworth [24]. If a transmitter and a receiver domain were found adjacent in the genome (whether within a single ‘hybrid kinase’ gene, or as separate HK and RR genes with no more than one intervening gene), and they had at least two non-TCS genes on each side, then the relevant genes were described as ‘paired’ (i.e. that focus appears to encode an entire TCS). If a gene contained a single transmitter, receiver or phosphotransfer domain and was found more than two genes from another TCS gene, it was described as ‘orphan’. TCS gene foci with any other domain/gene organisation were described as ‘complex’. The genomes of *Pp* and *Ca* are incomplete, so gene organisations were not defined for those organisms. Focus TCS domain organisation is described in the text using R, T and H to represent receiver, transmitter and HisKA/Hpt domains respectively, with commas separating genes. Thus a ‘RT,R’ organisation represents a hybrid kinase gene encoding an N-terminal receiver domain and a C-terminal transmitter domain, adjacent to a response regulator gene encoding a single receiver domain.

### Sequence-based clustering

To define orthologues and paralogues, sets of TCS genes were clustered using CD-HIT [34]. A high similarity cut-off was used (up to 90 % similarity) to give high confidence clusters, and then a lower similarity (30 %) cut-off was used to identify more divergent clusters. Clusters obtained were then subjected to curation, whereby any large clusters containing multiple paralogues obtained using the 30 % cut-off were manually split into the high-confidence clusters obtained with a 90 % cut-off. Clusters which included proteins of significantly different length (>100 amino acid residues difference), or of varying domain architecture, family membership and/or gene organisation, or with varying numbers of members per genome were earmarked for further analysis.

## Discussion

### Genetic distance and inferred evolutionary events

Comparing the TCSs from organisms with differing degrees of relatedness allowed inference of the contribution of different types of mutation to the evolution of contemporary sets of TCSs. It appears that the relative impact of different mutational events are not affected by the relatedness of the organisms being compared, however, the apparent frequency of mutations is dependent on the relatedness of compared genomes (Table 3), echoing the findings of He et al. [35] in their study of clostridia. This means that when comparing the gene sets of multiple genomes, it is important to characterize the similarity of those genomes and compensate for differing degree of relatedness. It is even worth including organisms of diverse relatedness, to increase the power of analysis, as suggested by Eisen et al.*,* [36]. In this study we used 16S rRNA gene sequence similarity as a straightforward indicator of genomic relatedness/distance and found that the ability to identify TCS orthologues between genomes was dependent on relatedness (Fig. 3). At low levels of relatedness, difficulties in the identification of orthologues reduced the power of comparative analysis making it difficult to infer evolutionary events. This difficulty was likely exacerbated by the large numbers of TCS in myxobacterial genomes, and should be less of a problem for the majority of prokaryotes.

There have been several studies comparing the TCS gene sets of related organisms, and although not noted, the phenomena we describe can often be seen in the TCS gene sets of diverse taxa. For instance, TCS gene complements can vary by 10–30 % when comparing organisms within the same family/genus, and the frequency of within-species variation is about half that observed when comparing organisms within the same genus [37–40].

### Evolution of myxobacterial TCS gene sets

Our data suggest that most changes in myxobacterial TCS gene sets are due to large indels, some of which are associated with chromosomal recombination. There is evidence of frequent gene loss (which has also dominated TCS evolution in the *Lactobacillaceae* and *Leuconostocaceae* [39]), but also gene gain via HGT and occasional gene duplications. Some (~10 %) of these indels result in changes in gene organization, when the indel only affects part of a TCS locus. Small (within-gene) indels are less frequent and often result in domain gain/loss with associated changes in TCS family membership. Other studies have reported broadly consistent results. For example, in a study of recombination rates in *Salmonella*, Sun et al.*,* [41] found that deletions are significantly more frequent than duplications. Both *Mx* and *Bb* exhibit genome-wide evidence of HGT [42, 43], and we find that HK and RR genes tend to be acquired/lost as pairs, which has been observed in several other taxa, including mutans streptococci and *Xanthomonas* spp. [40, 44].

Domain shuffling appears to have happened occasionally during TCS evolution, giving rise to HKs with a multiplicity of input domains, and RRs with diverse output domains [45]. Our methods of defining orthologues used whole gene sequences and may not have been capable of identifying shuffled domains, nevertheless we did observe several instances of input/output domain gain/loss. With domain shuffling being a rare evolutionary event, it seems probable that apparent domain shuffling is a consequence of domain loss followed by domain gain rather than direct shuffling between proteins.

We also did not observe significant relocation of TCS within genomes. The few cases observed (for example see Fig. 4b) were associated with breakpoints of chromosomal recombination. It is possible that those chromosomal relocations occurred due to recombination between homologous TCS genes at those points, however there is too little conservation of synteny in the break point regions to provide any evidence of this happening in myxobacterial genomes.

Myxobacteria were chosen as the subject of this study due, in part, to the large number of TCS genes they possess. Potentially, this could limit extrapolation of our conclusions to other organisms, as increased numbers of TCS genes may have promoted some mutagenic events (for example homologous recombination or gene conversion), and the large numbers of paralogous genes makes robust orthologue identification difficult.

In 2008 we described the TCS of four sequenced myxobacteria and used domain-based orthology searches to identify likely evolutionary events and peculiarities of myxobacteial TCS gene sets [14]. For this study we added a further eight myxobacterial genomes, dramatically increasing the number of inter-genome comparisons possible, and defined orthologues using whole gene sequence-based methods. The results obtained here agree well with that previous work, and provide a less anecdotal analysis of evolutionary events. Benchmarking our genome comparisons by relatedness also allows straightforward comparison with TCS gene sets from other genomes in the future.

The TCS genes of myxobacteria seem to be relatively conserved compared to non-TCS genes, and we also found that the *Mx* TCS genes with *Mf* orthologues were enriched for named genes, which tend to have observable phenotypes. This suggests that there is a ‘core’ set of TCS genes in myxobacteria responsible for regulating important functions, with other ‘accessory’ TCS genes being more variable between genomes, and with minor (if any) observable phenotypes.

The *Ad1*/*AdC*/*AF*/*AK* TCS gene sets exhibited much less variability than those of *Mx*/*Mf*/*Cc*/*Sa* (see text and Additional file 2: Figure S2), however this effect was proportional with the reduced numbers of TCS genes in the anaeromyxobacteria. It is possible that the fruiting myxobacteria need enhanced ‘individuality’ to allow easy discrimination of competing genotypes, but whether this is a selected trait, or purely a consequence of their increased numbers of TCS is uncertain. Among strain-specific genes, there is particularly good evidence of acquisition by HGT, which is known to have had a major effect on the shaping of contemporary myxobacterial genomes, alongside lineage-specific expansion [43].

### Myxobacterial genomics

The field of myxobacterial genomics is currently hampered by the large number of genes encoded in each genome (and the consequent number of differences between genomes), the lack of easily assayable behavioural outputs (for instance fruiting body formation is typically just characterized by time, and % of wild-type spores produced), and pleiotropy of many genes. This means that phenotypic properties cannot easily be correlated with particular genomic differences.

However, further myxobacterial genomes are becoming available at an accelerating rate, and it is possible that in the near future, physiological characterisation of the new genome host organisms will enable correlations with genomic features. To that end, it is important to develop benchmarked catalogs of genomic differences. Taking into consideration the relative distance of compared genomes is an important feature of benchmarking, and this study provides a framework to develop such benchmarks using 16S ribosomal RNA gene sequence differences as indicators of genetic distance, which can be related to formal taxonomy and other systematic methods [46, 47].

During the course of this study draft genomes became available for strains of *Cystobacter fuscus*, *Cystobacter violaceus*, *Enhygromyxa salina*, *Hyalangium minutum*, *Sandaracinus amylolyticus*, and for two further strains of *Myxococcus xanthus*, including DZ2 and DZF1 [48, 49], however these genomes are not complete or scheduled for finishing. Thankfully, two complete myxobacterial genomes have also became available, *Myxococcus stipitatus* DSM 14675 and *Sorangium cellulosum* So0157-2 [50, 51]. Both of these genomes are now characterized in P2CS and seem typical members of their genera, albeit with slightly more TCS genes (309 in *Sc* So0157-2, 296 in *M. stipitatus* [*Ms*]), except that the LytTR family of RRs is expanded substantially in *Ms* (12 compared to two in each of *Mx* and *Mf*). As more genomes are completed it will become easier to identify more distant orthologues via ‘gap-bridging’ genomes. This will in turn improve deduction of the directionality of evolutionary changes such as indels, and allow us to create a more finely resolved timeline of myxobacterial TCS gene evolution.

## Conclusion

Focusing on the TCS genes of myxobacteria, this study investigated the relationship between apparent evolutionary events, and genomic distance or ‘relatedness’. As genomic distance increases it becomes more difficult to identify TCS orthologues, and the relative frequency of evolutionary events increases. However, the relative frequency of evolutionary changes remain unaffected by scale (or lineage), and it seems that in the myxobacteria, as for other organisms, TCS gene evolution is dominated by large indels (often causing changes in gene organization), with smaller indels being less frequent (occasionally causing changes in domain architecture). TCS genes are ‘born’ by duplication and acquisition through HGT, but the individuality of TCS gene sets appears to be dominated by lineage-specific gene loss.

More generally, our results suggest that researchers should be mindful that the numbers of genomes compared can have a substantial effect on the apparent frequency of observed events. This is particularly true when comparing organisms that share different levels of ‘genetic relatedness’, which should not be assumed to always be consistent with the taxonomic assignments of compared organisms.

## Additional files

Additional file 1: Figure S1. Genetic distance (substitutions per nucleotide position) and neighbour-joining tree inferred from myxobacterial 16S rRNA gene sequences. Bootstrap support for each clade was > 95 % (from 1000 bootstrap resamplings) in all cases except for the clade indicated by a filled circle (89 % bootstrap support). The tree is rooted with *B. bacteriovorus*. The bar represents 0.1 substitutions per nucleotide position. (PPT 119 kb) Additional file 2: Figure S2. Orthology relationships within-family. Venn diagrams showing the distribution of orthologous genes between organisms. Some orthologous clusters exhibited multiple members from the same organism, suggestive of lineage-specific gene duplications. For the purpose of these diagrams, duplicated proteins were treated as singletons. For instance, if a cluster contained members from *Sa*, *Cc*, *Mx* and *Mf*, but the *Sa* protein was duplicated, then 1 was entered in the *Sa*/*Cc*/*Mx*/*Mf* cell, and 1 into the *Sa* only cell. For a pair of *Sa* proteins, 2 was entered into the *Sa* cell. a: *Sa*/*Cc*/*Mx*/*Mf*, b: *AF*/*AK*/*Ad1*/*AdC*. (PDF 185 kb) Additional file 3:
**The sequence clusters of orthologues of every myxobacterial TCS protein.** (DOC 279 kb) Additional file 4: Table S1. Intricate multi-gene TCS foci. Each row shows all myxobacterial orthologues of intricate multi-gene foci, including non-intricate orthologues, arranged by organism. Foci in particular genomes are denoted by the number from the locus tag of the first protein within the focus. ‘//’ refers to an unspecified number (2 or more) of non-TCS genes. (PDF 39 kb) Additional file 5: Table S2. Evidence of horizontal gene transfer (HGT) amongst cystobacterial TCS proteins. Singletons (TCS lacking orthologues) from comparisons between *Ad1*/*AdC*/*AK*/*AF*, and between *Mx*/*Mf*/*Cc*/*Sa*, are presented alongside the number of singletons acquired by HGT (as evidenced by highest-scoring BLAST hits to non-myxobacteria outside the organism’s own genus). Also tabulated are the number of TCS genes found in foci where every TCS gene was acquired by putative HGT and the number of those which constitute an ‘intact’ TCS (ie containing ≥1 transmitter domain and ≥1 receiver domain. (PDF 456 kb) Additional file 6:
**Datasets of the annotated TCS proteins from each genome.** (XLS 586 kb)

## Abbreviations

TCS: Two-component system
HK: Histidine kinase
RR: Response regulator
TM: Transmembrane
