# Supplementary material for: Genome-wide analysis of myxobacterial two-component systems: genome relatedness and evolutionary changes
Source: BMC Genomics. 2015 Oct 13;16:780. doi: 10.1186/s12864-015-2018-y (PMC4603909; doi:10.1186/s12864-015-2018-y)
Supplement: Additional file 1: Figure S1. — Genetic distance (substitutions per nucleotide position) and neighbour-joining tree inferred from myxobacterial 16S rRNA gene sequences. Bootstrap support for each clade was > 95 % (from 1000 bootstrap resamplings) in all cases except for the clade indicated by a filled circle (89 % bootstrap support). The tree is rooted with B. bacteriovorus. The bar represents 0.1 substitutions per nucleotide position. (PPT 119 kb) [file 12864_2015_2018_MOESM1_ESM.ppt]

## Slide 1
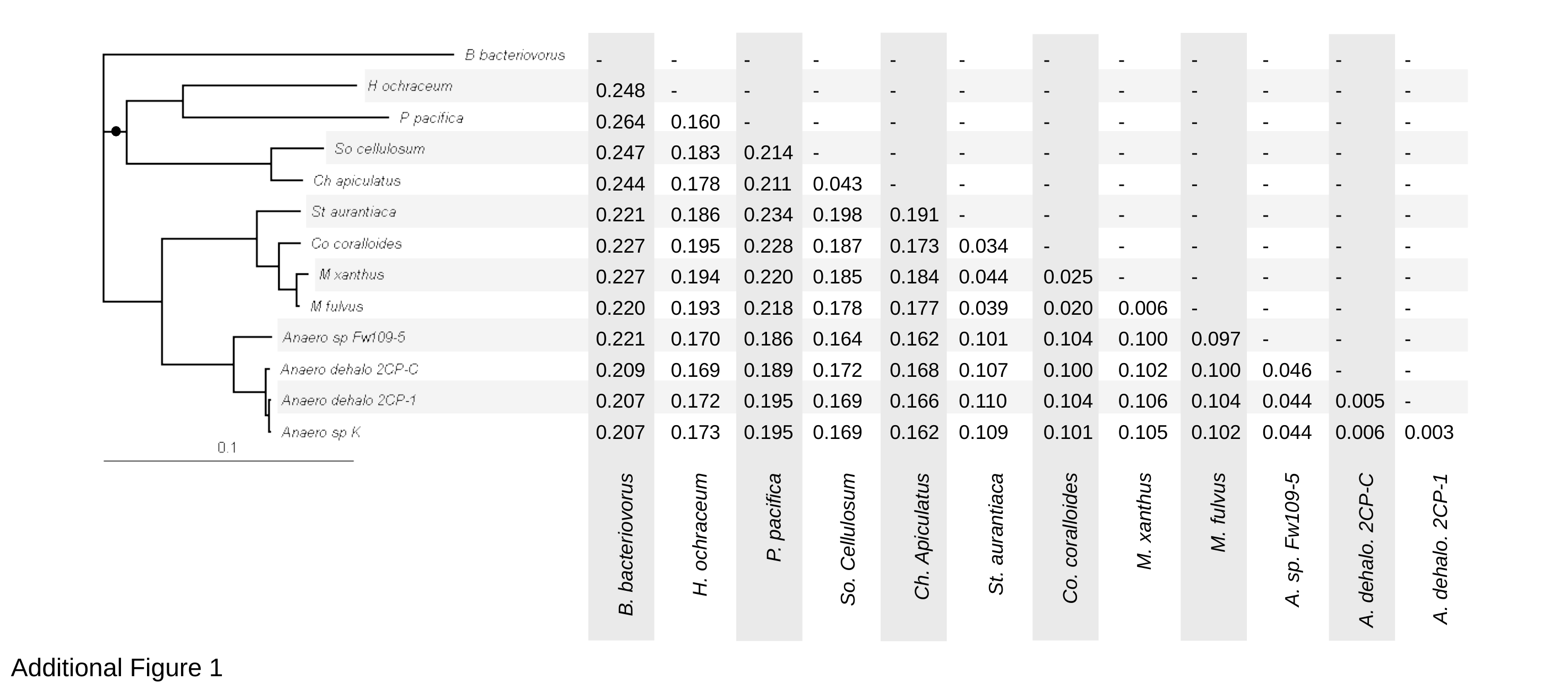

-
0.248
0.264
0.247
0.244
0.221
0.227
0.227
0.220
0.221
0.209
0.207
0.207
-
-
0.160
0.183
0.178
0.186
0.195
0.194
0.193
0.170
0.169
0.172
0.173
-
-
-
0.214
0.211
0.234
0.228
0.220
0.218
0.186
0.189
0.195
0.195
-
-
-
-
0.043
0.198
0.187
0.185
0.178
0.164
0.172
0.169
0.169
-
-
-
-
-
0.191
0.173
0.184
0.177
0.162
0.168
0.166
0.162
-
-
-
-
-
-
0.034
0.044
0.039
0.101
0.107
0.110
0.109
-
-
-
-
-
-
-
0.025
0.020
0.104
0.100
0.104
0.101
-
-
-
-
-
-
-
-
0.006
0.100
0.102
0.106
0.105
-
-
-
-
-
-
-
-
-
0.097
0.100
0.104
0.102
-
-
-
-
-
-
-
-
-
-
0.046
0.044
0.044
-
-
-
-
-
-
-
-
-
-
-
0.005
0.006
-
-
-
-
-
-
-
-
-
-
-
-
0.003
B. bacteriovorus
H. ochraceum
P. pacifica
So. Cellulosum
Ch. Apiculatus
St. aurantiaca
Co. coralloides
M. xanthus
M. fulvus
A. sp. Fw109-5
A. dehalo. 2CP-C
A. dehalo. 2CP-1
Additional Figure 1
