# Supplementary figures and images for: Genome-wide analysis of myxobacterial two-component systems: genome relatedness and evolutionary changes
Source: BMC Genomics. 2015 Oct 13;16:780. doi: 10.1186/s12864-015-2018-y (PMC4603909; doi:10.1186/s12864-015-2018-y)

a:

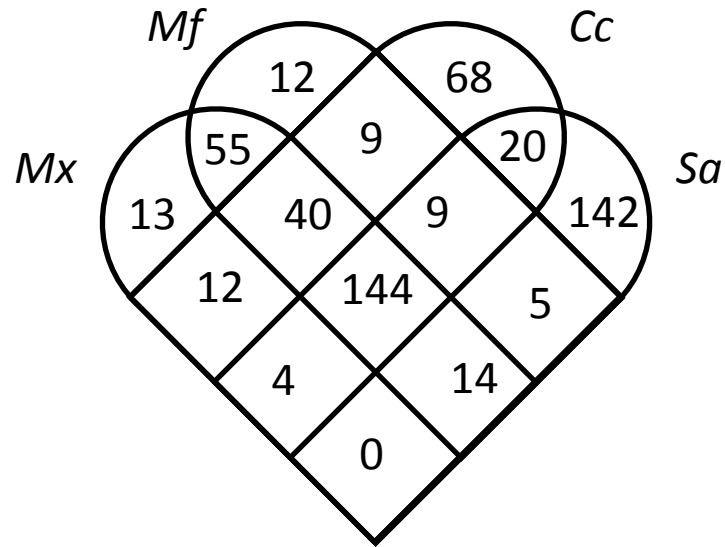

b:

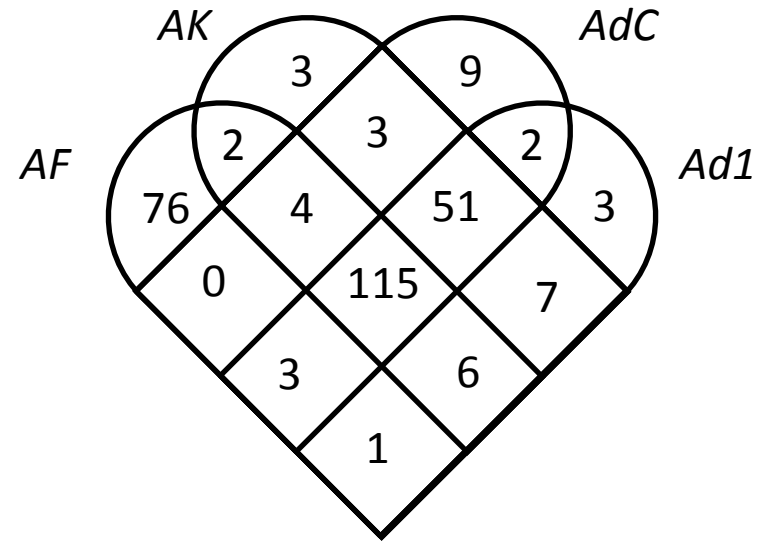

Additional Figure 2

Supplement: Additional file 2: Figure S2. — Orthology relationships within-family. Venn diagrams showing the distribution of orthologous genes between organisms. Some orthologous clusters exhibited multiple members from the same organism, suggestive of lineage-specific gene duplications. For the purpose of these diagrams, duplicated proteins were treated as singletons. For instance, if a cluster contained members from Sa, Cc, Mx and Mf, but the Sa protein was duplicated, then 1 was entered in the Sa/Cc/Mx/Mf cell, and 1 into the Sa only cell. For a pair of Sa proteins, 2 was entered into the Sa cell. a: Sa/Cc/Mx/Mf, b: AF/AK/Ad1/AdC. (PDF 185 kb) [file 12864_2015_2018_MOESM2_ESM.pdf]
