# Supplementary material for: Genome-wide analysis of myxobacterial two-component systems: genome relatedness and evolutionary changes
Source: BMC Genomics. 2015 Oct 13;16:780. doi: 10.1186/s12864-015-2018-y (PMC4603909; doi:10.1186/s12864-015-2018-y)
Supplement: Additional file 3: — The sequence clusters of orthologues of every myxobacterial TCS protein. (DOC 279 kb) [file 12864_2015_2018_MOESM3_ESM.doc]

CD-HIT output for all myxobacterial TCS genes, with clustering cut-off 50%.

Clusters have not been subjected to manual curation.

Clusters exhibiting changes in gene organisation between *Mx*, *Mf*, *Cc* and *Sa* or between *Ad1*, *AdC*, *AK* and *AF* are indicated with a ‘$’ or an ‘£’ respectively.

Clusters exhibiting changes in protein size between *Mx*, *Mf*, *Cc* and *Sa* are indicated with a ‘&’.

Clusters containing duplications of Mx, Mf, Cc, Sa, Ad1, AdC, AK or AF genes are indicated with a ^

>Cluster 0$

0 226aa, >LILAB_31835||RR|OmpR|R|Pair|... at 56%

1 229aa, >LILAB_31875||RR|OmpR|R|Complex|... at 58%

2 226aa, >MXAN_4777|PhoP1|RR|OmpR|R|Pair|... at 56%

3 229aa, >MXAN_4787|PhoP4|RR|OmpR|R|Complex|... at 58%

4 237aa, >COCOR_02611||RR|OmpR|R|Orphan|... at 56%

5 226aa, >COCOR_02620||RR|OmpR|R|Pair|... at 56%

6 226aa, >STAUR_5625||RR|OmpR|R|Complex|... at 57%

7 229aa, >STAUR_5628||RR|OmpR|R|Complex|... at 61%

8 230aa, >A2cp1_4151||RR|OmpR|R|Pair|... at 59%

9 230aa, >Adeh_4009||RR|OmpR|R|Pair|... at 56%

10 230aa, >AnaeK_4125||RR|OmpR|R|Pair|... at 58%

11 231aa, >Anae109_0415||RR|OmpR|R|Pair|... at 58%

12 256aa, >PPSIR1_08142||RR|OmpR|R||... *

13 243aa, >Hoch_6002||RR|OmpR|R|Pair|... at 65%

14 229aa, >A176_5973||RR|OmpR|R||... at 57%

15 226aa, >A176_5983||RR|OmpR|R||... at 56%

16 231aa, >sce2948||RR|OmpR|R|Pair|... at 58%

>Cluster 1&

0 2151aa, >LILAB_05220||HK|Hybrid|TRRR|Complex|... at 56%

1 3669aa, >LILAB_12790||HK|Hybrid|TRRR|Complex|... *

2 1967aa, >MXAN_0712||HK|Hybrid|TRRR|Complex|... at 55%

3 2478aa, >MXAN_6735||HK|Hybrid|TRRR|Complex|... at 79%

4 2475aa, >COCOR_07311||HK|Hybrid|TRRR|Complex|... at 74%

5 2483aa, >STAUR_1169||HK|Hybrid|TRRR|Complex|... at 73%

6 2142aa, >STAUR_7575||HK|Hybrid|TRRR|Complex|... at 57%

7 1847aa, >A2cp1_4276||HK|Hybrid|TR|Complex|... at 54%

8 1853aa, >Adeh_4122||HK|Hybrid|TR|Complex|... at 53%

9 1843aa, >AnaeK_4253||HK|Hybrid|TR|Complex|... at 54%

10 1816aa, >Anae109_0540||HK|Hybrid|TR|Complex|... at 50%

11 1869aa, >Anae109_2236A||HK|Hybrid|TR|Complex|... at 51%

12 2107aa, >Hoch_2076||HK|Hybrid|TRRR|Complex|... at 59%

13 1364aa, >A176_0388||HK|Hybrid|TRRR||... at 94%

14 1650aa, >A176_3398||HK|Hybrid|TRRR||... at 56%

>Cluster 2

0 187aa, >LILAB_11515||RR|PrrA|R|Pair|... *

1 185aa, >LILAB_15315||RR|PrrA|R|Pair|... at 58%

2 183aa, >MXAN_6224||RR|PrrA|R|Pair|... at 58%

3 187aa, >MXAN_6980||RR|PrrA|R|Pair|... at 94%

4 185aa, >COCOR_07568||RR|PrrA|R|Pair|... at 83%

5 185aa, >STAUR_1187||RR|PrrA|R|Pair|... at 73%

6 185aa, >A2cp1_2343||RR|PrrA|R|Pair|... at 63%

7 185aa, >Adeh_1607||RR|PrrA|R|Pair|... at 63%

8 185aa, >AnaeK_2255||RR|PrrA|R|Pair|... at 63%

9 182aa, >Anae109_2205||RR|PrrA|R|Pair|... at 63%

10 184aa, >PPSIR1_09420||RR|PrrA|R||... at 51%

11 180aa, >Hoch_5543||RR|PrrA|R|Pair|... at 66%

12 184aa, >A176_2289||RR|PrrA|R||... at 58%

13 187aa, >A176_5496||RR|PrrA|R||... at 95%

14 182aa, >sce8051||RR|PrrA|R|Pair|... at 50%

>Cluster 3

0 471aa, >LILAB_29335||RR|NtrC|R|Orphan|... at 72%

1 471aa, >MXAN_4240|Nla22|RR|NtrC|R|Orphan|... at 71%

2 466aa, >A2cp1_0864||RR|NtrC|R|Orphan|... at 60%

3 483aa, >A2cp1_2629||RR|NtrC|R|Complex|... at 99%

4 466aa, >Adeh_0812||RR|NtrC|R|Orphan|... at 59%

5 485aa, >Adeh_1326||RR|NtrC|R|Complex|... *

6 466aa, >AnaeK_0860||RR|NtrC|R|Orphan|... at 59%

7 483aa, >AnaeK_2533||RR|NtrC|R|Complex|... at 99%

8 464aa, >Anae109_0852||RR|PrrA|R|Orphan|... at 56%

9 480aa, >Anae109_2442||RR|NtrC|R|Complex|... at 92%

10 471aa, >A176_4102||RR|NtrC|R||... at 71%

11 475aa, >sce5791||RR|NtrC|R|Orphan|... at 52%

12 464aa, >sce6014||RR|NtrC|R|Complex|... at 51%

>Cluster 4

0 468aa, >LILAB_20255||RR|NtrC|R|Orphan|... at 61%

1 468aa, >MXAN_2516|Nla4|RR|NtrC|R|Orphan|... at 61%

2 468aa, >COCOR_05573||RR|NtrC|R|Orphan|... at 61%

3 472aa, >STAUR_3197||RR|NtrC|R|Orphan|... at 62%

4 474aa, >A2cp1_0723||RR|NtrC|R|Orphan|... at 59%

5 476aa, >Adeh_0688||RR|NtrC|R|Orphan|... at 58%

6 475aa, >AnaeK_0722||RR|NtrC|R|Orphan|... at 57%

7 471aa, >Anae109_0732||RR|NtrC|R|Orphan|... at 55%

8 475aa, >PPSIR1_25876||RR|NtrC|R||... at 57%

9 501aa, >Hoch_2606||RR|NtrC|R|Orphan|... *

10 474aa, >A176_4694||RR|NtrC|R||... at 60%

11 468aa, >sce9222||RR|NtrC|R|Orphan|... at 58%

>Cluster 5

0 478aa, >LILAB_36410||RR|NtrC|R|Pair|... at 50%

1 478aa, >MXAN_5784|PilR|RR|NtrC|R|Pair|... at 50%

2 477aa, >COCOR_06284||RR|NtrC|R|Pair|... at 50%

3 454aa, >STAUR_6454||RR|NtrC|R|Pair|... at 50%

4 461aa, >A2cp1_0660||RR|NtrC|R|Pair|... at 51%

5 460aa, >Adeh_0626||RR|NtrC|R|Pair|... at 50%

6 460aa, >AnaeK_0660||RR|NtrC|R|Pair|... at 51%

7 457aa, >Anae109_0671||RR|NtrC|R|Pair|... at 52%

8 466aa, >Hoch_4153||RR|NtrC|R|Orphan|... at 55%

9 456aa, >A176_4465||RR|NtrC|R||... at 52%

10 493aa, >sce4272||RR|NtrC|R|Orphan|... *

>Cluster 6$

0 441aa, >LILAB_04015||RR|NtrC|R|Pair|... at 59%

1 455aa, >MXAN_0937|Nla7|RR|NtrC|R|Pair|... at 59%

2 462aa, >COCOR_07531||RR|NtrC|R|Complex|... at 58%

3 456aa, >STAUR_4537||RR|NtrC|R|Complex|... at 57%

4 466aa, >PPSIR1_07485||RR|NtrC|R||... at 53%

5 471aa, >PPSIR1_42124||RR|NtrC|R||... at 53%

6 453aa, >Hoch_2280||RR|NtrC|R|Complex|... at 57%

7 442aa, >A176_0946||RR|NtrC|R||... at 59%

8 460aa, >sce6966||RR|NtrC|R|Complex|... at 56%

9 461aa, >sce7801||RR|NtrC|R|Pair|... at 62%

10 475aa, >sce8721||RR|NtrC|R|Complex|... *

>Cluster 7

0 474aa, >LILAB_36375||RR|NtrC|R|Pair|... *

1 474aa, >MXAN_5777|Nla23|RR|NtrC|R|Pair|... at 94%

2 470aa, >COCOR_06277||RR|NtrC|R|Pair|... at 81%

3 467aa, >STAUR_6446||RR|NtrC|R|Pair|... at 82%

4 458aa, >A2cp1_0673||RR|NtrC|R|Pair|... at 60%

5 455aa, >Adeh_0639||RR|NtrC|R|Pair|... at 61%

6 457aa, >AnaeK_0673||RR|NtrC|R|Pair|... at 61%

7 456aa, >Anae109_0684||RR|NtrC|R|Pair|... at 61%

8 471aa, >PPSIR1_35422||RR|NtrC|R||... at 51%

9 472aa, >A176_4472||RR|NtrC|R||... at 87%

10 453aa, >sce2881||RR|NtrC|R|Orphan|... at 52%

>Cluster 8

0 125aa, >LILAB_00995||RR|CheY|R|Pair|... at 62%

1 153aa, >LILAB_06225||RR|CheY|R|Orphan|... at 94%

2 169aa, >MXAN_0524||RR|CheY|R|Orphan|... *

3 136aa, >MXAN_1552||RR|CheY|R|Pair|... at 58%

4 156aa, >COCOR_00459||RR|CheY|R|Orphan|... at 75%

5 136aa, >COCOR_01560||RR|CheY|R|Pair|... at 57%

6 132aa, >STAUR_2325||RR|CheY|R|Pair|... at 60%

7 150aa, >STAUR_7845||RR|CheY|R|Orphan|... at 70%

8 125aa, >A176_3850||RR|CheY|R||... at 60%

9 153aa, >A176_1405||RR|CheY|R||... at 93%

10 142aa, >sce2954||RR|CheY|R|Complex|... at 50%

>Cluster 9

0 469aa, >LILAB_02505||RR|NtrC|R|Orphan|... at 63%

1 477aa, >MXAN_1245|SasR|RR|NtrC|R|Orphan|... at 63%

2 469aa, >COCOR_01156||RR|NtrC|R|Orphan|... at 64%

3 469aa, >STAUR_1862||RR|NtrC|R|Orphan|... at 62%

4 469aa, >A2cp1_4396||RR|NtrC|R|Orphan|... at 83%

5 469aa, >Adeh_4240||RR|NtrC|R|Orphan|... at 83%

6 469aa, >AnaeK_4373||RR|NtrC|R|Orphan|... at 83%

7 495aa, >Anae109_4389||RR|NtrC|R|Orphan|... *

8 459aa, >Hoch_5989||RR|NtrC|R|Pair|... at 58%

9 478aa, >A176_0556||RR|NtrC|R||... at 62%

>Cluster 10

0 463aa, >LILAB_03245||RR|NtrC|R|Pair|... at 85%

1 482aa, >MXAN_1078|SpdR|RR|NtrC|R|Pair|... at 85%

2 484aa, >COCOR_01018||RR|NtrC|R|Pair|... *

3 476aa, >STAUR_1639||RR|NtrC|R|Pair|... at 82%

4 481aa, >A2cp1_0446||RR|NtrC|R|Complex|... at 52%

5 474aa, >Adeh_0417||RR|NtrC|R|Complex|... at 52%

6 481aa, >AnaeK_0445||RR|NtrC|R|Complex|... at 52%

7 480aa, >Anae109_4152||RR|NtrC|R|Complex|... at 50%

8 467aa, >A176_4006||RR|NtrC|R||... at 84%

9 451aa, >sce1649||RR|NtrC|R|Complex|... at 51%

>Cluster 11

0 474aa, >LILAB_25760||RR|NtrC|R|Orphan|... at 84%

1 474aa, >MXAN_3555||RR|NtrC|R|Orphan|... at 84%

2 474aa, >COCOR_04484||RR|NtrC|R|Orphan|... at 84%

3 478aa, >STAUR_4072||RR|NtrC|R|Orphan|... *

4 473aa, >A2cp1_2056||RR|NtrC|R|Pair|... at 65%

5 473aa, >Adeh_1908||RR|NtrC|R|Pair|... at 65%

6 473aa, >AnaeK_1971||RR|NtrC|R|Pair|... at 65%

7 470aa, >Anae109_1951||RR|NtrC|R|Pair|... at 67%

8 472aa, >Hoch_2510||RR|NtrC|R|Orphan|... at 58%

9 474aa, >A176_6908||RR|NtrC|R||... at 84%

>Cluster 12

0 349aa, >A2cp1_0625||RR|CheB|R|Orphan|... at 61%

1 362aa, >A2cp1_2570||RR|CheB|R|Complex|... *

2 356aa, >A2cp1_2580||RR|CheB|R|Orphan|... at 55%

3 349aa, >Adeh_0600||RR|CheB|R|Orphan|... at 60%

4 356aa, >Adeh_1373||RR|CheB|R|Orphan|... at 55%

5 362aa, >Adeh_1383||RR|CheB|R|Orphan|... at 94%

6 349aa, >AnaeK_0634||RR|CheB|R|Orphan|... at 61%

7 362aa, >AnaeK_2474||RR|CheB|R|Orphan|... at 98%

8 356aa, >AnaeK_2484||RR|CheB|R|Orphan|... at 56%

9 358aa, >Anae109_0644||RR|CheB|R|Orphan|... at 62%

>Cluster 13$

0 233aa, >LILAB_14490||RR|OmpR|R|Pair|... at 65%

1 238aa, >LILAB_21595||RR|OmpR|R|Pair|... at 79%

2 238aa, >MXAN_2778|PhoP2|RR|OmpR|R|Pair|... at 80%

3 233aa, >MXAN_6413|PhoP3|RR|OmpR|R|Pair|... at 64%

4 234aa, >COCOR_01404||RR|OmpR|R|Pair|... at 63%

5 241aa, >COCOR_05279||RR|OmpR|R|Pair|... *

6 235aa, >STAUR_0727||RR|OmpR|R|Pair|... at 58%

7 241aa, >STAUR_4909||RR|OmpR|R|Complex|... at 73%

8 233aa, >A176_2487||RR|OmpR|R||... at 64%

9 238aa, >A176_0216||RR|OmpR|R||... at 77%

>Cluster 14

0 126aa, >LILAB_31165||RR|CheY|R|Orphan|... at 59%

1 134aa, >MXAN_4645||RR|CheY|R|Orphan|... at 55%

2 129aa, >COCOR_04849||RR|CheY|R|Orphan|... at 51%

3 132aa, >STAUR_5427||RR|CheY|R|Orphan|... at 51%

4 132aa, >A2cp1_1223||RR|CheY|R|Orphan|... at 56%

5 138aa, >Adeh_1095||RR|CheY|R|Orphan|... at 53%

6 132aa, >AnaeK_1155||RR|CheY|R|Orphan|... at 56%

7 138aa, >Anae109_1134||RR|CheY|R|Orphan|... at 53%

8 168aa, >Hoch_5910||RR|CheY|R|Orphan|... *

9 126aa, >A176_1966||RR|CheY|R||... at 57%

>Cluster 15$

0 134aa, >LILAB_05130||RR|CheY|R|Complex|... at 67%

1 136aa, >LILAB_05155||RR|CheY|R|Complex|... at 86%

2 165aa, >MXAN_0726||RR|CheY|R|Complex|... *

3 136aa, >MXAN_0732||RR|CheY|R|Complex|... at 66%

4 131aa, >COCOR_00604||RR|CheY|R|Orphan|... at 73%

5 139aa, >COCOR_00614||RR|CheY|R|Complex|... at 63%

6 132aa, >STAUR_7557||RR|CheY|R|Complex|... at 62%

7 140aa, >STAUR_7565||RR|CheY|R|Complex|... at 70%

8 136aa, >A176_3380||RR|CheY|R||... at 66%

9 146aa, >A176_3385||RR|CheY|R||... at 82%

>Cluster 16$

0 607aa, >LILAB_14485||HK|Classic|T|Pair|... at 69%

1 609aa, >LILAB_21600||HK|Classic|T|Pair|... at 50%

2 609aa, >MXAN_2779|PhoR2|HK|Classic|T|Pair|... at 50%

3 607aa, >MXAN_6414|PhoR3|HK|Classic|T|Pair|... at 69%

4 614aa, >COCOR_01405||HK|Classic|T|Pair|... *

5 609aa, >COCOR_05278||HK|Classic|T|Pair|... at 50%

6 609aa, >STAUR_4908||HK|Classic|T|Complex|... at 51%

7 607aa, >A176_2488||HK|Classic|T||... at 69%

8 609aa, >A176_0217||HK|Classic|T||... at 50%

>Cluster 17

0 485aa, >LILAB_34650||RR|NtrC|R|Complex|... at 93%

1 485aa, >MXAN_5364|HsfA|RR|NtrC|R|Complex|... at 94%

2 491aa, >COCOR_02206||RR|NtrC|R|Complex|... *

3 484aa, >STAUR_6071||RR|NtrC|R|Complex|... at 89%

4 489aa, >A2cp1_3623||RR|NtrC|R|Complex|... at 55%

5 489aa, >Adeh_3471||RR|NtrC|R|Complex|... at 57%

6 489aa, >AnaeK_3555||RR|NtrC|R|Complex|... at 55%

7 490aa, >Anae109_3581||RR|NtrC|R|Complex|... at 56%

8 485aa, >A176_7543||RR|NtrC|R||... at 94%

>Cluster 18

0 484aa, >LILAB_28325||RR|NtrC|R|Complex|... *

1 484aa, >MXAN_4042|Nla6|RR|NtrC|R|Pair|... at 99%

2 479aa, >COCOR_03899||RR|NtrC|R|Pair|... at 92%

3 457aa, >STAUR_4506||RR|NtrC|R|Pair|... at 87%

4 469aa, >A2cp1_2398||RR|NtrC|R|Complex|... at 66%

5 467aa, >Adeh_1555||RR|NtrC|R|Complex|... at 66%

6 469aa, >AnaeK_2310||RR|NtrC|R|Complex|... at 66%

7 468aa, >Anae109_2258||RR|NtrC|R|Complex|... at 65%

8 477aa, >A176_1744||RR|NtrC|R||... at 97%

>Cluster 19&

0 165aa, >LILAB_30440||RR|CheY|R|Complex|... at 58%

1 420aa, >MXAN_4461|RomR|RR|unclassified|R|Complex|... at 51%

2 421aa, >COCOR_03318||RR|CheY|R|Complex|... at 51%

3 413aa, >STAUR_4816||RR|CheY|R|Complex|... at 52%

4 476aa, >A2cp1_1568||RR|unclassified|R|Complex|... at 97%

5 469aa, >Adeh_2391||RR|unclassified|R|Complex|... at 92%

6 477aa, >AnaeK_1473||RR|unclassified|R|Complex|... *

7 453aa, >Anae109_1476||RR|unclassified|R|Complex|... at 56%

8 420aa, >A176_5137||RR|CheY|R||... at 52%

>Cluster 20

0 439aa, >LILAB_13380||RR|unclassified|R|Orphan|... at 98%

1 470aa, >MXAN_6627||RR|unclassified|R|Orphan|... *

2 440aa, >COCOR_07185||RR|CheY|R|Orphan|... at 90%

3 441aa, >STAUR_1269||RR|CheY|R|Orphan|... at 85%

4 451aa, >A2cp1_4417||RR|unclassified|R|Orphan|... at 51%

5 451aa, >Adeh_4262||RR|unclassified|R|Orphan|... at 50%

6 469aa, >AnaeK_4396||RR|unclassified|R|Orphan|... at 50%

7 442aa, >Anae109_4417||RR|unclassified|R|Orphan|... at 54%

8 439aa, >A176_2168||RR|CheY|R||... at 95%

>Cluster 21

0 457aa, >LILAB_30450||RR|PleD|R|Complex|... at 52%

1 457aa, >MXAN_4463||RR|PleD|R|Complex|... at 52%

2 462aa, >COCOR_03316||RR|PleD|R|Complex|... at 51%

3 453aa, >STAUR_4818||RR|PleD|R|Complex|... at 53%

4 455aa, >A2cp1_1566||RR|PleD|R|Complex|... at 71%

5 455aa, >Adeh_2393||RR|PleD|R|Complex|... at 71%

6 455aa, >AnaeK_1471||RR|PleD|R|Complex|... at 71%

7 465aa, >Anae109_1474||RR|PleD|R|Complex|... *

8 457aa, >A176_5139||RR|PleD|R||... at 51%

>Cluster 22$

0 454aa, >LILAB_29465||RR|NtrC|R|Complex|... at 74%

1 439aa, >LILAB_30920||RR|NtrC|R|Orphan|... at 96%

2 452aa, >MXAN_4261||RR|NtrC|R|Complex|... at 75%

3 457aa, >MXAN_4580|Nla8|RR|NtrC|R|Pair|... *

4 457aa, >COCOR_03585||RR|NtrC|R|Pair|... at 77%

5 454aa, >COCOR_03610||RR|NtrC|R|Pair|... at 73%

6 448aa, >STAUR_4861||RR|NtrC|R|Pair|... at 74%

7 452aa, >A176_4135||RR|NtrC|R||... at 74%

8 448aa, >A176_1909||RR|NtrC|R||... at 95%

>Cluster 23

0 411aa, >LILAB_29300||RR|RpfG|R|Orphan|... *

1 411aa, >MXAN_4232||RR|RpfG|R|Orphan|... at 97%

2 410aa, >COCOR_03704||RR|RpfG|R|Orphan|... at 89%

3 399aa, >STAUR_4687||RR|RpfG|R|Orphan|... at 86%

4 357aa, >A2cp1_2101||RR|RpfG|R|Orphan|... at 62%

5 357aa, >Adeh_1829||RR|RpfG|R|Orphan|... at 62%

6 357aa, >AnaeK_2031||RR|RpfG|R|Orphan|... at 62%

7 357aa, >Anae109_1991||RR|RpfG|R|Orphan|... at 64%

8 410aa, >A176_4095||RR|RpfG|R||... at 98%

>Cluster 24

0 371aa, >LILAB_31315||RR|RpfG|R|Orphan|... at 53%

1 371aa, >MXAN_4675||RR|RpfG|R|Orphan|... at 53%

2 373aa, >COCOR_04872||RR|RpfG|R|Orphan|... at 54%

3 369aa, >STAUR_5467||RR|RpfG|R|Orphan|... at 52%

4 375aa, >A2cp1_1265||RR|RpfG|R|Orphan|... at 78%

5 391aa, >Adeh_1136||RR|RpfG|R|Orphan|... at 75%

6 375aa, >AnaeK_1196||RR|RpfG|R|Orphan|... at 78%

7 394aa, >Anae109_1184||RR|RpfG|R|Orphan|... *

8 371aa, >A176_1997||RR|RpfG|R||... at 53%

>Cluster 25

0 233aa, >LILAB_02055||RR|OmpR|R|Pair|... at 86%

1 232aa, >MXAN_1349||RR|OmpR|R|Pair|... at 85%

2 237aa, >COCOR_01238||RR|OmpR|R|Pair|... *

3 229aa, >STAUR_2102||RR|OmpR|R|Pair|... at 82%

4 230aa, >A2cp1_0440||RR|OmpR|R|Pair|... at 52%

5 230aa, >Adeh_0411||RR|OmpR|R|Pair|... at 52%

6 230aa, >AnaeK_0439||RR|OmpR|R|Pair|... at 51%

7 230aa, >Anae109_4159||RR|OmpR|R|Pair|... at 50%

8 232aa, >A176_0643||RR|OmpR|R||... at 86%

>Cluster 26

0 122aa, >LILAB_12995||RR|CheY|R|Pair|... at 100%

1 122aa, >MXAN_6693|DifD|RR|CheY|R|Pair|... at 100%

2 122aa, >COCOR_07284||RR|CheY|R|Pair|... at 98%

3 122aa, >STAUR_1220||RR|CheY|R|Pair|... at 95%

4 122aa, >A2cp1_4472||RR|CheY|R|Pair|... at 88%

5 122aa, >Adeh_4317||RR|CheY|R|Pair|... at 88%

6 122aa, >AnaeK_4453||RR|CheY|R|Pair|... at 88%

7 122aa, >Anae109_4462||RR|CheY|R|Pair|... at 86%

8 136aa, >A176_2234||RR|CheY|R||... *

>Cluster 27£

0 135aa, >LILAB_22540||RR|CheY|R|Pair|... *

1 125aa, >MXAN_2962||RR|CheY|R|Pair|... at 97%

2 124aa, >COCOR_05100||RR|CheY|R|Pair|... at 86%

3 127aa, >STAUR_5149||RR|CheY|R|Pair|... at 82%

4 126aa, >A2cp1_1974||RR|CheY|R|Pair|... at 53%

5 126aa, >Adeh_1989||RR|CheY|R|Orphan|... at 52%

6 126aa, >AnaeK_1889||RR|CheY|R|Complex|... at 53%

7 126aa, >Anae109_1850||RR|CheY|R|Complex|... at 57%

8 135aa, >A176_4867||RR|CheY|R||... at 96%

>Cluster 28

0 127aa, >LILAB_35915||RR|CheY|R|Orphan|... at 96%

1 127aa, >MXAN_5688||RR|CheY|R|Orphan|... at 96%

2 128aa, >COCOR_06169||RR|CheY|R|Orphan|... *

3 127aa, >STAUR_6354||RR|CheY|R|Orphan|... at 91%

4 127aa, >A2cp1_0641||RR|CheY|R|Complex|... at 57%

5 127aa, >Adeh_0616||RR|CheY|R|Complex|... at 57%

6 127aa, >AnaeK_0650||RR|CheY|R|Complex|... at 57%

7 127aa, >Anae109_0660||RR|CheY|R|Complex|... at 56%

8 127aa, >A176_7176||RR|CheY|R||... at 96%

>Cluster 29$

0 702aa, >LILAB_29470||HK|Classic|T|Complex|... at 58%

1 702aa, >MXAN_4262||HK|Classic|T|Complex|... at 57%

2 712aa, >MXAN_4579||HK|Classic|T|Pair|... at 86%

3 705aa, >COCOR_03586||HK|Classic|T|Pair|... at 58%

4 710aa, >COCOR_03609||HK|Classic|T|Pair|... at 57%

5 713aa, >STAUR_4862||HK|Classic|T|Pair|... at 58%

6 705aa, >A176_4136||HK|Classic|T||... at 56%

7 737aa, >A176_1908||HK|Classic|T||... *

>Cluster 30

0 540aa, >A2cp1_1802||HK|Classic|T|Pair|... at 57%

1 578aa, >A2cp1_1854||HK|Classic|T|Pair|... at 53%

2 599aa, >A2cp1_2175||HK|Classic|T|Pair|... *

3 596aa, >Adeh_1759||HK|Classic|T|Pair|... at 89%

4 575aa, >Adeh_2102||HK|Classic|T|Pair|... at 53%

5 560aa, >AnaeK_1728||HK|Classic|T|Pair|... at 55%

6 578aa, >AnaeK_1774||HK|Classic|T|Pair|... at 53%

7 597aa, >AnaeK_2081||HK|Classic|T|Pair|... at 98%

>Cluster 31$

0 466aa, >LILAB_06400||HK|Classic|T|Complex|... at 53%

1 471aa, >LILAB_22930||HK|Classic|T|Orphan|... at 94%

2 469aa, >MXAN_0459|RedC|HK|Classic|T|Complex|... at 52%

3 514aa, >MXAN_3036||HK|Classic|T|Orphan|... *

4 478aa, >COCOR_02335||HK|Classic|T|Orphan|... at 51%

5 471aa, >STAUR_7971||HK|Classic|T|Pair|... at 51%

6 507aa, >A176_5883||HK|Classic|T||... at 88%

7 465aa, >A176_1437||HK|Classic|T||... at 52%

>Cluster 32

0 471aa, >A2cp1_1801||RR|NtrC|R|Pair|... at 69%

1 469aa, >A2cp1_1855||RR|NtrC|R|Pair|... at 68%

2 478aa, >A2cp1_2176||RR|NtrC|R|Pair|... *

3 478aa, >Adeh_1758||RR|NtrC|R|Pair|... at 95%

4 469aa, >Adeh_2101||RR|NtrC|R|Pair|... at 68%

5 471aa, >AnaeK_1727||RR|NtrC|R|Pair|... at 69%

6 469aa, >AnaeK_1775||RR|NtrC|R|Pair|... at 68%

7 478aa, >AnaeK_2082||RR|NtrC|R|Pair|... at 99%

>Cluster 33

0 477aa, >MXAN_3811|Nla13|RR|NtrC|R|Pair|... *

1 453aa, >COCOR_05933||RR|NtrC|R|Pair|... at 69%

2 461aa, >STAUR_0772||RR|NtrC|R|Pair|... at 55%

3 466aa, >A2cp1_0883||RR|NtrC|R|Pair|... at 68%

4 466aa, >Adeh_0833||RR|NtrC|R|Pair|... at 68%

5 466aa, >AnaeK_0879||RR|NtrC|R|Pair|... at 68%

6 457aa, >Anae109_0889||RR|NtrC|R|Pair|... at 70%

7 467aa, >A176_5280||RR|NtrC|R||... at 88%

>Cluster 34

0 353aa, >MXAN_6952||RR|CheB|R|Complex|... at 69%

1 355aa, >COCOR_07522||RR|CheB|R|Complex|... at 69%

2 361aa, >STAUR_0870||RR|CheB|R|Complex|... *

3 342aa, >A2cp1_2774||RR|CheB|R|Complex|... at 59%

4 342aa, >Adeh_1192||RR|CheB|R|Complex|... at 57%

5 342aa, >AnaeK_2679||RR|CheB|R|Complex|... at 58%

6 338aa, >Anae109_2298||RR|CheB|R|Complex|... at 54%

7 353aa, >A176_5467||RR|CheB|R||... at 68%

>Cluster 35£^

0 245aa, >COCOR_07821||RR|OmpR|R|Pair|... *

1 241aa, >STAUR_7042||RR|OmpR|R|Pair|... at 77%

2 234aa, >A2cp1_3106||RR|OmpR|R|Pair|... at 62%

3 238aa, >A2cp1_3138||RR|OmpR|R|Complex|... at 65%

4 245aa, >Adeh_2914||RR|OmpR|R|Pair|... at 59%

5 234aa, >AnaeK_3000||RR|OmpR|R|Pair|... at 62%

6 234aa, >Anae109_0880||RR|OmpR|R|Pair|... at 62%

7 228aa, >sce2049||RR|OmpR|R|Complex|... at 50%

>Cluster 36

0 126aa, >LILAB_31705||RR|CheY|R|Complex|... at 53%

1 126aa, >MXAN_4751||RR|CheY|R|Complex|... at 53%

2 127aa, >COCOR_02645||RR|CheY|R|Pair|... at 51%

3 125aa, >STAUR_5601||RR|CheY|R|Pair|... at 51%

4 128aa, >A2cp1_2913||RR|CheY|R|Complex|... *

5 128aa, >Adeh_2729||RR|CheY|R|Complex|... at 90%

6 128aa, >AnaeK_2821||RR|CheY|R|Complex|... at 97%

7 126aa, >A176_6010||RR|CheY|R||... at 55%

>Cluster 37$^

0 559aa, >LILAB_28365A||RR|unclassified|R|Pair|... at 77%

1 689aa, >MXAN_4049||RR|CyC-C|R|Orphan|... *

2 672aa, >COCOR_05744||RR|CyC-C|R|Orphan|... at 55%

3 663aa, >STAUR_0029||RR|CyC-C|R|Pair|... at 54%

4 665aa, >STAUR_4565||RR|CyC-C|R|Orphan|... at 51%

5 679aa, >STAUR_7210||RR|CyC-C|R|Orphan|... at 55%

6 674aa, >A176_1751||RR|CyC-C|R||... at 85%

>Cluster 38

0 560aa, >A2cp1_1952||HK|Classic|T|Pair|... at 98%

1 548aa, >A2cp1_3530||HK|Classic|T|Orphan|... at 50%

2 584aa, >Adeh_2011||HK|Classic|T|Pair|... at 95%

3 548aa, >Adeh_3383||HK|Classic|T|Orphan|... at 50%

4 586aa, >AnaeK_1867||HK|Classic|T|Pair|... *

5 548aa, >AnaeK_3466||HK|Classic|T|Orphan|... at 50%

6 584aa, >Anae109_1796||HK|Classic|T|Pair|... at 73%

>Cluster 39

0 463aa, >A2cp1_1708||RR|NtrC|R|Pair|... at 96%

1 448aa, >A2cp1_2499||RR|NtrC|R|Complex|... at 69%

2 448aa, >Adeh_1450||RR|NtrC|R|Pair|... at 69%

3 466aa, >Adeh_2239||RR|NtrC|R|Pair|... *

4 463aa, >AnaeK_1635||RR|NtrC|R|Pair|... at 95%

5 448aa, >AnaeK_2412||RR|NtrC|R|Pair|... at 69%

6 460aa, >Anae109_1017||RR|NtrC|R|Pair|... at 78%

>Cluster 40

0 454aa, >A2cp1_0884||HK|Classic|T|Pair|... at 59%

1 449aa, >A2cp1_3303||HK|Classic|T|Orphan|... at 53%

2 439aa, >Adeh_0834||HK|Classic|T|Pair|... at 62%

3 448aa, >Adeh_3109||HK|Classic|T|Orphan|... at 55%

4 439aa, >AnaeK_0880||HK|Classic|T|Pair|... at 61%

5 449aa, >AnaeK_3218||HK|Classic|T|Orphan|... at 54%

6 458aa, >Anae109_0890||HK|Classic|T|Pair|... *

>Cluster 41

0 442aa, >LILAB_11520||HK|Classic|T|Pair|... at 52%

1 436aa, >MXAN_6979||HK|Classic|T|Pair|... at 51%

2 452aa, >A2cp1_2344||HK|Classic|T|Pair|... at 96%

3 457aa, >Adeh_1606||HK|Classic|T|Pair|... *

4 452aa, >AnaeK_2256||HK|Classic|T|Pair|... at 96%

5 429aa, >Anae109_2206||HK|Classic|T|Pair|... at 68%

6 442aa, >A176_5495||HK|Classic|T||... at 51%

>Cluster 42

0 396aa, >LILAB_29365||HK|Classic|T|Complex|... *

1 396aa, >MXAN_4246||HK|Classic|T|Complex|... at 92%

2 379aa, >A2cp1_2626||HK|Classic|T|Complex|... at 64%

3 379aa, >Adeh_1329||HK|Classic|T|Complex|... at 64%

4 379aa, >AnaeK_2530||HK|Classic|T|Complex|... at 64%

5 382aa, >Anae109_2438||HK|Classic|T|Complex|... at 65%

6 396aa, >A176_4108||HK|Classic|T||... at 93%

>Cluster 43

0 368aa, >LILAB_29355||HK|Hybrid|RT|Complex|... at 70%

1 368aa, >MXAN_4244||HK|Hybrid|RT|Complex|... at 69%

2 375aa, >A2cp1_2628||HK|Hybrid|RT|Complex|... *

3 375aa, >Adeh_1327||HK|Hybrid|RT|Complex|... at 97%

4 375aa, >AnaeK_2532||HK|Hybrid|RT|Complex|... at 99%

5 375aa, >Anae109_2440||HK|Hybrid|RT|Complex|... at 77%

6 368aa, >A176_4106||HK|Hybrid|RT||... at 69%

>Cluster 44

0 237aa, >LILAB_29360||RR|FrzZ|RR|Complex|... at 61%

1 237aa, >MXAN_4245||RR|FrzZ|RR|Complex|... at 60%

2 240aa, >A2cp1_2627||RR|FrzZ|RR|Complex|... *

3 240aa, >Adeh_1328||RR|FrzZ|RR|Complex|... at 97%

4 240aa, >AnaeK_2531||RR|FrzZ|RR|Complex|... at 97%

5 238aa, >Anae109_2439||RR|FrzZ|RR|Complex|... at 74%

6 237aa, >A176_4107||RR|FrzZ|RR||... at 62%

>Cluster 45

0 226aa, >LILAB_25010||RR|OmpR|R|Pair|... at 58%

1 226aa, >MXAN_3450||RR|OmpR|R|Pair|... at 58%

2 218aa, >STAUR_4618||RR|OmpR|R|Pair|... at 58%

3 227aa, >A2cp1_3997||RR|OmpR|R|Pair|... *

4 227aa, >Adeh_3856||RR|OmpR|R|Pair|... at 97%

5 227aa, >AnaeK_3919||RR|OmpR|R|Pair|... at 99%

6 227aa, >A176_5033||RR|OmpR|R||... at 58%

>Cluster 46^

0 127aa, >LILAB_21115||RR|CheY|R|Complex|... at 88%

1 127aa, >MXAN_2684|CheY4|RR|CheY|R|Complex|... at 89%

2 137aa, >COCOR_05358||RR|CheY|R|Complex|... *

3 128aa, >STAUR_3421||RR|CheY|R|Complex|... at 78%

4 124aa, >Anae109_0467||RR|CheY|R|Complex|... at 64%

5 134aa, >Anae109_3535||RR|CheY|R|Complex|... at 52%

6 123aa, >A176_0126||RR|CheY|R||... at 90%

>Cluster 47

0 2031aa, >sce3497||HK|Classic|T|Orphan|... *

1 2019aa, >sce5443||HK|Classic|T|Complex|... at 73%

2 2013aa, >sce5444||HK|Classic|T|Complex|... at 71%

3 2010aa, >sce5445||HK|Classic|T|Complex|... at 66%

4 2012aa, >sce5446||HK|Classic|T|Complex|... at 60%

5 2018aa, >sce5457||HK|Classic|T|Orphan|... at 72%

>Cluster 48

0 1079aa, >A2cp1_0976||HK|Hybrid|TR|Pair|... *

1 462aa, >Adeh_0922||RR|NtrC|R|Pair|... at 98%

2 621aa, >Adeh_0923||HK|Classic|T|Pair|... at 90%

3 466aa, >AnaeK_0978||RR|NtrC|R|Pair|... at 99%

4 617aa, >AnaeK_0979||HK|Classic|T|Pair|... at 95%

5 466aa, >Anae109_0965||RR|NtrC|R|Pair|... at 87%

>Cluster 49^

0 508aa, >LILAB_07555||HK|Hybrid|TRT|Complex|... at 53%

1 508aa, >MXAN_0229||HK|Hybrid|RTR|Complex|... at 53%

2 525aa, >COCOR_07667||HK|Hybrid|RTR|Complex|... *

3 510aa, >COCOR_07814||HK|Hybrid|RTR|Complex|... at 54%

4 510aa, >STAUR_0288||HK|Hybrid|RTR|Complex|... at 55%

5 508aa, >A176_5372||HK|Hybrid|RTR||... at 55%

>Cluster 50^

0 480aa, >LILAB_09385||RR|CyC-C|R|Pair|... at 98%

1 480aa, >LILAB_09435||RR|CyC-C|R|Orphan|... at 98%

2 497aa, >MXAN_7396||RR|CyC-C|R|Pair|... *

3 486aa, >COCOR_07987||RR|CyC-C|R|Orphan|... at 90%

4 488aa, >STAUR_0316||RR|CyC-C|R|Orphan|... at 80%

5 486aa, >A176_3727||RR|CyC-C|R||... at 97%

>Cluster 51

0 466aa, >MXAN_3418||RR|NtrC|R|Pair|... at 62%

1 470aa, >COCOR_00920||RR|NtrC|R|Pair|... *

2 465aa, >A2cp1_0324||RR|NtrC|R|Pair|... at 64%

3 465aa, >Adeh_0302||RR|NtrC|R|Pair|... at 64%

4 465aa, >AnaeK_0313||RR|NtrC|R|Pair|... at 64%

5 463aa, >Anae109_4257||RR|NtrC|R|Pair|... at 62%

>Cluster 52£

0 462aa, >A2cp1_1707||HK|Classic|T|Pair|... *

1 247aa, >Adeh_0536||HK|Classic|T|Orphan|... at 56%

2 461aa, >Adeh_2240||HK|Classic|T|Pair|... at 96%

3 237aa, >AnaeK_0568||HK|Classic|T|Orphan|... at 60%

4 462aa, >AnaeK_1634||HK|Classic|T|Pair|... at 99%

5 441aa, >Anae109_1016||HK|Classic|T|Pair|... at 62%

>Cluster 53

0 455aa, >A2cp1_3483||RR|NtrC|R|Orphan|... at 56%

1 453aa, >Adeh_3338||RR|NtrC|R|Orphan|... at 56%

2 455aa, >AnaeK_3419||RR|NtrC|R|Orphan|... at 56%

3 455aa, >Anae109_3404||RR|NtrC|R|Orphan|... at 58%

4 461aa, >Hoch_0018||RR|NtrC|R|Orphan|... *

5 460aa, >sce1691||RR|NtrC|R|Complex|... at 52%

>Cluster 54$

0 447aa, >LILAB_31865||RR|NtrC|R|Complex|... at 66%

1 447aa, >MXAN_4785|Nla3|RR|NtrC|R|Complex|... at 68%

2 455aa, >COCOR_01047||RR|NtrC|R|Pair|... *

3 450aa, >COCOR_02119||RR|NtrC|R|Pair|... at 66%

4 447aa, >STAUR_6001||RR|NtrC|R|Pair|... at 67%

5 447aa, >A176_5975||RR|NtrC|R||... at 66%

>Cluster 55$

0 349aa, >LILAB_11610||RR|CheB|R|Orphan|... *

1 348aa, >MXAN_6959||RR|CheB|R|Orphan|... at 92%

2 343aa, >COCOR_07533||RR|CheB|R|Complex|... at 71%

3 345aa, >STAUR_0865||RR|CheB|R|Pair|... at 69%

4 340aa, >A176_5475||RR|CheB|R||... at 89%

5 344aa, >sce2662||RR|CheB|R|Orphan|... at 57%

>Cluster 56£

0 231aa, >A2cp1_0942||RR|OmpR|R|Pair|... at 58%

1 231aa, >Adeh_0891||RR|OmpR|R|Pair|... at 57%

2 231aa, >AnaeK_0939||RR|OmpR|R|Pair|... at 58%

3 233aa, >Anae109_3104||RR|OmpR|R|Complex|... at 60%

4 235aa, >PPSIR1_30140||RR|OmpR|R||... *

5 234aa, >sce1356||RR|OmpR|R|Pair|... at 61%

>Cluster 57

0 226aa, >LILAB_34400||RR|OmpR|R|Pair|... *

1 226aa, >MXAN_5313||RR|OmpR|R|Pair|... at 98%

2 226aa, >COCOR_02255||RR|OmpR|R|Pair|... at 88%

3 226aa, >STAUR_6024||RR|OmpR|R|Pair|... at 86%

4 223aa, >PPSIR1_27328||RR|OmpR|R||... at 68%

5 226aa, >A176_7492||RR|OmpR|R||... at 94%

>Cluster 58

0 197aa, >LILAB_35425||RR|Xre|R|Orphan|... at 100%

1 222aa, >MXAN_5592|DigR|RR|Xre|R|Orphan|... *

2 197aa, >COCOR_06083||RR|Xre|R|Orphan|... at 96%

3 197aa, >STAUR_6250||RR|Xre|R|Orphan|... at 96%

4 197aa, >A176_7074||RR|Xre|R||... at 99%

5 202aa, >sce0622||RR|Xre|R|Complex|... at 54%

>Cluster 59$

0 125aa, >LILAB_11580||RR|CheY|R|Complex|... at 82%

1 125aa, >MXAN_6965||RR|CheY|R|Complex|... at 80%

2 130aa, >COCOR_07539||RR|CheY|R|Complex|... *

3 127aa, >STAUR_0859||RR|CheY|R|Pair|... at 75%

4 125aa, >A176_5481||RR|CheY|R||... at 83%

5 112aa, >sce2656||RR|CheY|R|Complex|... at 66%

>Cluster 60

0 124aa, >STAUR_4724||RR|CheY|R|Pair|... *

1 123aa, >A2cp1_0631||RR|CheY|R|Orphan|... at 60%

2 123aa, >Adeh_0606||RR|CheY|R|Orphan|... at 59%

3 123aa, >AnaeK_0640||RR|CheY|R|Orphan|... at 59%

4 123aa, >Anae109_0650||RR|CheY|R|Orphan|... at 60%

5 124aa, >sce2206||RR|CheY|R|Orphan|... at 55%

>Cluster 61&^

0 1760aa, >STAUR_1021||HK|Classic|T|Orphan|... at 60%

1 419aa, >STAUR_1353||HK|Classic|T|Orphan|... at 83%

2 1761aa, >STAUR_5099||HK|Classic|T|Orphan|... at 73%

3 1763aa, >STAUR_7439||HK|Classic|T|Orphan|... *

4 1763aa, >STAUR_7639||HK|Classic|T|Orphan|... at 69%

>Cluster 62

0 1109aa, >LILAB_30360||HK|Hybrid|RTRR|Complex|... at 84%

1 1109aa, >MXAN_4445||HK|Hybrid|RTRR|Complex|... at 83%

2 1112aa, >COCOR_03338||HK|Hybrid|RTRR|Complex|... *

3 1097aa, >STAUR_4801||HK|Hybrid|RTRR|Complex|... at 77%

4 1108aa, >A176_5122||HK|Hybrid|RTRR||... at 83%

>Cluster 63

0 977aa, >LILAB_18015||RR|VieB|R|Orphan|... at 61%

1 927aa, >MXAN_2050||RR|VieB|R|Orphan|... at 64%

2 935aa, >COCOR_06041||RR|CheY|R|Orphan|... at 54%

3 929aa, >STAUR_6212||RR|VieB|R|Orphan|... at 53%

4 1048aa, >A176_7230||RR|VieB|R||... *

>Cluster 64

0 981aa, >LILAB_05125||HK|Hybrid|RTRRR|Complex|... at 78%

1 981aa, >MXAN_0733|RodK|HK|Hybrid|RTRRR|Complex|... at 78%

2 988aa, >COCOR_00615||HK|Hybrid|RTRRR|Complex|... *

3 968aa, >STAUR_7556||HK|Hybrid|TR|Complex|... at 69%

4 980aa, >A176_3379||HK|Hybrid|TRRR||... at 78%

>Cluster 65

0 950aa, >LILAB_07765||HK|Hybrid|TR|Complex|... at 64%

1 967aa, >MXAN_0195||HK|Hybrid|TR|Complex|... at 63%

2 948aa, >COCOR_07842||HK|Hybrid|TR|Complex|... at 55%

3 976aa, >STAUR_0267||HK|Hybrid|TR|Complex|... *

4 954aa, >A176_3115||HK|Hybrid|TR||... at 64%

>Cluster 66

0 948aa, >LILAB_20705||HK|Hybrid|TRT|Complex|... at 61%

1 970aa, >MXAN_2606||HK|Hybrid|TRT|Complex|... at 61%

2 975aa, >COCOR_05442||HK|Hybrid|TRT|Complex|... *

3 971aa, >STAUR_3336||HK|Hybrid|TRT|Complex|... at 59%

4 949aa, >A176_4788||HK|Hybrid|TRT||... at 61%

>Cluster 67^

0 126aa, >LILAB_11650A||RR|CheY|R|Complex|... at 55%

1 929aa, >LILAB_19280||HK|Unorthodox|TRRH|Complex|... at 91%

2 944aa, >MXAN_2317||HK|Unorthodox|TRRH|Complex|... *

3 909aa, >COCOR_02341||HK|Unorthodox|TRRH|Complex|... at 55%

4 915aa, >A176_2697||HK|Unorthodox|TRRH||... at 85%

>Cluster 68$

0 811aa, >LILAB_33705||HK|CheA|TR|Pair|... at 59%

1 798aa, >MXAN_5147|CheA3|HK|CheA|TR|Complex|... at 60%

2 920aa, >COCOR_02351||HK|CheA|TR|Complex|... *

3 815aa, >STAUR_5916||HK|CheA|TR|Complex|... at 55%

4 901aa, >A176_6468||HK|CheA|TR||... at 67%

>Cluster 69&

0 227aa, >LILAB_06705A||HK|Classic|T|Orphan|... at 66%

1 875aa, >MXAN_0399||HK|Classic|T|Orphan|... at 58%

2 889aa, >COCOR_07614||HK|Classic|T|Orphan|... *

3 873aa, >STAUR_8070||HK|Classic|T|Orphan|... at 51%

4 860aa, >A176_1497||HK|Classic|T||... at 57%

>Cluster 70

0 859aa, >LILAB_27605||HK|Hybrid|TRR|Complex|... at 71%

1 862aa, >MXAN_3879||HK|Hybrid|TRR|Complex|... at 71%

2 866aa, >COCOR_06549||HK|Hybrid|TRR|Complex|... *

3 853aa, >STAUR_4470||HK|Hybrid|TRR|Complex|... at 71%

4 864aa, >A176_6420||HK|Hybrid|TRR||... at 71%

>Cluster 71&

0 843aa, >LILAB_13000||HK|CheA|T|Pair|... at 85%

1 857aa, >MXAN_6692|DifE|HK|CheA|T|Pair|... *

2 789aa, >COCOR_07283||HK|CheA|T|Pair|... at 59%

3 688aa, >STAUR_1221||HK|CheA|T|Pair|... at 58%

4 849aa, >A176_2233||HK|CheA|T||... at 83%

>Cluster 72

0 849aa, >LILAB_21125||HK|CheA|TR|Complex|... *

1 848aa, >MXAN_2686|CheA4|HK|CheA|TR|Complex|... at 94%

2 845aa, >COCOR_05356||HK|CheA|TR|Complex|... at 73%

3 842aa, >STAUR_3423||HK|CheA|TR|Complex|... at 70%

4 849aa, >A176_0128||HK|CheA|TR||... at 91%

>Cluster 73

0 802aa, >LILAB_12195||HK|Hybrid|TR|Pair|... at 89%

1 842aa, >MXAN_6855|EspC|HK|Hybrid|TR|Pair|... *

2 842aa, >COCOR_07432||HK|Hybrid|TR|Pair|... at 58%

3 800aa, >STAUR_0999||HK|Hybrid|TR|Complex|... at 52%

4 809aa, >A176_3550||HK|Hybrid|TR||... at 83%

>Cluster 74

0 835aa, >LILAB_31140||HK|Hybrid|TR|Pair|... at 98%

1 836aa, >MXAN_4640|SgmT|HK|Hybrid|TR|Pair|... *

2 833aa, >COCOR_04844||HK|Hybrid|TR|Pair|... at 84%

3 827aa, >STAUR_5413||HK|Hybrid|TR|Pair|... at 73%

4 835aa, >A176_1961||HK|Hybrid|TR||... at 96%

>Cluster 75

0 830aa, >LILAB_03250||HK|Classic|T|Pair|... at 71%

1 830aa, >MXAN_1077|SpdS|HK|Classic|T|Pair|... at 71%

2 836aa, >COCOR_01017||HK|Classic|T|Pair|... *

3 836aa, >STAUR_1638||HK|Classic|T|Pair|... at 62%

4 830aa, >A176_4005||HK|Classic|T||... at 70%

>Cluster 76

0 778aa, >LILAB_28855||HK|CheA|TR|Complex|... *

1 777aa, >MXAN_4140|FrzE|HK|CheA|TR|Complex|... at 96%

2 770aa, >COCOR_03835||HK|CheA|TR|Complex|... at 85%

3 767aa, >STAUR_4587||HK|CheA|TR|Complex|... at 82%

4 775aa, >A176_1845||HK|CheA|TR||... at 95%

>Cluster 77

0 768aa, >LILAB_04045||HK|Hybrid|TR|Pair|... at 97%

1 777aa, >MXAN_0931|EspA|HK|Hybrid|TR|Pair|... *

2 755aa, >COCOR_00882||HK|Hybrid|TR|Pair|... at 82%

3 710aa, >STAUR_7060||HK|Hybrid|TR|Pair|... at 72%

4 754aa, >A176_0939||HK|Hybrid|TR||... at 94%

>Cluster 78

0 714aa, >LILAB_16155||HK|CheA|TR|Complex|... at 63%

1 714aa, >MXAN_6029||HK|CheA|TR|Complex|... at 61%

2 725aa, >COCOR_06563||HK|CheA|TR|Complex|... at 65%

3 763aa, >STAUR_6706||HK|CheA|TR|Complex|... *

4 564aa, >A176_1114||HK|Hybrid|TR||... at 76%

>Cluster 79

0 748aa, >LILAB_24490||HK|Classic|T|Orphan|... at 50%

1 747aa, >A2cp1_2057||HK|Classic|T|Pair|... at 84%

2 747aa, >Adeh_1907||HK|Classic|T|Pair|... at 84%

3 747aa, >AnaeK_1972||HK|Classic|T|Pair|... at 84%

4 752aa, >Anae109_1952||HK|Classic|T|Pair|... *

>Cluster 80

0 724aa, >LILAB_11650||HK|CheA|TR|Complex|... at 89%

1 726aa, >MXAN_6951||HK|CheA|TR|Complex|... at 90%

2 720aa, >COCOR_07521||HK|CheA|TR|Complex|... at 73%

3 710aa, >STAUR_0871||HK|CheA|TR|Complex|... at 57%

4 738aa, >A176_5466||HK|CheA|TR||... *

>Cluster 81

0 684aa, >LILAB_14795||HK|Hybrid|TR|Pair|... at 93%

1 719aa, >MXAN_6335||HK|Hybrid|TR|Pair|... *

2 677aa, >COCOR_06953||HK|Hybrid|TR|Pair|... at 74%

3 682aa, >STAUR_6916||HK|Hybrid|TR|Orphan|... at 68%

4 696aa, >A176_2395||HK|Hybrid|TR||... at 91%

>Cluster 82&

0 701aa, >LILAB_00990||HK|Classic|T|Pair|... at 91%

1 706aa, >MXAN_1553||HK|Classic|T|Pair|... *

2 705aa, >COCOR_01561||HK|Classic|T|Pair|... at 69%

3 561aa, >STAUR_2326||HK|Classic|T|Pair|... at 68%

4 701aa, >A176_3849||HK|Classic|T||... at 85%

>Cluster 83

0 650aa, >LILAB_07085||HK|Hybrid|TR|Pair|... at 81%

1 691aa, >MXAN_0314||HK|Hybrid|TR|Pair|... at 80%

2 661aa, >COCOR_07725||HK|Hybrid|TR|Pair|... at 67%

3 675aa, >STAUR_7822||HK|Hybrid|TR|Pair|... at 52%

4 703aa, >A176_6272||HK|Hybrid|TR||... *

>Cluster 84&

0 682aa, >LILAB_07550||HK|Hybrid|TRR|Complex|... *

1 513aa, >MXAN_0230||HK|Hybrid|TRR|Complex|... at 64%

2 682aa, >COCOR_07813||HK|Hybrid|TRR|Complex|... at 78%

3 682aa, >STAUR_0289||HK|Hybrid|TR|Complex|... at 75%

4 682aa, >A176_5373||HK|Hybrid|TRR||... at 93%

>Cluster 85$

0 655aa, >LILAB_11575||HK|Hybrid|RT|Complex|... at 86%

1 656aa, >MXAN_6966||HK|Hybrid|RRT|Complex|... at 84%

2 657aa, >COCOR_07558||HK|Hybrid|RT|Complex|... at 66%

3 647aa, >STAUR_0856||HK|Hybrid|RT|Pair|... at 59%

4 680aa, >A176_5484||HK|Hybrid|RRT||... *

>Cluster 86

0 647aa, >LILAB_12145||PP|HisKa|H|Complex|... at 96%

1 672aa, >MXAN_6866||PP|HisKa|H|Complex|... *

2 652aa, >COCOR_07443||HK|Classic|T|Complex|... at 79%

3 631aa, >STAUR_0990||HK|Classic|T|Complex|... at 73%

4 659aa, >A176_3536||HK|Classic|T||... at 92%

>Cluster 87

0 660aa, >LILAB_12795||HK|Hybrid|RT|Complex|... at 96%

1 671aa, >MXAN_6734||HK|Hybrid|RT|Complex|... *

2 657aa, >COCOR_07310||HK|Hybrid|RT|Complex|... at 81%

3 661aa, >STAUR_1170||HK|Hybrid|RT|Complex|... at 76%

4 636aa, >A176_0389||HK|Hybrid|RT||... at 96%

>Cluster 88

0 632aa, >LILAB_12150||HK|Classic|T|Complex|... at 79%

1 631aa, >MXAN_6865||HK|Classic|T|Complex|... at 78%

2 640aa, >COCOR_07442||HK|Classic|T|Complex|... *

3 639aa, >STAUR_0991||HK|Classic|T|Complex|... at 68%

4 631aa, >A176_3537||HK|Classic|T||... at 78%

>Cluster 89&

0 565aa, >LILAB_07760||HK|Classic|T|Complex|... at 93%

1 635aa, >MXAN_0196||HK|Classic|T|Complex|... *

2 584aa, >COCOR_07841||HK|Classic|T|Complex|... at 56%

3 446aa, >STAUR_0269||HK|Classic|T|Complex|... at 51%

4 574aa, >A176_3113||HK|Classic|T||... at 85%

>Cluster 90

0 612aa, >LILAB_14880||HK|Hybrid|TR|Pair|... at 75%

1 632aa, >MXAN_6315||HK|Hybrid|TR|Pair|... at 74%

2 634aa, >COCOR_06931||HK|Hybrid|TR|Pair|... *

3 631aa, >STAUR_6883||HK|Hybrid|TR|Orphan|... at 70%

4 631aa, >A176_2380||HK|Hybrid|TR||... at 75%

>Cluster 91

0 616aa, >LILAB_33145||RR|PleD|R|Complex|... *

1 616aa, >MXAN_5053||RR|PleD|R|Complex|... at 97%

2 616aa, >COCOR_02426||RR|PleD|R|Complex|... at 86%

3 616aa, >STAUR_5835||RR|PleD|R|Complex|... at 81%

4 616aa, >A176_6569||RR|PleD|R||... at 96%

>Cluster 92

0 615aa, >LILAB_30355||HK|Classic|T|Complex|... *

1 615aa, >MXAN_4444||HK|Classic|T|Complex|... at 93%

2 612aa, >COCOR_03339||HK|Classic|T|Complex|... at 68%

3 612aa, >STAUR_4800||HK|Classic|T|Complex|... at 69%

4 615aa, >A176_5121||HK|Classic|T||... at 86%

>Cluster 93

0 541aa, >LILAB_11635||HK|Classic|T|Complex|... at 93%

1 588aa, >MXAN_6953|SocD|HK|Classic|T|Complex|... *

2 576aa, >COCOR_07523||HK|Classic|T|Complex|... at 73%

3 558aa, >STAUR_0869||HK|Classic|T|Complex|... at 67%

4 520aa, >A176_5469||HK|Classic|T||... at 90%

>Cluster 94$

0 549aa, >LILAB_31870||HK|Classic|T|Complex|... at 65%

1 549aa, >MXAN_4786||HK|Classic|T|Complex|... at 64%

2 555aa, >COCOR_02120||HK|Classic|T|Pair|... at 59%

3 584aa, >STAUR_6002||HK|Classic|T|Pair|... *

4 515aa, >A176_5974||HK|Classic|T||... at 66%

>Cluster 95

0 581aa, >LILAB_26850||RR|unclassified|R|Complex|... *

1 577aa, >MXAN_3734||RR|unclassified|R|Complex|... at 92%

2 578aa, >COCOR_04268||RR|unclassified|R|Complex|... at 78%

3 565aa, >STAUR_4234||RR|unclassified|R|Pair|... at 74%

4 565aa, >A176_6152||RR|unclassified|R||... at 90%

>Cluster 96

0 566aa, >LILAB_24235||HK|Hybrid|TR|Pair|... at 82%

1 530aa, >MXAN_3290|KfaA|HK|Hybrid|TR|Pair|... at 83%

2 581aa, >COCOR_03034||HK|Hybrid|TR|Pair|... *

3 518aa, >STAUR_3728||HK|Hybrid|TR|Pair|... at 78%

4 568aa, >A176_2882||HK|Hybrid|TR||... at 82%

>Cluster 97

0 549aa, >LILAB_33925||HK|Classic|T|Orphan|... at 95%

1 578aa, >MXAN_5184|CrdS|HK|Classic|T|Orphan|... *

2 549aa, >COCOR_02322||HK|Classic|T|Orphan|... at 83%

3 549aa, >STAUR_5936||HK|Classic|T|Orphan|... at 80%

4 549aa, >A176_7336||HK|Classic|T||... at 92%

>Cluster 98

0 522aa, >LILAB_36415||HK|Classic|T|Pair|... at 55%

1 525aa, >MXAN_5785|PilS|HK|Classic|T|Pair|... at 54%

2 522aa, >COCOR_06285||HK|Classic|T|Pair|... at 56%

3 530aa, >STAUR_6455||HK|Classic|T|Pair|... at 55%

4 566aa, >A176_4464||HK|Classic|T||... *

>Cluster 99

0 553aa, >LILAB_23850||RR|NtrC|R|Complex|... at 83%

1 553aa, >MXAN_3214|ActB|RR|NtrC|R|Complex|... at 82%

2 561aa, >COCOR_02963||RR|NtrC|R|Complex|... *

3 547aa, >STAUR_3648||RR|NtrC|R|Orphan|... at 82%

4 554aa, >A176_6381||RR|NtrC|R||... at 82%

>Cluster 100

0 533aa, >LILAB_34655||HK|Hybrid|RT|Complex|... *

1 527aa, >MXAN_5365|HsfB|HK|Hybrid|RT|Complex|... at 96%

2 526aa, >COCOR_02205||HK|Hybrid|RT|Complex|... at 84%

3 522aa, >STAUR_6072||HK|Hybrid|RT|Complex|... at 79%

4 527aa, >A176_7544||HK|Hybrid|RT||... at 96%

>Cluster 101&

0 390aa, >LILAB_23210||HK|Classic|T|Orphan|... at 59%

1 390aa, >MXAN_3098||HK|Classic|T|Orphan|... at 60%

2 533aa, >COCOR_06374||HK|Classic|T|Orphan|... *

3 395aa, >STAUR_7263||HK|Classic|T|Orphan|... at 58%

4 390aa, >A176_6939||HK|Classic|T||... at 58%

>Cluster 102

0 463aa, >LILAB_30460||HK|Hybrid|TRR|Complex|... at 94%

1 517aa, >MXAN_4465||HK|Hybrid|TRR|Complex|... *

2 509aa, >COCOR_03314||HK|Hybrid|TR|Complex|... at 75%

3 517aa, >STAUR_4820||HK|Hybrid|TR|Complex|... at 68%

4 505aa, >A176_5141||HK|Hybrid|TR||... at 90%

>Cluster 103

0 515aa, >LILAB_33055||HK|Classic|T|Orphan|... *

1 515aa, >MXAN_5034||HK|Classic|T|Orphan|... at 94%

2 513aa, >COCOR_02435||HK|Classic|T|Orphan|... at 79%

3 513aa, >STAUR_5829||HK|Classic|T|Orphan|... at 74%

4 513aa, >A176_6591||HK|Classic|T||... at 92%

>Cluster 104

0 510aa, >LILAB_02970||HK|Classic|T|Pair|... at 66%

1 510aa, >MXAN_1129|FrgB|HK|Classic|T|Pair|... at 66%

2 510aa, >COCOR_01086||HK|Classic|T|Pair|... at 68%

3 513aa, >STAUR_1707||HK|Classic|T|Pair|... *

4 510aa, >A176_0464||HK|Classic|T||... at 64%

>Cluster 105

0 509aa, >LILAB_02050||HK|Classic|T|Pair|... at 74%

1 509aa, >MXAN_1350||HK|Classic|T|Pair|... at 73%

2 510aa, >COCOR_01239||HK|Classic|T|Pair|... *

3 506aa, >STAUR_2103||HK|Classic|T|Pair|... at 68%

4 509aa, >A176_0644||HK|Classic|T||... at 73%

>Cluster 106

0 496aa, >LILAB_33735||RR|NtrC|R|Orphan|... at 83%

1 498aa, >MXAN_5153|CrdA|RR|NtrC|R|Orphan|... at 82%

2 486aa, >COCOR_02345||RR|NtrC|R|Orphan|... at 85%

3 501aa, >STAUR_5922||RR|NtrC|R|Orphan|... *

4 494aa, >A176_6462||RR|NtrC|R||... at 82%

>Cluster 107

0 499aa, >LILAB_33140||RR|unclassified|R|Complex|... *

1 499aa, >MXAN_5052||RR|unclassified|R|Complex|... at 97%

2 499aa, >COCOR_02427||RR|CheY|R|Complex|... at 79%

3 499aa, >STAUR_5834||RR|CheY|R|Complex|... at 76%

4 499aa, >A176_6570||RR|CheY|R||... at 96%

>Cluster 108

0 497aa, >LILAB_02480||HK|Classic|T|Orphan|... *

1 497aa, >MXAN_1249|SasS|HK|Classic|T|Orphan|... at 92%

2 493aa, >COCOR_01160||HK|Classic|T|Orphan|... at 73%

3 488aa, >STAUR_1875||HK|Classic|T|Orphan|... at 72%

4 494aa, >A176_0560||HK|Classic|T||... at 88%

>Cluster 109$

0 492aa, >LILAB_04010||HK|Classic|T|Pair|... *

1 492aa, >MXAN_0938||HK|Classic|T|Pair|... at 96%

2 492aa, >COCOR_07530||HK|Classic|T|Complex|... at 75%

3 484aa, >STAUR_4536||HK|Classic|T|Complex|... at 67%

4 492aa, >A176_0947||HK|Classic|T||... at 92%

>Cluster 110

0 487aa, >LILAB_33590||RR|NtrC|R|Pair|... at 98%

1 488aa, >MXAN_5124|MrpB|RR|NtrC|R|Pair|... *

2 488aa, >COCOR_02367||RR|NtrC|R|Pair|... at 90%

3 480aa, >STAUR_5890||RR|NtrC|R|Pair|... at 85%

4 486aa, >A176_6492||RR|NtrC|R||... at 97%

>Cluster 111

0 476aa, >LILAB_02770||HK|Classic|T|Pair|... *

1 476aa, >MXAN_1166||HK|Classic|T|Pair|... at 95%

2 473aa, >COCOR_01122||HK|Classic|T|Pair|... at 74%

3 469aa, >STAUR_1782||HK|Classic|T|Pair|... at 68%

4 476aa, >A176_0507||HK|Classic|T||... at 93%

>Cluster 112

0 466aa, >LILAB_34405||HK|Classic|T|Pair|... at 64%

1 466aa, >MXAN_5314||HK|Classic|T|Pair|... at 64%

2 471aa, >COCOR_02254||HK|Classic|T|Pair|... *

3 464aa, >STAUR_6025||HK|Classic|T|Pair|... at 62%

4 430aa, >A176_7493||HK|Classic|T||... at 68%

>Cluster 113

0 468aa, >LILAB_02975||RR|NtrC|R|Pair|... *

1 466aa, >MXAN_1128|FrgC|RR|NtrC|R|Pair|... at 93%

2 463aa, >COCOR_01085||RR|NtrC|R|Pair|... at 75%

3 464aa, >STAUR_1706||RR|NtrC|R|Pair|... at 75%

4 461aa, >A176_0463||RR|NtrC|R||... at 93%

>Cluster 114

0 464aa, >A2cp1_1954||RR|NtrC|R|Pair|... at 86%

1 463aa, >Adeh_2009||RR|NtrC|R|Pair|... at 85%

2 464aa, >AnaeK_1869||RR|NtrC|R|Pair|... at 86%

3 466aa, >Anae109_1798||RR|NtrC|R|Pair|... *

4 363aa, >PPSIR1_16240||RR|unclassified|R||... at 50%

>Cluster 115

0 450aa, >LILAB_29110||RR|NtrC|R|Pair|... at 63%

1 450aa, >MXAN_4196||RR|NtrC|R|Pair|... at 63%

2 461aa, >PPSIR1_07543||RR|NtrC|R||... *

3 447aa, >Hoch_3222||RR|NtrC|R|Pair|... at 63%

4 450aa, >A176_4058||RR|NtrC|R||... at 64%

>Cluster 116$

0 458aa, >LILAB_32810||RR|NtrC|R|Complex|... at 55%

1 458aa, >MXAN_4977||RR|NtrC|R|Complex|... at 56%

2 451aa, >STAUR_8088||RR|NtrC|R|Pair|... at 63%

3 458aa, >A176_3027||RR|NtrC|R||... at 55%

4 461aa, >sce7862||RR|NtrC|R|Orphan|... *

>Cluster 117$

0 453aa, >LILAB_31840||HK|Classic|T|Pair|... at 88%

1 454aa, >MXAN_4778|PhoR1|HK|Classic|T|Pair|... at 86%

2 453aa, >COCOR_02619||HK|Classic|T|Pair|... at 72%

3 452aa, >STAUR_5626||HK|Classic|T|Complex|... at 71%

4 455aa, >A176_5982||HK|Classic|T||... *

>Cluster 118

0 448aa, >LILAB_02765||RR|NtrC|R|Pair|... at 87%

1 447aa, >MXAN_1167|Nla28|RR|NtrC|R|Pair|... at 87%

2 453aa, >COCOR_01123||RR|NtrC|R|Pair|... *

3 446aa, >STAUR_1783||RR|NtrC|R|Pair|... at 85%

4 447aa, >A176_0508||RR|NtrC|R||... at 86%

>Cluster 119

0 452aa, >LILAB_07955||HK|Classic|T|Orphan|... *

1 452aa, >MXAN_0153||HK|Classic|T|Orphan|... at 92%

2 451aa, >COCOR_07906||HK|Classic|T|Orphan|... at 55%

3 448aa, >STAUR_0208||HK|Classic|T|Orphan|... at 55%

4 442aa, >A176_3162||HK|Classic|T||... at 86%

>Cluster 120

0 427aa, >LILAB_28525||HK|Classic|T|Pair|... at 84%

1 427aa, >MXAN_4071||HK|Classic|T|Pair|... at 83%

2 427aa, >COCOR_03879||HK|Classic|T|Pair|... at 80%

3 428aa, >STAUR_4526||HK|Classic|T|Pair|... *

4 427aa, >A176_1784||HK|Classic|T||... at 84%

>Cluster 121

0 387aa, >LILAB_11265||RR|unclassified|R|Orphan|... at 57%

1 387aa, >MXAN_7024||RR|unclassified|R|Orphan|... at 55%

2 375aa, >COCOR_00381||RR|unclassified|R|Orphan|... at 58%

3 388aa, >STAUR_0801||RR|unclassified|RR|Complex|... *

4 386aa, >A176_5554||RR|unclassified|R||... at 56%

>Cluster 122

0 385aa, >LILAB_21050||HK|Hybrid|RT|Complex|... *

1 385aa, >MXAN_2670|AsgA|HK|Hybrid|RT|Complex|... at 96%

2 385aa, >COCOR_05373||HK|Hybrid|RT|Complex|... at 86%

3 385aa, >STAUR_3406||HK|Hybrid|RT|Complex|... at 79%

4 385aa, >A176_0113||HK|Hybrid|RT||... at 96%

>Cluster 123

0 373aa, >LILAB_36380||HK|Classic|T|Pair|... *

1 373aa, >MXAN_5778||HK|Classic|T|Pair|... at 89%

2 372aa, >COCOR_06278||HK|Classic|T|Pair|... at 70%

3 372aa, >STAUR_6447||HK|Classic|T|Pair|... at 68%

4 373aa, >A176_4471||HK|Classic|T||... at 76%

>Cluster 124

0 355aa, >LILAB_16160||RR|CheB|R|Complex|... at 90%

1 355aa, >MXAN_6028||RR|CheB|R|Complex|... at 91%

2 355aa, >COCOR_06562||RR|CheB|R|Complex|... at 83%

3 351aa, >STAUR_6705||RR|CheB|R|Complex|... at 71%

4 357aa, >A176_1113||RR|CheB|R||... *

>Cluster 125$

0 352aa, >LILAB_33685||RR|CheB|R|Orphan|... *

1 352aa, >MXAN_5145|CheB|RR|CheB|R|Complex|... at 98%

2 352aa, >COCOR_02352||RR|CheB|R|Complex|... at 80%

3 350aa, >STAUR_5915||RR|CheB|R|Complex|... at 78%

4 352aa, >A176_6470||RR|CheB|R||... at 97%

>Cluster 126

0 341aa, >LILAB_22535||HK|Classic|T|Pair|... at 64%

1 341aa, >MXAN_2961||HK|Classic|T|Pair|... at 63%

2 352aa, >COCOR_05101||HK|Classic|T|Pair|... *

3 338aa, >STAUR_5150||HK|Classic|T|Pair|... at 58%

4 338aa, >A176_4868||HK|Classic|T||... at 63%

>Cluster 127

0 345aa, >LILAB_31710||RR|CheB|R|Complex|... at 71%

1 345aa, >MXAN_4752||RR|CheB|R|Complex|... at 71%

2 347aa, >COCOR_02644||RR|CheB|R|Pair|... at 66%

3 349aa, >STAUR_5602||RR|CheB|R|Orphan|... *

4 345aa, >A176_6009||RR|CheB|R||... at 69%

>Cluster 128

0 347aa, >LILAB_36445||RR|PleD|R|Orphan|... at 76%

1 347aa, >MXAN_5791||RR|PleD|R|Orphan|... at 76%

2 339aa, >COCOR_06291||RR|PleD|R|Orphan|... at 78%

3 348aa, >STAUR_6461||RR|PleD|R|Orphan|... *

4 347aa, >A176_4458||RR|PleD|R||... at 76%

>Cluster 129

0 315aa, >LILAB_33585||HK|Classic|T|Pair|... at 69%

1 341aa, >MXAN_5123|MrpA|HK|Classic|T|Pair|... at 67%

2 328aa, >COCOR_02368||HK|Classic|T|Pair|... at 70%

3 346aa, >STAUR_5889||HK|Classic|T|Pair|... *

4 330aa, >A176_6493||HK|Classic|T||... at 69%

>Cluster 130

0 332aa, >LILAB_30295||HK|Hybrid|RT|Pair|... at 60%

1 331aa, >MXAN_4432||HK|Hybrid|RT|Pair|... at 60%

2 335aa, >COCOR_03464||HK|Hybrid|RT|Pair|... at 60%

3 340aa, >STAUR_4783||HK|Hybrid|RT|Pair|... *

4 331aa, >A176_5108||HK|Hybrid|RT||... at 59%

>Cluster 131

0 329aa, >LILAB_21060||RR|unclassified|R|Complex|... *

1 329aa, >MXAN_2671||RR|unclassified|R|Complex|... at 93%

2 322aa, >COCOR_05371||RR|CheY|R|Complex|... at 76%

3 323aa, >STAUR_3408||RR|CheY|R|Complex|... at 70%

4 329aa, >A176_0115||RR|CheY|R||... at 89%

>Cluster 132

0 318aa, >LILAB_26855||RR|PleD|R|Complex|... at 80%

1 318aa, >MXAN_3735||RR|PleD|R|Complex|... at 81%

2 319aa, >COCOR_04267||RR|PleD|R|Complex|... at 77%

3 322aa, >STAUR_4235||RR|PleD|R|Pair|... *

4 318aa, >A176_6151||RR|PleD|R||... at 82%

>Cluster 133

0 292aa, >LILAB_28875||RR|FrzZ|RR|Complex|... at 84%

1 290aa, >MXAN_4144|FrzZ|RR|FrzZ|RR|Complex|... at 85%

2 289aa, >COCOR_03831||RR|FrzZ|RR|Complex|... at 80%

3 293aa, >STAUR_4591||RR|FrzZ|RR|Complex|... *

4 291aa, >A176_1849||RR|FrzZ|RR||... at 83%

>Cluster 134

0 288aa, >LILAB_16140||RR|CheV|R|Complex|... *

1 288aa, >MXAN_6032||RR|CheV|R|Complex|... at 95%

2 288aa, >COCOR_06566||RR|CheV|R|Complex|... at 78%

3 287aa, >STAUR_6709||RR|CheV|R|Complex|... at 74%

4 288aa, >A176_1117||RR|CheV|R||... at 90%

>Cluster 135

0 243aa, >LILAB_28530||RR|NarL|R|Pair|... at 85%

1 246aa, >MXAN_4072||RR|NarL|R|Pair|... at 84%

2 249aa, >COCOR_03878||RR|NarL|R|Pair|... at 84%

3 254aa, >STAUR_4527||RR|NarL|R|Pair|... *

4 240aa, >A176_1785||RR|NarL|R||... at 86%

>Cluster 136&

0 249aa, >LILAB_03200||RR|unclassified|R|Orphan|... at 80%

1 249aa, >MXAN_1087||RR|unclassified|R|Orphan|... at 79%

2 250aa, >COCOR_01026||RR|unclassified|R|Orphan|... *

3 180aa, >STAUR_1647||RR|CheY|R|Orphan|... at 70%

4 230aa, >A176_4015||RR|unclassified|R||... at 81%

>Cluster 137

0 206aa, >LILAB_28330||PP|HisKa|H|Complex|... at 91%

1 245aa, >MXAN_4043||PP|HisKa|H|Pair|... *

2 220aa, >COCOR_03898||PP|HisKa|H|Pair|... at 66%

3 206aa, >STAUR_4507||PP|HisKa|H|Pair|... at 68%

4 213aa, >A176_1745||PP|HisKa|H||... at 86%

>Cluster 138

0 198aa, >LILAB_23310||RR|NarL|R|Orphan|... at 86%

1 229aa, >MXAN_3117|FruA|RR|NarL|R|Orphan|... at 84%

2 193aa, >COCOR_04934||RR|NarL|R|Orphan|... at 86%

3 236aa, >STAUR_4943||RR|NarL|R|Orphan|... *

4 229aa, >A176_6957||RR|NarL|R||... at 84%

>Cluster 139

0 230aa, >LILAB_28975||RR|OmpR|R|Pair|... at 51%

1 232aa, >MXAN_4164||RR|OmpR|R|Pair|... at 53%

2 227aa, >Hoch_0268||RR|OmpR|R|Pair|... at 54%

3 232aa, >A176_4023||RR|OmpR|R||... at 52%

4 234aa, >sce4878||RR|OmpR|R|Pair|... *

>Cluster 140$&

0 220aa, >LILAB_05150||RR|NarL|R|Complex|... at 62%

1 163aa, >MXAN_0727||RR|CheY|R|Complex|... at 52%

2 229aa, >COCOR_00609||RR|NarL|R|Pair|... *

3 223aa, >STAUR_7561||RR|NarL|R|Orphan|... at 52%

4 228aa, >A176_3384||RR|NarL|R||... at 60%

>Cluster 141

0 221aa, >LILAB_26725||RR|NarL|R|Orphan|... at 97%

1 223aa, >MXAN_3711||RR|NarL|R|Orphan|... *

2 219aa, >COCOR_04294||RR|NarL|R|Orphan|... at 86%

3 218aa, >STAUR_4210||RR|NarL|R|Orphan|... at 86%

4 221aa, >A176_6177||RR|NarL|R||... at 94%

>Cluster 142

0 193aa, >LILAB_03170||RR|MerR|R|Orphan|... at 89%

1 201aa, >MXAN_1093||RR|MerR|R|Orphan|... at 85%

2 196aa, >COCOR_01032||RR|MerR|R|Orphan|... at 85%

3 209aa, >STAUR_1652||RR|MerR|R|Orphan|... *

4 206aa, >A176_0426||RR|MerR|R||... at 83%

>Cluster 143

0 159aa, >LILAB_16060||RR|CheY|R|Orphan|... at 76%

1 158aa, >MXAN_6046||RR|CheY|R|Orphan|... at 77%

2 160aa, >COCOR_06580||RR|CheY|R|Orphan|... *

3 159aa, >STAUR_6741||RR|CheY|R|Orphan|... at 57%

4 158aa, >A176_1131||RR|CheY|R||... at 77%

>Cluster 144

0 153aa, >LILAB_17870||RR|CheY|R|Orphan|... at 96%

1 153aa, >MXAN_2021||RR|CheY|R|Orphan|... at 96%

2 149aa, >COCOR_02042||RR|CheY|R|Orphan|... at 69%

3 151aa, >STAUR_2840||RR|CheY|R|Orphan|... at 75%

4 154aa, >A176_2851||RR|CheY|R||... *

>Cluster 145

0 137aa, >LILAB_16845||RR|CheY|R|Pair|... at 95%

1 153aa, >MXAN_5889||RR|CheY|R|Pair|... *

2 132aa, >COCOR_06414||RR|CheY|R|Orphan|... at 71%

3 134aa, >STAUR_6555||RR|CheY|R|Orphan|... at 57%

4 137aa, >A176_6745||RR|CheY|R||... at 83%

>Cluster 146

0 152aa, >LILAB_04820||RR|CheY|R|Complex|... *

1 149aa, >STAUR_7450||RR|CheY|R|Complex|... at 87%

2 145aa, >Hoch_4644||RR|CheY|R|Pair|... at 68%

3 147aa, >Hoch_4791||RR|CheY|R|Orphan|... at 56%

4 152aa, >A176_4333||RR|CheY|R||... at 92%

>Cluster 147

0 126aa, >LILAB_31915||RR|CheY|R|Orphan|... at 95%

1 148aa, >MXAN_4794||RR|CheY|R|Orphan|... *

2 127aa, >COCOR_02603||RR|CheY|R|Orphan|... at 84%

3 143aa, >STAUR_5635||RR|CheY|R|Orphan|... at 66%

4 126aa, >A176_5966||RR|CheY|R||... at 92%

>Cluster 148

0 133aa, >LILAB_14975||RR|CheY|R|Orphan|... at 64%

1 134aa, >MXAN_6296||RR|CheY|R|Orphan|... at 64%

2 132aa, >COCOR_06910||RR|CheY|R|Orphan|... at 64%

3 146aa, >STAUR_6855||RR|CheY|R|Orphan|... *

4 128aa, >A176_2357||RR|CheY|R||... at 67%

>Cluster 149

0 120aa, >LILAB_33940||RR|CheY|R|Orphan|... at 95%

1 142aa, >MXAN_5189||RR|CheY|R|Orphan|... *

2 120aa, >COCOR_02319||RR|CheY|R|Orphan|... at 79%

3 120aa, >STAUR_5939||RR|CheY|R|Orphan|... at 78%

4 119aa, >A176_7341||RR|CheY|R||... at 90%

>Cluster 150

0 134aa, >LILAB_09205||RR|CheY|R|Orphan|... at 55%

1 134aa, >MXAN_7420||RR|CheY|R|Orphan|... at 53%

2 135aa, >COCOR_08036||RR|CheY|R|Orphan|... at 53%

3 141aa, >STAUR_7883||RR|CheY|R|Orphan|... *

4 134aa, >A176_3754||RR|CheY|R||... at 55%

>Cluster 151

0 135aa, >LILAB_35755||RR|CheY|R|Orphan|... at 77%

1 133aa, >MXAN_5656||RR|CheY|R|Orphan|... at 80%

2 133aa, >COCOR_06147||RR|CheY|R|Orphan|... at 79%

3 137aa, >STAUR_6336||RR|CheY|R|Orphan|... *

4 135aa, >A176_7141||RR|CheY|R||... at 77%

>Cluster 152

0 135aa, >LILAB_15770||RR|CheY|R|Orphan|... *

1 135aa, >MXAN_6099||RR|CheY|R|Orphan|... at 81%

2 135aa, >COCOR_06626||RR|CheY|R|Orphan|... at 74%

3 135aa, >STAUR_6795||RR|CheY|R|Orphan|... at 72%

4 135aa, >A176_3436||RR|CheY|R||... at 86%

>Cluster 153$

0 126aa, >LILAB_11565||RR|CheY|R|Complex|... at 50%

1 126aa, >MXAN_6968||RR|CheY|R|Orphan|... at 51%

2 125aa, >COCOR_07560||RR|CheY|R|Complex|... at 54%

3 135aa, >STAUR_6567||RR|CheY|R|Pair|... *

4 126aa, >A176_5486||RR|CheY|R||... at 50%

>Cluster 154

0 131aa, >A2cp1_2816||RR|CheY|R|Pair|... *

1 131aa, >Adeh_2635||RR|CheY|R|Pair|... at 96%

2 131aa, >AnaeK_2721||RR|CheY|R|Pair|... at 99%

3 126aa, >Anae109_2598||RR|CheY|R|Pair|... at 76%

4 122aa, >sce0429||RR|CheY|R|Pair|... at 52%

>Cluster 155

0 122aa, >LILAB_11390||RR|CheY|R|Complex|... at 56%

1 122aa, >MXAN_7001||RR|CheY|R|Complex|... at 57%

2 128aa, >COCOR_00396||RR|CheY|R|Complex|... at 63%

3 130aa, >STAUR_0840||RR|CheY|R|Complex|... *

4 122aa, >A176_5527||RR|CheY|R||... at 57%

>Cluster 156

0 128aa, >LILAB_16135||RR|CheY|R|Complex|... *

1 128aa, >MXAN_6033||RR|CheY|R|Complex|... at 100%

2 125aa, >COCOR_06567||RR|CheY|R|Complex|... at 80%

3 127aa, >STAUR_6710||RR|CheY|R|Complex|... at 74%

4 128aa, >A176_1118||RR|CheY|R||... at 98%

>Cluster 157$

0 104aa, >LILAB_30475||RR|CheY|R|Orphan|... at 99%

1 128aa, >MXAN_4468||RR|CheY|R|Orphan|... *

2 118aa, >COCOR_03312||RR|CheY|R|Complex|... at 76%

3 118aa, >STAUR_4822||RR|CheY|R|Complex|... at 72%

4 118aa, >A176_5144||RR|CheY|R||... at 99%

>Cluster 158

0 125aa, >LILAB_01905||RR|CheY|R|Orphan|... at 86%

1 125aa, >MXAN_1378||RR|CheY|R|Orphan|... at 86%

2 126aa, >COCOR_01265||RR|CheY|R|Orphan|... *

3 125aa, >STAUR_2140||RR|CheY|R|Orphan|... at 85%

4 125aa, >A176_0674||RR|CheY|R||... at 86%

>Cluster 159$

0 124aa, >LILAB_16240||RR|CheY|R|Orphan|... *

1 124aa, >MXAN_6012||RR|CheY|R|Orphan|... at 100%

2 124aa, >COCOR_06543||RR|CheY|R|Pair|... at 88%

3 124aa, >STAUR_6691||RR|CheY|R|Pair|... at 86%

4 124aa, >A176_1096||RR|CheY|R||... at 99%

>Cluster 160

0 124aa, >LILAB_31745||RR|CheY|R|Pair|... *

1 124aa, >MXAN_4759||RR|CheY|R|Pair|... at 99%

2 124aa, >COCOR_02637||RR|CheY|R|Pair|... at 80%

3 124aa, >STAUR_5608||RR|CheY|R|Pair|... at 78%

4 120aa, >A176_6002||RR|CheY|R||... at 95%

>Cluster 161

0 122aa, >LILAB_10650||RR|CheY|R|Orphan|... *

1 122aa, >MXAN_7150||RR|CheY|R|Orphan|... at 92%

2 122aa, >COCOR_00253||RR|CheY|R|Orphan|... at 72%

3 122aa, >STAUR_0630||RR|CheY|R|Orphan|... at 68%

4 122aa, >A176_5687||RR|CheY|R||... at 93%

>Cluster 162

0 121aa, >LILAB_13410||RR|CheY|R|Orphan|... at 68%

1 121aa, >MXAN_6620||RR|CheY|R|Orphan|... at 67%

2 122aa, >COCOR_03965||RR|CheY|R|Orphan|... *

3 121aa, >STAUR_0512||RR|CheY|R|Orphan|... at 69%

4 121aa, >A176_2162||RR|CheY|R||... at 69%

>Cluster 163&^

0 1020aa, >COCOR_01454||HK|Hybrid|TRT|Complex|... at 75%

1 992aa, >COCOR_02086||HK|Hybrid|TRT|Complex|... at 50%

2 2028aa, >STAUR_6958||HK|Hybrid|TRTTRT|Complex|... *

3 989aa, >STAUR_8144||HK|Hybrid|TRT|Complex|... at 51%

>Cluster 164&

0 1629aa, >LILAB_22700||RR|unclassified|R|Orphan|... *

1 1395aa, >MXAN_2991|AglZ|RR|unclassified|R|Orphan|... at 62%

2 1531aa, >COCOR_05067||RR|unclassified|R|Orphan|... at 52%

3 1521aa, >A176_5834||RR|CheY|R||... at 58%

>Cluster 165

0 1527aa, >A2cp1_0447||HK|Classic|T|Complex|... *

1 1527aa, >Adeh_0418||HK|Classic|T|Complex|... at 94%

2 1527aa, >AnaeK_0446||HK|Classic|T|Complex|... at 98%

3 1519aa, >Anae109_4151||HK|Classic|T|Complex|... at 61%

>Cluster 166

0 1359aa, >A2cp1_2891||RR|unclassified|R|Orphan|... at 58%

1 1363aa, >Adeh_2705||RR|unclassified|R|Orphan|... at 57%

2 1359aa, >AnaeK_2798||RR|unclassified|R|Orphan|... at 58%

3 1370aa, >Anae109_2699||RR|unclassified|R|Orphan|... *

>Cluster 167

0 1148aa, >COCOR_05431||HK|Hybrid|TRRR|Complex|... at 68%

1 1171aa, >STAUR_3019||HK|Hybrid|TRRR|Complex|... *

2 231aa, >Hoch_6240A||RR|FrzZ|RR|Complex|... at 51%

3 1151aa, >A176_6694||HK|Hybrid|TRRR||... at 73%

>Cluster 168

0 1069aa, >A2cp1_2991||HK|Classic|T|Complex|... at 92%

1 1070aa, >Adeh_2805||HK|Classic|T|Pair|... *

2 1069aa, >AnaeK_2900||HK|Classic|T|Pair|... at 92%

3 1070aa, >Anae109_1498||HK|Classic|T|Pair|... at 61%

>Cluster 169&

0 827aa, >LILAB_19540||HK|Classic|T|Orphan|... at 83%

1 886aa, >MXAN_2368||HK|Classic|T|Orphan|... *

2 773aa, >COCOR_04711||HK|Classic|T|Orphan|... at 63%

3 818aa, >A176_2643||HK|Classic|T||... at 81%

>Cluster 170&

0 488aa, >LILAB_06975||HK|Classic|T|Orphan|... at 55%

1 562aa, >MXAN_0340||HK|Classic|T|Orphan|... at 50%

2 856aa, >COCOR_07656||HK|Classic|T|Orphan|... *

3 516aa, >A176_3668||HK|Classic|T||... at 54%

>Cluster 171

0 800aa, >LILAB_11385||HK|Classic|T|Complex|... at 62%

1 815aa, >MXAN_7002||HK|Classic|T|Complex|... at 61%

2 816aa, >STAUR_0839||HK|Classic|T|Complex|... *

3 799aa, >A176_5528||HK|Classic|T||... at 62%

>Cluster 172$

0 774aa, >LILAB_11430||HK|Hybrid|RT|Complex|... at 55%

1 773aa, >MXAN_6996|AsgD|HK|Hybrid|RT|Complex|... at 55%

2 807aa, >STAUR_0633||HK|Hybrid|RT|Pair|... *

3 770aa, >A176_5521||HK|Hybrid|RT||... at 56%

>Cluster 173£

0 687aa, >A2cp1_4373||HK|Hybrid|TR|Pair|... at 51%

1 686aa, >Adeh_4217||HK|Hybrid|TR|Pair|... at 52%

2 686aa, >AnaeK_4350||HK|Hybrid|TR|Pair|... at 52%

3 800aa, >Anae109_4367||HK|Hybrid|TR|Complex|... *

>Cluster 174

0 767aa, >LILAB_08270||HK|Hybrid|TR|Pair|... at 88%

1 768aa, >MXAN_0095||HK|Hybrid|TR|Pair|... *

2 758aa, >STAUR_2934||HK|Hybrid|TR|Pair|... at 55%

3 766aa, >A176_3235||HK|Hybrid|TR||... at 85%

>Cluster 175

0 763aa, >A2cp1_1867||HK|Classic|T|Orphan|... *

1 763aa, >Adeh_2090||HK|Classic|T|Orphan|... at 92%

2 763aa, >AnaeK_1788||HK|Classic|T|Orphan|... at 98%

3 763aa, >Anae109_1722||HK|Classic|T|Orphan|... at 58%

>Cluster 176

0 758aa, >LILAB_03710||HK|Classic|T|Complex|... *

1 756aa, >COCOR_00946||HK|Classic|T|Complex|... at 67%

2 728aa, >STAUR_7006||HK|Classic|T|Complex|... at 65%

3 757aa, >A176_3925||HK|Classic|T||... at 82%

>Cluster 177

0 758aa, >LILAB_08445||HK|Classic|T|Orphan|... *

1 758aa, >MXAN_0060||HK|Classic|T|Orphan|... at 89%

2 756aa, >COCOR_00060||HK|Classic|T|Orphan|... at 54%

3 758aa, >A176_3269||HK|Classic|T||... at 85%

>Cluster 178

0 753aa, >A2cp1_0638||HK|CheA|TR|Complex|... at 81%

1 750aa, >Adeh_0613||HK|CheA|TR|Complex|... at 81%

2 756aa, >AnaeK_0647||HK|CheA|TR|Complex|... at 81%

3 758aa, >Anae109_0657||HK|CheA|TR|Complex|... *

>Cluster 179

0 754aa, >LILAB_05945||HK|Classic|T|Orphan|... at 77%

1 755aa, >MXAN_0571||HK|Classic|T|Orphan|... at 76%

2 754aa, >COCOR_00504||HK|Classic|T|Orphan|... at 53%

3 757aa, >A176_1347||HK|Classic|T||... *

>Cluster 180

0 741aa, >A2cp1_3151||HK|Hybrid|TR|Pair|... at 55%

1 752aa, >Adeh_2956||HK|Hybrid|TR|Pair|... at 55%

2 738aa, >AnaeK_3043||HK|Hybrid|TR|Pair|... at 55%

3 755aa, >Anae109_1671||HK|Hybrid|TR|Pair|... *

>Cluster 181$

0 747aa, >MXAN_3343||HK|Classic|T|Orphan|... at 70%

1 747aa, >COCOR_03207||HK|Classic|T|Orphan|... at 71%

2 748aa, >STAUR_3807||HK|Classic|T|Pair|... *

3 748aa, >A176_4579||HK|Classic|T||... at 70%

>Cluster 182

0 681aa, >LILAB_11085||HK|Classic|T|Orphan|... at 82%

1 739aa, >MXAN_7059||HK|Classic|T|Orphan|... *

2 684aa, >COCOR_00346||HK|Classic|T|Orphan|... at 54%

3 676aa, >A176_5592||HK|Classic|T||... at 78%

>Cluster 183

0 713aa, >A2cp1_3829||RR|unclassified|R|Orphan|... at 55%

1 718aa, >Adeh_3688||RR|unclassified|R|Orphan|... at 64%

2 714aa, >AnaeK_3746||RR|unclassified|R|Orphan|... at 55%

3 735aa, >Anae109_3814||RR|unclassified|R|Orphan|... *

>Cluster 184

0 691aa, >LILAB_07150||HK|Classic|T|Orphan|... at 56%

1 714aa, >MXAN_0304||HK|Classic|T|Orphan|... at 53%

2 717aa, >COCOR_07739||HK|Classic|T|Orphan|... *

3 695aa, >A176_6281||HK|Classic|T||... at 54%

>Cluster 185$

0 696aa, >LILAB_29400||HK|Classic|T|Complex|... at 70%

1 707aa, >MXAN_4251||HK|Classic|T|Complex|... at 69%

2 711aa, >COCOR_03677||HK|Classic|T|Pair|... *

3 707aa, >A176_4122||HK|Classic|T||... at 69%

>Cluster 186$

0 683aa, >MXAN_6964||HK|CheA|T|Complex|... at 59%

1 692aa, >COCOR_07538||HK|CheA|T|Complex|... at 63%

2 697aa, >STAUR_0860||HK|CheA|T|Pair|... *

3 690aa, >A176_5480||HK|CheA|T||... at 65%

>Cluster 187

0 684aa, >A2cp1_4471||HK|CheA|T|Pair|... *

1 674aa, >Adeh_4316||HK|CheA|T|Pair|... at 94%

2 680aa, >AnaeK_4452||HK|CheA|T|Pair|... at 98%

3 662aa, >Anae109_4461||HK|CheA|T|Pair|... at 62%

>Cluster 188

0 646aa, >A2cp1_4382||HK|Classic|T|Complex|... at 68%

1 613aa, >Adeh_4226||HK|Classic|T|Complex|... at 71%

2 646aa, >AnaeK_4359||HK|Classic|T|Complex|... at 68%

3 667aa, >Anae109_4376||HK|Classic|T|Complex|... *

>Cluster 189

0 662aa, >A2cp1_1965||HK|Classic|T|Pair|... *

1 658aa, >Adeh_1998||HK|Classic|T|Pair|... at 93%

2 662aa, >AnaeK_1880||HK|Classic|T|Pair|... at 98%

3 640aa, >Anae109_1809||HK|Classic|T|Pair|... at 62%

>Cluster 190

0 589aa, >A2cp1_4467||HK|Classic|T|Orphan|... at 58%

1 591aa, >Adeh_4312||HK|Classic|T|Orphan|... at 58%

2 589aa, >AnaeK_4448||HK|Classic|T|Orphan|... at 58%

3 661aa, >Anae109_4457||HK|Classic|T|Orphan|... *

>Cluster 191

0 660aa, >LILAB_21740||RR|RpfG|R|Orphan|... *

1 660aa, >MXAN_2807||RR|RpfG|R|Orphan|... at 96%

2 656aa, >COCOR_05254||RR|unclassified|R|Orphan|... at 82%

3 653aa, >STAUR_5245||RR|unclassified|R|Orphan|... at 66%

>Cluster 192

0 627aa, >A2cp1_4378||HK|Hybrid|RTR|Complex|... at 77%

1 627aa, >Adeh_4222||HK|Hybrid|RTR|Complex|... at 77%

2 638aa, >AnaeK_4355||HK|Hybrid|RTR|Complex|... at 77%

3 641aa, >Anae109_4372||HK|Hybrid|RTR|Complex|... *

>Cluster 193

0 627aa, >LILAB_31515||RR|unclassified|R|Orphan|... at 93%

1 630aa, >MXAN_4717||RR|unclassified|R|Orphan|... *

2 628aa, >COCOR_02678||RR|CheY|R|Orphan|... at 76%

3 630aa, >A176_6044||RR|CheY|R||... at 93%

>Cluster 194

0 622aa, >A2cp1_0598||RR|PleD|R|Complex|... *

1 622aa, >Adeh_0571||RR|PleD|R|Complex|... at 98%

2 622aa, >AnaeK_0606||RR|PleD|R|Complex|... at 99%

3 622aa, >Anae109_0614||RR|PleD|R|Complex|... at 75%

>Cluster 195

0 614aa, >LILAB_05110||HK|Classic|T|Orphan|... at 63%

1 614aa, >MXAN_0736||HK|Classic|T|Orphan|... at 62%

2 618aa, >COCOR_00618||HK|Classic|T|Orphan|... *

3 614aa, >A176_3376||HK|Classic|T||... at 62%

>Cluster 196$

0 615aa, >MXAN_0168||HK|Classic|T|Orphan|... *

1 611aa, >COCOR_07862||HK|Classic|T|Pair|... at 73%

2 606aa, >STAUR_0221||HK|Classic|T|Pair|... at 73%

3 615aa, >A176_3143||HK|Classic|T||... at 88%

>Cluster 197$

0 594aa, >LILAB_29435||RR|PleD|R|Orphan|... at 68%

1 594aa, >MXAN_4257||RR|PleD|R|Orphan|... at 68%

2 599aa, >COCOR_03614||RR|PleD|R|Complex|... *

3 594aa, >A176_4129||RR|PleD|R||... at 70%

>Cluster 198

0 563aa, >LILAB_28905||RR|unclassified|R|Orphan|... at 51%

1 562aa, >MXAN_4149|FrzS|RR|unclassified|R|Orphan|... at 52%

2 593aa, >STAUR_4596||RR|unclassified|R|Orphan|... *

3 577aa, >A176_1854||RR|unclassified|R||... at 51%

>Cluster 199

0 592aa, >A2cp1_3864||HK|Classic|T|Orphan|... *

1 592aa, >Adeh_3724||HK|Classic|T|Orphan|... at 94%

2 592aa, >AnaeK_3781||HK|Classic|T|Orphan|... at 99%

3 591aa, >Anae109_3853||HK|Classic|T|Orphan|... at 70%

>Cluster 200

0 587aa, >A2cp1_0806||HK|Classic|T|Orphan|... at 53%

1 588aa, >Adeh_0755||HK|Classic|T|Orphan|... at 53%

2 587aa, >AnaeK_0802||HK|Classic|T|Orphan|... at 53%

3 592aa, >Anae109_0792||HK|Classic|T|Orphan|... *

>Cluster 201

0 581aa, >A2cp1_3487||HK|Classic|T|Orphan|... *

1 581aa, >Adeh_3342||HK|Classic|T|Orphan|... at 93%

2 581aa, >AnaeK_3423||HK|Classic|T|Orphan|... at 97%

3 573aa, >Anae109_3408||HK|Classic|T|Orphan|... at 70%

>Cluster 202

0 513aa, >LILAB_15675||HK|Classic|T|Orphan|... at 89%

1 565aa, >MXAN_6117||HK|Classic|T|Orphan|... *

2 486aa, >COCOR_03804||HK|Classic|T|Orphan|... at 68%

3 563aa, >A176_3456||HK|Classic|T||... at 85%

>Cluster 203

0 490aa, >A2cp1_0323||HK|Classic|T|Pair|... at 95%

1 562aa, >Adeh_0301||HK|Classic|T|Pair|... *

2 490aa, >AnaeK_0312||HK|Classic|T|Pair|... at 95%

3 506aa, >Anae109_4258||HK|Classic|T|Pair|... at 72%

>Cluster 204^

0 560aa, >A2cp1_3169||HK|Classic|T|Pair|... *

1 560aa, >AnaeK_3061||HK|Classic|T|Pair|... at 99%

2 559aa, >Anae109_1441||HK|Classic|T|Pair|... at 68%

3 559aa, >Anae109_3417||HK|Classic|T|Pair|... at 62%

>Cluster 205

0 547aa, >STAUR_4722||HK|CheA|T|Pair|... at 51%

1 553aa, >A2cp1_0628||HK|CheA|T|Orphan|... *

2 551aa, >Adeh_0603||HK|CheA|T|Orphan|... at 96%

3 551aa, >AnaeK_0637||HK|CheA|T|Orphan|... at 99%

>Cluster 206$

0 542aa, >LILAB_16225||HK|Classic|T|Orphan|... at 88%

1 548aa, >MXAN_6015||HK|Classic|T|Orphan|... *

2 534aa, >COCOR_06545||HK|Classic|T|Pair|... at 60%

3 547aa, >A176_1099||HK|Classic|T||... at 83%

>Cluster 207

0 525aa, >A2cp1_0659||HK|Classic|T|Pair|... at 57%

1 526aa, >Adeh_0625||HK|Classic|T|Pair|... at 57%

2 525aa, >AnaeK_0659||HK|Classic|T|Pair|... at 56%

3 546aa, >Anae109_0670||HK|Classic|T|Pair|... *

>Cluster 208

0 475aa, >A2cp1_2835||RR|NtrC|R|Orphan|... at 83%

1 483aa, >Adeh_2651||RR|NtrC|R|Orphan|... at 82%

2 475aa, >AnaeK_2740||RR|NtrC|R|Orphan|... at 83%

3 532aa, >Anae109_2620||RR|NtrC|R|Orphan|... *

>Cluster 209

0 525aa, >A2cp1_1199||HK|Classic|T|Orphan|... *

1 498aa, >Adeh_1071||HK|Classic|T|Orphan|... at 96%

2 525aa, >AnaeK_1130||HK|Classic|T|Orphan|... at 97%

3 508aa, >Anae109_1110||HK|Classic|T|Orphan|... at 75%

>Cluster 210

0 523aa, >A2cp1_3624||HK|Hybrid|RT|Complex|... *

1 520aa, >Adeh_3472||HK|Hybrid|RT|Complex|... at 97%

2 523aa, >AnaeK_3556||HK|Hybrid|RT|Complex|... at 99%

3 516aa, >Anae109_3582||HK|Hybrid|RT|Complex|... at 72%

>Cluster 211

0 516aa, >A2cp1_0604||HK|Classic|T|Orphan|... at 77%

1 516aa, >Adeh_0577||HK|Classic|T|Orphan|... at 77%

2 516aa, >AnaeK_0612||HK|Classic|T|Orphan|... at 77%

3 518aa, >Anae109_0620||HK|Classic|T|Orphan|... *

>Cluster 212

0 479aa, >LILAB_03610||HK|Classic|T|Orphan|... at 95%

1 513aa, >MXAN_1014|SdeK|HK|Classic|T|Orphan|... *

2 488aa, >STAUR_6987||HK|Classic|T|Orphan|... at 52%

3 477aa, >A176_3943||HK|Classic|T||... at 90%

>Cluster 213

0 434aa, >A2cp1_2397||RR|PleD|R|Complex|... at 67%

1 300aa, >Adeh_1556||RR|PleD|R|Complex|... at 79%

2 434aa, >AnaeK_2309||RR|PleD|R|Complex|... at 67%

3 509aa, >Anae109_2257||RR|PleD|R|Complex|... *

>Cluster 214

0 485aa, >LILAB_10475||HK|Classic|T|Pair|... at 84%

1 507aa, >MXAN_7180|Orf2|HK|Classic|T|Pair|... *

2 496aa, >COCOR_00234||HK|Classic|T|Pair|... at 56%

3 486aa, >A176_5711||HK|Classic|T||... at 84%

>Cluster 215

0 501aa, >LILAB_11630||HK|Classic|T|Complex|... at 64%

1 501aa, >MXAN_6955|TodK|HK|Classic|T|Complex|... at 63%

2 505aa, >COCOR_07532||HK|Classic|T|Complex|... *

3 501aa, >A176_5471||HK|Classic|T||... at 63%

>Cluster 216

0 502aa, >LILAB_12950||HK|Classic|T|Orphan|... *

1 502aa, >MXAN_6702||HK|Classic|T|Orphan|... at 94%

2 497aa, >COCOR_07290||HK|Classic|T|Orphan|... at 78%

3 498aa, >A176_2248||HK|Classic|T||... at 92%

>Cluster 217£

0 502aa, >A2cp1_1970||HK|Classic|T|Orphan|... *

1 457aa, >Adeh_1993||HK|Classic|T|Pair|... at 97%

2 502aa, >AnaeK_1885||HK|Classic|T|Complex|... at 100%

3 501aa, >Anae109_1846||HK|Classic|T|Complex|... at 76%

>Cluster 218

0 481aa, >A2cp1_1183||RR|PrrA|R|Orphan|... at 98%

1 481aa, >Adeh_1055||RR|PrrA|R|Orphan|... at 97%

2 501aa, >AnaeK_1114||RR|PrrA|R|Orphan|... *

3 475aa, >Anae109_1105||RR|NtrC|R|Orphan|... at 85%

>Cluster 219

0 498aa, >A2cp1_0125||HK|Classic|T|Pair|... *

1 497aa, >Adeh_0107||HK|Classic|T|Pair|... at 98%

2 498aa, >AnaeK_0114||HK|Classic|T|Pair|... at 97%

3 497aa, >Anae109_0111||HK|Classic|T|Pair|... at 80%

>Cluster 220

0 487aa, >A2cp1_4368||RR|unclassified|R|Orphan|... at 52%

1 487aa, >Adeh_4213||RR|unclassified|R|Orphan|... at 51%

2 487aa, >AnaeK_4345||RR|unclassified|R|Orphan|... at 52%

3 493aa, >Anae109_4361||RR|unclassified|R|Orphan|... *

>Cluster 221

0 485aa, >A2cp1_3792||RR|NtrC|R|Orphan|... at 94%

1 492aa, >Adeh_3651||RR|NtrC|R|Orphan|... *

2 486aa, >AnaeK_3709||RR|NtrC|R|Orphan|... at 95%

3 474aa, >Anae109_3777||RR|NtrC|R|Orphan|... at 71%

>Cluster 222

0 441aa, >LILAB_35995||HK|Classic|T|Orphan|... at 90%

1 488aa, >MXAN_5704||HK|Classic|T|Orphan|... *

2 471aa, >COCOR_06182||HK|Classic|T|Orphan|... at 62%

3 455aa, >A176_7193||HK|Classic|T||... at 82%

>Cluster 223

0 472aa, >LILAB_30890||RR|NtrC|R|Pair|... at 71%

1 475aa, >COCOR_02144||RR|NtrC|R|Pair|... at 63%

2 488aa, >STAUR_5454||RR|NtrC|R|Pair|... *

3 473aa, >A176_1901||RR|NtrC|R||... at 71%

>Cluster 224

0 488aa, >A2cp1_0599||RR|unclassified|R|Complex|... *

1 488aa, >Adeh_0572||RR|unclassified|R|Complex|... at 98%

2 488aa, >AnaeK_0607||RR|unclassified|R|Complex|... at 99%

3 485aa, >Anae109_0615||RR|unclassified|R|Complex|... at 75%

>Cluster 225

0 486aa, >LILAB_03745||HK|Hybrid|RT|Pair|... *

1 486aa, >MXAN_0993||HK|Hybrid|RT|Pair|... at 91%

2 486aa, >COCOR_00929||HK|Hybrid|RT|Pair|... at 67%

3 486aa, >A176_3918||HK|Hybrid|RT||... at 86%

>Cluster 226

0 460aa, >LILAB_16020||HK|Classic|T|Orphan|... at 91%

1 485aa, >MXAN_6053||HK|Classic|T|Orphan|... *

2 473aa, >STAUR_7421||HK|Classic|T|Orphan|... at 59%

3 461aa, >A176_1139||HK|Classic|T||... at 86%

>Cluster 227

0 471aa, >A2cp1_4402||HK|Classic|T|Orphan|... at 77%

1 469aa, >Adeh_4246||HK|Classic|T|Orphan|... at 78%

2 471aa, >AnaeK_4379||HK|Classic|T|Orphan|... at 77%

3 484aa, >Anae109_4395||HK|Classic|T|Orphan|... *

>Cluster 228£

0 483aa, >A2cp1_4406||RR|NtrC|R|Pair|... at 84%

1 483aa, >Adeh_4250||RR|NtrC|R|Orphan|... at 84%

2 483aa, >AnaeK_4383||RR|NtrC|R|Pair|... at 84%

3 484aa, >Anae109_4399||RR|NtrC|R|Pair|... *

>Cluster 229

0 459aa, >A2cp1_0922||RR|unclassified|R|Orphan|... at 92%

1 483aa, >Adeh_0873||RR|unclassified|R|Orphan|... *

2 458aa, >AnaeK_0918||RR|unclassified|R|Orphan|... at 93%

3 456aa, >Anae109_0930||RR|unclassified|R|Orphan|... at 63%

>Cluster 230

0 473aa, >LILAB_10680||RR|NtrC|R|Pair|... at 95%

1 477aa, >MXAN_7143||RR|NtrC|R|Pair|... *

2 472aa, >COCOR_00261||RR|NtrC|R|Pair|... at 84%

3 473aa, >A176_5679||RR|NtrC|R||... at 94%

>Cluster 231

0 405aa, >A2cp1_0278||HK|Classic|T|Orphan|... at 59%

1 405aa, >Adeh_0256||HK|Classic|T|Orphan|... at 59%

2 405aa, >AnaeK_0267||HK|Classic|T|Orphan|... at 59%

3 471aa, >Anae109_0278||HK|Classic|T|Orphan|... *

>Cluster 232

0 447aa, >LILAB_10685||HK|Classic|T|Pair|... at 73%

1 447aa, >MXAN_7142||HK|Classic|T|Pair|... at 72%

2 469aa, >COCOR_00262||HK|Classic|T|Pair|... *

3 434aa, >A176_5678||HK|Classic|T||... at 74%

>Cluster 233$

0 453aa, >MXAN_0172||RR|NtrC|R|Orphan|... at 86%

1 445aa, >COCOR_07860||RR|NtrC|R|Pair|... at 86%

2 467aa, >STAUR_0222||RR|NtrC|R|Pair|... *

3 446aa, >A176_3139||RR|NtrC|R||... at 86%

>Cluster 234

0 460aa, >MXAN_5853|Nla1|RR|unclassified|R|Pair|... at 60%

1 461aa, >COCOR_06378||RR|unclassified|R|Pair|... at 59%

2 459aa, >A176_4389||RR|unclassified|R||... at 60%

3 465aa, >sce2692||RR|unclassified|R|Orphan|... *

>Cluster 235

0 411aa, >LILAB_12230||HK|Hybrid|RT|Pair|... at 87%

1 464aa, >MXAN_6847||HK|Hybrid|RT|Pair|... *

2 449aa, >COCOR_04346||HK|Hybrid|RT|Pair|... at 63%

3 411aa, >A176_3558||HK|Hybrid|RT||... at 81%

>Cluster 236

0 461aa, >A2cp1_4476||HK|Classic|T|Orphan|... at 81%

1 461aa, >Adeh_4321||HK|Classic|T|Orphan|... at 80%

2 461aa, >AnaeK_4457||HK|Classic|T|Orphan|... at 81%

3 464aa, >Anae109_4466||HK|Classic|T|Orphan|... *

>Cluster 237

0 462aa, >LILAB_30325||RR|unclassified|R|Orphan|... *

1 457aa, >MXAN_4438||RR|unclassified|R|Orphan|... at 90%

2 452aa, >COCOR_03344||RR|CheY|R|Orphan|... at 68%

3 462aa, >A176_5115||RR|CheY|R||... at 86%

>Cluster 238

0 459aa, >A2cp1_0124||RR|NtrC|R|Pair|... *

1 459aa, >Adeh_0106||RR|NtrC|R|Pair|... at 98%

2 459aa, >AnaeK_0113||RR|NtrC|R|Pair|... at 96%

3 457aa, >Anae109_0110||RR|NtrC|R|Pair|... at 85%

>Cluster 239$

0 455aa, >LILAB_29405||RR|NtrC|R|Complex|... *

1 455aa, >MXAN_4252|Nla12|RR|NtrC|R|Complex|... at 94%

2 447aa, >COCOR_03676||RR|NtrC|R|Pair|... at 80%

3 455aa, >A176_4123||RR|NtrC|R||... at 94%

>Cluster 240

0 428aa, >A2cp1_3107||HK|Classic|T|Pair|... at 63%

1 429aa, >Adeh_2915||HK|Classic|T|Pair|... at 65%

2 429aa, >AnaeK_3001||HK|Classic|T|Pair|... at 63%

3 453aa, >Anae109_0881||HK|Classic|T|Pair|... *

>Cluster 241

0 450aa, >LILAB_02650||RR|NtrC|R|Pair|... at 84%

1 450aa, >MXAN_1189||RR|NtrC|R|Pair|... at 83%

2 451aa, >COCOR_07104||RR|NtrC|R|Pair|... *

3 450aa, >A176_0536||RR|NtrC|R||... at 84%

>Cluster 242

0 412aa, >A2cp1_2815||HK|Classic|T|Pair|... at 95%

1 451aa, >Adeh_2634||HK|Classic|T|Pair|... *

2 407aa, >AnaeK_2720||HK|Classic|T|Pair|... at 95%

3 448aa, >Anae109_2597||HK|Classic|T|Pair|... at 64%

>Cluster 243£

0 443aa, >A2cp1_0533||RR|NtrC|R|Complex|... at 99%

1 443aa, >Adeh_0504||RR|NtrC|R|Complex|... at 98%

2 446aa, >AnaeK_0537||RR|NtrC|R|Complex|... *

3 446aa, >Anae109_2379||RR|NtrC|R|Pair|... at 76%

>Cluster 244

0 423aa, >LILAB_02645||HK|Classic|T|Pair|... at 93%

1 424aa, >MXAN_1190||HK|Classic|T|Pair|... *

2 400aa, >COCOR_07103||HK|Classic|T|Pair|... at 76%

3 424aa, >A176_0537||HK|Classic|T||... at 85%

>Cluster 245$

0 379aa, >LILAB_32800||RR|unclassified|R|Complex|... at 89%

1 419aa, >MXAN_4975||RR|unclassified|R|Complex|... *

2 379aa, >COCOR_02478||RR|CheY|R|Orphan|... at 52%

3 387aa, >A176_3029||RR|CheY|R||... at 83%

>Cluster 246

0 417aa, >A2cp1_2474||HK|Hybrid|RT|Pair|... at 95%

1 418aa, >Adeh_1481||HK|Hybrid|RT|Pair|... *

2 417aa, >AnaeK_2386||HK|Hybrid|RT|Pair|... at 95%

3 418aa, >Anae109_2352||HK|Hybrid|RT|Pair|... at 70%

>Cluster 247£

0 414aa, >A2cp1_0532||HK|Classic|T|Complex|... at 64%

1 414aa, >Adeh_0503||HK|Classic|T|Complex|... at 64%

2 414aa, >AnaeK_0536||HK|Classic|T|Complex|... at 64%

3 418aa, >Anae109_2378||HK|Classic|T|Pair|... *

>Cluster 248

0 381aa, >A2cp1_2325||HK|Classic|T|Orphan|... at 66%

1 377aa, >Adeh_1623||HK|Classic|T|Orphan|... at 67%

2 381aa, >AnaeK_2237||HK|Classic|T|Orphan|... at 66%

3 413aa, >Anae109_2188||HK|Classic|T|Orphan|... *

>Cluster 249

0 404aa, >LILAB_03700||HK|Hybrid|RT|Complex|... *

1 396aa, >COCOR_00948||HK|Hybrid|RT|Complex|... at 63%

2 397aa, >STAUR_7004||HK|Hybrid|RT|Complex|... at 57%

3 398aa, >A176_3927||HK|Hybrid|RT||... at 78%

>Cluster 250

0 388aa, >LILAB_36050||HK|Hybrid|RT|Pair|... at 96%

1 399aa, >MXAN_5715||HK|Hybrid|RT|Pair|... *

2 392aa, >COCOR_06192||HK|Hybrid|RT|Orphan|... at 75%

3 388aa, >A176_3405||HK|Hybrid|RT||... at 86%

>Cluster 251

0 379aa, >LILAB_23605||HK|Hybrid|RT|Complex|... at 56%

1 392aa, >COCOR_05186||HK|Hybrid|RT|Complex|... *

2 376aa, >STAUR_3490||HK|Hybrid|RT|Complex|... at 54%

3 369aa, >A176_6768||HK|Hybrid|RT||... at 56%

>Cluster 252

0 386aa, >LILAB_05205||RR|unclassified|R|Orphan|... *

1 378aa, >MXAN_0715||RR|unclassified|R|Orphan|... at 89%

2 304aa, >STAUR_7572||RR|CheY|R|Orphan|... at 66%

3 386aa, >A176_3395||RR|unclassified|R||... at 84%

>Cluster 253

0 373aa, >A2cp1_0672||HK|Classic|T|Pair|... at 60%

1 373aa, >Adeh_0638||HK|Classic|T|Pair|... at 59%

2 373aa, >AnaeK_0672||HK|Classic|T|Pair|... at 60%

3 380aa, >Anae109_0683||HK|Classic|T|Pair|... *

>Cluster 254£

0 377aa, >A2cp1_1514||HK|Classic|T|Pair|... *

1 367aa, >Adeh_2444||HK|Classic|T|Pair|... at 91%

2 365aa, >AnaeK_0373||HK|Classic|T|Complex|... at 67%

3 365aa, >Anae109_3188||HK|Classic|T|Orphan|... at 70%

>Cluster 255

0 376aa, >A2cp1_4150||HK|Classic|T|Pair|... at 77%

1 376aa, >Adeh_4008||HK|Classic|T|Pair|... at 77%

2 376aa, >AnaeK_4124||HK|Classic|T|Pair|... at 77%

3 377aa, >Anae109_0416||HK|Classic|T|Pair|... *

>Cluster 256

0 365aa, >LILAB_22650||HK|Hybrid|RT|Pair|... *

1 358aa, >COCOR_05836||HK|Hybrid|RT|Pair|... at 66%

2 357aa, >STAUR_4544||HK|Hybrid|RT|Pair|... at 63%

3 365aa, >A176_5825||HK|Hybrid|RT||... at 78%

>Cluster 257

0 354aa, >LILAB_02325||HK|Classic|T|Pair|... *

1 353aa, >MXAN_1280||HK|Classic|T|Pair|... at 92%

2 352aa, >COCOR_01188||HK|Classic|T|Pair|... at 77%

3 351aa, >A176_0591||HK|Classic|T||... at 88%

>Cluster 258

0 322aa, >A2cp1_3625||RR|PleD|R|Complex|... at 97%

1 351aa, >Adeh_3473||RR|PleD|R|Complex|... *

2 322aa, >AnaeK_3557||RR|PleD|R|Complex|... at 97%

3 322aa, >Anae109_3583||RR|PleD|R|Complex|... at 68%

>Cluster 259

0 311aa, >LILAB_34660||RR|PleD|R|Complex|... at 61%

1 320aa, >MXAN_5366||RR|PleD|R|Complex|... at 61%

2 316aa, >COCOR_02204||RR|PleD|R|Complex|... at 58%

3 349aa, >STAUR_6073||RR|PleD|R|Complex|... *

>Cluster 260

0 310aa, >LILAB_23845||RR|PleD|R|Complex|... at 96%

1 342aa, >MXAN_3213|ActA|RR|PleD|R|Complex|... *

2 317aa, >COCOR_02962||RR|PleD|R|Complex|... at 75%

3 310aa, >A176_6380||RR|PleD|R||... at 94%

>Cluster 261

0 330aa, >LILAB_28850|FrzG|RR|CheB|R|Complex|... at 74%

1 334aa, >MXAN_4139|FrzG|RR|CheB|R|Complex|... at 74%

2 342aa, >COCOR_03836||RR|CheB|R|Complex|... *

3 329aa, >STAUR_4586||RR|CheB|R|Complex|... at 64%

>Cluster 262

0 341aa, >A2cp1_0639||RR|CheB|R|Complex|... *

1 341aa, >Adeh_0614||RR|CheB|R|Complex|... at 97%

2 341aa, >AnaeK_0648||RR|CheB|R|Complex|... at 99%

3 340aa, >Anae109_0658||RR|CheB|R|Complex|... at 76%

>Cluster 263

0 332aa, >LILAB_04060||HK|Classic|T|Orphan|... at 95%

1 334aa, >MXAN_0928|HpkA|HK|Classic|T|Orphan|... *

2 329aa, >COCOR_00879||HK|Classic|T|Orphan|... at 80%

3 323aa, >A176_0936||HK|Classic|T||... at 91%

>Cluster 264

0 300aa, >A2cp1_0439||HK|Classic|T|Pair|... at 74%

1 300aa, >Adeh_0410||HK|Classic|T|Pair|... at 74%

2 307aa, >AnaeK_0438||HK|Classic|T|Pair|... at 72%

3 311aa, >Anae109_4160||HK|Classic|T|Pair|... *

>Cluster 265

0 307aa, >A2cp1_2399||HK|Classic|T|Complex|... *

1 307aa, >Adeh_1554||PP|HisKa|H|Complex|... at 89%

2 307aa, >AnaeK_2311||HK|Classic|T|Complex|... at 99%

3 306aa, >Anae109_2259||PP|HisKa|H|Complex|... at 64%

>Cluster 266

0 279aa, >A2cp1_3886||RR|unclassified|R|Orphan|... at 96%

1 280aa, >Adeh_3745||RR|unclassified|R|Orphan|... *

2 279aa, >AnaeK_3802||RR|unclassified|R|Orphan|... at 96%

3 180aa, >Anae109_3862||RR|CheY|R|Orphan|... at 52%

>Cluster 267

0 248aa, >A2cp1_4356||RR|unclassified|R|Orphan|... at 97%

1 262aa, >Adeh_4201||RR|unclassified|R|Orphan|... *

2 248aa, >AnaeK_4333||RR|unclassified|R|Orphan|... at 97%

3 248aa, >Anae109_4350||RR|unclassified|R|Orphan|... at 66%

>Cluster 268

0 257aa, >LILAB_02330||RR|LytTR|R|Pair|... *

1 214aa, >MXAN_1279||RR|LytTR|R|Pair|... at 92%

2 257aa, >COCOR_01187||RR|LytTR|R|Pair|... at 80%

3 257aa, >A176_0590||RR|LytTR|R||... at 85%

>Cluster 269

0 249aa, >LILAB_06395||RR|FrzZ|RR|Complex|... at 98%

1 255aa, >MXAN_0460|RedD|RR|FrzZ|RR|Complex|... *

2 244aa, >STAUR_7970||RR|FrzZ|RR|Complex|... at 65%

3 249aa, >A176_1436||RR|FrzZ|RR||... at 97%

>Cluster 270£

0 248aa, >A2cp1_1523||RR|unclassified|R|Orphan|... *

1 248aa, >Adeh_2435||RR|unclassified|R|Orphan|... at 96%

2 248aa, >AnaeK_1428||RR|unclassified|R|Orphan|... at 100%

3 241aa, >Anae109_0025||RR|unclassified|R|Complex|... at 53%

>Cluster 271

0 245aa, >A2cp1_4381||HK|Classic|T|Complex|... at 61%

1 245aa, >Adeh_4225||HK|Classic|T|Complex|... at 60%

2 245aa, >AnaeK_4358||HK|Classic|T|Complex|... at 62%

3 246aa, >Anae109_4375||HK|Classic|T|Complex|... *

>Cluster 272^

0 239aa, >A2cp1_3170||RR|LytTR|R|Pair|... *

1 239aa, >AnaeK_3062||RR|LytTR|R|Pair|... at 99%

2 236aa, >Anae109_1440||RR|LytTR|R|Pair|... at 74%

3 237aa, >Anae109_3416||RR|LytTR|R|Pair|... at 58%

>Cluster 273

0 119aa, >MXAN_7364||RR|CheY|R|Complex|... at 57%

1 124aa, >COCOR_07950||RR|CheY|R|Complex|... at 59%

2 236aa, >PPSIR1_07475||RR|unclassified|R||... *

3 125aa, >A176_3696||RR|CheY|R||... at 57%

>Cluster 274

0 229aa, >LILAB_16310||RR|OmpR|R|Pair|... at 95%

1 231aa, >MXAN_5995||RR|OmpR|R|Pair|... *

2 229aa, >PPSIR1_07078||RR|OmpR|R||... at 54%

3 231aa, >A176_1082||RR|OmpR|R||... at 96%

>Cluster 275

0 230aa, >A2cp1_0634||RR|unclassified|R|Orphan|... *

1 191aa, >Adeh_0609||RR|unclassified|R|Orphan|... at 98%

2 230aa, >AnaeK_0643||RR|unclassified|R|Orphan|... at 99%

3 230aa, >Anae109_0653||RR|unclassified|R|Orphan|... at 77%

>Cluster 276&

0 156aa, >LILAB_04025||RR|CheY|R|Orphan|... at 96%

1 228aa, >MXAN_0935|OrfF|RR|unclassified|R|Orphan|... *

2 152aa, >COCOR_00888||RR|CheY|R|Orphan|... at 55%

3 156aa, >A176_0944||RR|CheY|R||... at 91%

>Cluster 277$

0 224aa, >LILAB_26865||RR|unclassified|R|Complex|... at 72%

1 224aa, >MXAN_3738||RR|unclassified|R|Orphan|... at 75%

2 223aa, >COCOR_04264||RR|CheY|R|Orphan|... at 51%

3 225aa, >A176_6148||RR|CheY|R||... *

>Cluster 278

0 224aa, >LILAB_26585||RR|OmpR|R|Pair|... *

1 224aa, >COCOR_04326||RR|OmpR|R|Pair|... at 83%

2 224aa, >STAUR_0009||RR|OmpR|R|Pair|... at 74%

3 224aa, >A176_6204||RR|OmpR|R||... at 88%

>Cluster 279

0 218aa, >A2cp1_1966||RR|NarL|R|Pair|... *

1 218aa, >Adeh_1997||RR|NarL|R|Pair|... at 97%

2 218aa, >AnaeK_1881||RR|NarL|R|Pair|... at 100%

3 218aa, >Anae109_1810||RR|NarL|R|Pair|... at 84%

>Cluster 280

0 215aa, >A2cp1_2938||RR|NarL|R|Complex|... *

1 215aa, >Adeh_2754||RR|NarL|R|Orphan|... at 99%

2 215aa, >AnaeK_2846||RR|NarL|R|Orphan|... at 100%

3 215aa, >Anae109_2743||RR|NarL|R|Orphan|... at 93%

>Cluster 281

0 207aa, >A2cp1_3714||RR|MerR|R|Orphan|... at 67%

1 207aa, >Adeh_3574||RR|MerR|R|Orphan|... at 66%

2 207aa, >AnaeK_3640||RR|MerR|R|Orphan|... at 68%

3 210aa, >Anae109_3695||RR|MerR|R|Orphan|... *

>Cluster 282

0 209aa, >MXAN_0311||RR|NarL|R|Orphan|... *

1 209aa, >COCOR_07728||RR|NarL|R|Orphan|... at 75%

2 208aa, >AnaeK_0902||RR|NarL|R|Orphan|... at 51%

3 202aa, >Anae109_0915||RR|NarL|R|Orphan|... at 52%

>Cluster 283

0 204aa, >A2cp1_4119A||RR|unclassified|R|Orphan|... *

1 123aa, >Adeh_3975||RR|CheY|R|Orphan|... at 93%

2 123aa, >AnaeK_4086||RR|CheY|R|Orphan|... at 99%

3 126aa, >Anae109_0442||RR|CheY|R|Orphan|... at 62%

>Cluster 284

0 136aa, >A2cp1_4269||RR|CheY|R|Orphan|... at 56%

1 136aa, >Adeh_4116||RR|CheY|R|Orphan|... at 56%

2 136aa, >AnaeK_4246||RR|CheY|R|Orphan|... at 56%

3 197aa, >Anae109_4274||RR|unclassified|R|Orphan|... *

>Cluster 285

0 135aa, >A2cp1_0609||RR|CheY|R|Orphan|... at 93%

1 169aa, >Adeh_0582||RR|CheY|R|Orphan|... *

2 135aa, >AnaeK_0617||RR|CheY|R|Orphan|... at 92%

3 121aa, >Anae109_0625||RR|CheY|R|Orphan|... at 80%

>Cluster 286

0 124aa, >A2cp1_4295||RR|CheY|R|Orphan|... at 79%

1 124aa, >Adeh_4142||RR|CheY|R|Orphan|... at 78%

2 124aa, >AnaeK_4273||RR|CheY|R|Orphan|... at 79%

3 168aa, >Anae109_4285||RR|CheY|R|Orphan|... *

>Cluster 287

0 113aa, >A2cp1_0445||RR|CheY|R|Complex|... at 62%

1 113aa, >Adeh_0416||RR|CheY|R|Complex|... at 61%

2 113aa, >AnaeK_0444||RR|CheY|R|Complex|... at 61%

3 150aa, >Anae109_4153||RR|CheY|R|Complex|... *

>Cluster 288

0 148aa, >LILAB_03705||RR|CheY|R|Complex|... *

1 145aa, >COCOR_00947||RR|CheY|R|Complex|... at 73%

2 146aa, >STAUR_7005||RR|CheY|R|Complex|... at 64%

3 142aa, >A176_3926||RR|CheY|R||... at 84%

>Cluster 289

0 126aa, >LILAB_29410||RR|CheY|R|Complex|... at 69%

1 131aa, >MXAN_4253||RR|CheY|R|Complex|... at 68%

2 143aa, >COCOR_03615||RR|CheY|R|Complex|... *

3 128aa, >A176_4124||RR|CheY|R||... at 67%

>Cluster 290£

0 119aa, >A2cp1_2989||RR|CheY|R|Complex|... at 58%

1 119aa, >Adeh_2803||RR|CheY|R|Pair|... at 57%

2 119aa, >AnaeK_2898||RR|CheY|R|Pair|... at 58%

3 136aa, >Anae109_2756||RR|CheY|R|Orphan|... *

>Cluster 291

0 127aa, >A2cp1_1620||RR|CheY|R|Orphan|... at 100%

1 135aa, >Adeh_2344||RR|CheY|R|Orphan|... *

2 127aa, >AnaeK_1525||RR|CheY|R|Orphan|... at 100%

3 127aa, >Anae109_1530||RR|CheY|R|Orphan|... at 81%

>Cluster 292

0 122aa, >A2cp1_0828||RR|CheY|R|Orphan|... at 63%

1 122aa, >Adeh_0776||RR|CheY|R|Orphan|... at 61%

2 122aa, >AnaeK_0824||RR|CheY|R|Orphan|... at 63%

3 130aa, >Anae109_0824||RR|CheY|R|Orphan|... *

>Cluster 293

0 121aa, >LILAB_05230||RR|CheY|R|Orphan|... at 77%

1 121aa, >MXAN_0710||RR|CheY|R|Orphan|... at 75%

2 129aa, >COCOR_00588||RR|CheY|R|Orphan|... *

3 121aa, >A176_7424||RR|CheY|R||... at 76%

>Cluster 294

0 129aa, >A2cp1_1247||RR|CheY|R|Orphan|... *

1 125aa, >Adeh_1119||RR|CheY|R|Orphan|... at 92%

2 119aa, >AnaeK_1179||RR|CheY|R|Orphan|... at 100%

3 119aa, >Anae109_1159||RR|CheY|R|Orphan|... at 71%

>Cluster 295

0 128aa, >A2cp1_0119||RR|CheY|R|Orphan|... at 64%

1 128aa, >Adeh_0101||RR|CheY|R|Orphan|... at 64%

2 128aa, >AnaeK_0108||RR|CheY|R|Orphan|... at 64%

3 129aa, >Anae109_0102||RR|CheY|R|Orphan|... *

>Cluster 296

0 125aa, >A2cp1_3985||RR|CheY|R|Orphan|... at 62%

1 124aa, >Adeh_3844||RR|CheY|R|Orphan|... at 62%

2 125aa, >AnaeK_3907||RR|CheY|R|Orphan|... at 61%

3 128aa, >Anae109_3956||RR|CheY|R|Orphan|... *

>Cluster 297

0 126aa, >LILAB_06385||RR|CheY|R|Complex|... at 100%

1 127aa, >MXAN_0462|RedF|RR|CheY|R|Complex|... *

2 127aa, >STAUR_7968||RR|CheY|R|Complex|... at 54%

3 126aa, >A176_1434||RR|CheY|R||... at 99%

>Cluster 298

0 120aa, >LILAB_23595||RR|CheY|R|Complex|... at 61%

1 118aa, >COCOR_05188||RR|CheY|R|Complex|... at 53%

2 127aa, >STAUR_3488||RR|CheY|R|Complex|... *

3 120aa, >A176_6770||RR|CheY|R||... at 64%

>Cluster 299

0 126aa, >A2cp1_0162||RR|CheY|R|Orphan|... *

1 126aa, >Adeh_0144||RR|CheY|R|Orphan|... at 99%

2 126aa, >AnaeK_0151||RR|CheY|R|Orphan|... at 100%

3 126aa, >Anae109_0149||RR|CheY|R|Orphan|... at 93%

>Cluster 300$

0 122aa, >LILAB_07355||RR|CheY|R|Complex|... at 75%

1 122aa, >MXAN_0259||RR|CheY|R|Orphan|... at 75%

2 125aa, >COCOR_07785||RR|CheY|R|Complex|... *

3 122aa, >A176_5401||RR|CheY|R||... at 76%

>Cluster 301

0 120aa, >A2cp1_2353||RR|CheY|R|Orphan|... at 68%

1 120aa, >Adeh_1598||RR|CheY|R|Orphan|... at 69%

2 120aa, >AnaeK_2265||RR|CheY|R|Orphan|... at 68%

3 125aa, >Anae109_2215||RR|CheY|R|Orphan|... *

>Cluster 302

0 121aa, >LILAB_04990||RR|CheY|R|Orphan|... at 95%

1 121aa, >MXAN_0763||RR|CheY|R|Orphan|... at 94%

2 119aa, >COCOR_00649||RR|CheY|R|Orphan|... at 73%

3 122aa, >A176_4288||RR|CheY|R||... *

>Cluster 303

0 120aa, >A2cp1_1026||RR|CheY|R|Orphan|... at 100%

1 121aa, >Adeh_0967||RR|CheY|R|Orphan|... *

2 120aa, >AnaeK_1029||RR|CheY|R|Orphan|... at 100%

3 120aa, >Anae109_1012||RR|CheY|R|Orphan|... at 70%

>Cluster 304

0 1764aa, >LILAB_28450||HK|Classic|T|Orphan|... *

1 1764aa, >MXAN_4053||HK|Classic|T|Orphan|... at 94%

2 1763aa, >A176_1767||HK|Classic|T||... at 89%

>Cluster 305

0 1344aa, >LILAB_10330||HK|Hybrid|TR|Pair|... at 74%

1 1379aa, >MXAN_7206|MokA|HK|Hybrid|TR|Pair|... at 71%

2 1411aa, >A176_5738||HK|Hybrid|TR||... *

>Cluster 306£

0 1242aa, >A2cp1_3377||HK|Hybrid|TR|Complex|... at 97%

1 1279aa, >Adeh_3183||HK|Hybrid|TR|Pair|... *

2 1233aa, >AnaeK_0372||HK|Hybrid|TR|Complex|... at 66%

>Cluster 307

0 384aa, >A2cp1_2483||HK|Classic|T|Complex|... at 72%

1 1084aa, >Adeh_1473||HK|Classic|TT|Complex|... *

2 384aa, >AnaeK_2395||HK|Classic|T|Complex|... at 70%

>Cluster 308

0 960aa, >LILAB_21620||HK|Hybrid|TR|Pair|... at 86%

1 1077aa, >MXAN_2785||HK|Hybrid|TR|Pair|... *

2 1010aa, >A176_0223||HK|Hybrid|TR||... at 82%

>Cluster 309

0 959aa, >A2cp1_0763||RR|unclassified|R|Orphan|... at 50%

1 908aa, >Adeh_0726||RR|unclassified|R|Orphan|... at 53%

2 1057aa, >Anae109_0769||RR|unclassified|R|Orphan|... *

>Cluster 310

0 1010aa, >LILAB_09375||HK|Classic|T|Pair|... at 81%

1 999aa, >MXAN_7398||HK|Classic|T|Pair|... at 63%

2 1014aa, >A176_3729||HK|Classic|T||... *

>Cluster 311

0 990aa, >MXAN_5852||HK|Classic|T|Pair|... *

1 984aa, >COCOR_06377||HK|Classic|T|Pair|... at 73%

2 977aa, >A176_4390||HK|Classic|T||... at 90%

>Cluster 312

0 842aa, >MXAN_7363||HK|Hybrid|TRR|Complex|... at 59%

1 933aa, >COCOR_07949||HK|Hybrid|TR|Complex|... *

2 842aa, >A176_3695||HK|Hybrid|TRR||... at 58%

>Cluster 313&

0 729aa, >LILAB_31740||HK|CheA|T|Pair|... at 56%

1 862aa, >MXAN_4758||HK|CheA|T|Pair|... at 54%

2 905aa, >A176_6003||HK|CheA|T||... *

>Cluster 314

0 835aa, >LILAB_04825||HK|Classic|T|Complex|... at 63%

1 882aa, >STAUR_7451||HK|Classic|T|Complex|... *

2 855aa, >A176_4332||HK|Classic|T||... at 61%

>Cluster 315&

0 737aa, >LILAB_13600||HK|Classic|T|Orphan|... at 68%

1 864aa, >MXAN_6586||HK|Classic|T|Orphan|... *

2 740aa, >COCOR_05439||HK|Classic|T|Orphan|... at 58%

>Cluster 316

0 858aa, >A2cp1_0266||RR|unclassified|R|Orphan|... at 98%

1 860aa, >AnaeK_0255||RR|unclassified|R|Orphan|... *

2 851aa, >Anae109_0266||RR|unclassified|R|Orphan|... at 66%

>Cluster 317

0 804aa, >LILAB_05185||HK|Classic|T|Orphan|... at 85%

1 859aa, >MXAN_0720||HK|Classic|T|Orphan|... *

2 801aa, >A176_3391||HK|Classic|T||... at 85%

>Cluster 318

0 850aa, >A2cp1_1747||HK|Hybrid|TR|Pair|... *

1 848aa, >Adeh_2200||HK|Hybrid|TR|Pair|... at 89%

2 850aa, >AnaeK_1676||HK|Hybrid|TR|Pair|... at 97%

>Cluster 319

0 840aa, >A2cp1_1259||HK|Classic|T|Orphan|... *

1 748aa, >Adeh_1130||HK|Classic|T|Orphan|... at 93%

2 746aa, >AnaeK_1190||HK|Classic|T|Orphan|... at 99%

>Cluster 320

0 809aa, >LILAB_07755||HK|Classic|T|Complex|... at 94%

1 838aa, >MXAN_0197||HK|Classic|T|Complex|... *

2 838aa, >A176_3112||HK|Classic|T||... at 91%

>Cluster 321$&

0 723aa, >LILAB_05770||HK|Classic|T|Orphan|... at 51%

1 723aa, >MXAN_0612||HK|Classic|T|Orphan|... at 51%

2 837aa, >STAUR_7785||HK|Hybrid|TR|Complex|... *

>Cluster 322$^

0 818aa, >COCOR_07670||HK|Classic|T|Complex|... at 71%

1 836aa, >COCOR_07671||HK|Classic|T|Complex|... *

2 831aa, >STAUR_2295||HK|Classic|T|Orphan|... at 55%

>Cluster 323

0 804aa, >LILAB_20695||RR|unclassified|R|Complex|... at 92%

1 804aa, >MXAN_2604||RR|unclassified|R|Complex|... at 92%

2 805aa, >A176_4786||RR|CheY|R||... *

>Cluster 324

0 762aa, >LILAB_11380||HK|Classic|T|Complex|... at 84%

1 766aa, >MXAN_7003||HK|Classic|T|Complex|... *

2 766aa, >A176_5530||HK|Classic|T||... at 78%

>Cluster 325

0 755aa, >LILAB_19620||HK|Hybrid|RTR|Complex|... at 90%

1 763aa, >MXAN_2386||HK|Hybrid|RTR|Complex|... *

2 763aa, >A176_2622||HK|Hybrid|TR||... at 83%

>Cluster 326

0 729aa, >A2cp1_2278||HK|Classic|T|Orphan|... at 93%

1 730aa, >Adeh_1668||HK|Classic|T|Orphan|... *

2 729aa, >AnaeK_2189||HK|Classic|T|Orphan|... at 94%

>Cluster 327

0 711aa, >A2cp1_2583||HK|CheA|T|Complex|... at 97%

1 687aa, >Adeh_1370||HK|CheA|T|Complex|... at 67%

2 725aa, >AnaeK_2487||HK|CheA|T|Complex|... *

>Cluster 328

0 712aa, >A2cp1_4275||HK|Hybrid|RT|Complex|... *

1 712aa, >Adeh_4121||HK|Hybrid|RT|Complex|... at 94%

2 712aa, >AnaeK_4252||HK|Hybrid|RT|Complex|... at 98%

>Cluster 329

0 707aa, >A2cp1_0319||HK|Hybrid|TR|Pair|... at 93%

1 711aa, >Adeh_0297||HK|Hybrid|TR|Pair|... *

2 707aa, >AnaeK_0308||HK|Hybrid|TR|Pair|... at 93%

>Cluster 330

0 705aa, >A2cp1_2773||HK|CheA|TR|Complex|... at 95%

1 706aa, >Adeh_1193||HK|CheA|TR|Complex|... *

2 705aa, >AnaeK_2678||HK|CheA|TR|Complex|... at 94%

>Cluster 331

0 702aa, >A2cp1_3134||HK|Hybrid|TR|Complex|... *

1 702aa, >Adeh_2942||HK|Hybrid|TR|Complex|... at 92%

2 702aa, >AnaeK_3028||HK|Hybrid|TR|Complex|... at 96%

>Cluster 332

0 686aa, >LILAB_27975||HK|Hybrid|RT|Pair|... at 89%

1 700aa, >MXAN_3974||HK|Hybrid|RT|Pair|... *

2 699aa, >A176_7284||HK|Hybrid|RT||... at 87%

>Cluster 333

0 655aa, >LILAB_06995||HK|Hybrid|RT|Pair|... at 90%

1 698aa, >MXAN_0336||HK|Hybrid|RT|Pair|... *

2 673aa, >A176_3672||HK|Hybrid|RT||... at 85%

>Cluster 334

0 648aa, >LILAB_07470||HK|Classic|T|Orphan|... at 90%

1 689aa, >MXAN_0245||HK|Classic|T|Orphan|... *

2 647aa, >A176_5388||HK|Classic|T||... at 88%

>Cluster 335

0 685aa, >A2cp1_2920||HK|CheA|T|Orphan|... at 93%

1 687aa, >Adeh_2736||HK|CheA|T|Orphan|... *

2 685aa, >AnaeK_2828||HK|CheA|T|Orphan|... at 93%

>Cluster 336

0 673aa, >MXAN_7362||RR|PleD|RRRH|Complex|... at 91%

1 673aa, >COCOR_07948||RR|PleD|RHRR|Complex|... at 70%

2 674aa, >A176_3694||RR|PleD|R||... *

>Cluster 337

0 661aa, >A2cp1_2574||HK|CheA|T|Pair|... *

1 658aa, >Adeh_1379||HK|CheA|T|Pair|... at 91%

2 660aa, >AnaeK_2478||HK|CheA|T|Pair|... at 97%

>Cluster 338

0 630aa, >Adeh_1277||HK|Classic|T|Pair|... at 94%

1 654aa, >AnaeK_2584||HK|Classic|T|Pair|... *

2 626aa, >Anae109_2491||HK|Classic|T|Pair|... at 72%

>Cluster 339

0 653aa, >sce0794||RR|CyC-C|R|Pair|... *

1 650aa, >sce5834||RR|CyC-C|R|Orphan|... at 61%

2 650aa, >sce8885||RR|CyC-C|R|Pair|... at 80%

>Cluster 340&

0 514aa, >LILAB_07860||HK|Classic|T|Orphan|... at 84%

1 642aa, >MXAN_0176||HK|Classic|T|Orphan|... *

2 520aa, >A176_3132||HK|Classic|T||... at 75%

>Cluster 341

0 633aa, >A2cp1_1737||HK|Hybrid|TR|Pair|... *

1 633aa, >Adeh_2210||HK|Hybrid|TR|Pair|... at 92%

2 633aa, >AnaeK_1664||HK|Hybrid|TR|Pair|... at 98%

>Cluster 342

0 595aa, >A2cp1_2150||HK|Classic|T|Orphan|... at 89%

1 621aa, >Adeh_1782||HK|Classic|T|Orphan|... *

2 618aa, >AnaeK_2058||HK|Classic|T|Orphan|... at 88%

>Cluster 343

0 611aa, >A2cp1_3869||HK|Classic|T|Pair|... at 96%

1 613aa, >Adeh_3728||HK|Classic|T|Pair|... *

2 611aa, >AnaeK_3785||HK|Classic|T|Pair|... at 95%

>Cluster 344

0 591aa, >A2cp1_2759||HK|Classic|T|Orphan|... at 91%

1 609aa, >Adeh_1206||HK|Classic|T|Orphan|... *

2 590aa, >AnaeK_2664||HK|Classic|T|Orphan|... at 91%

>Cluster 345

0 589aa, >LILAB_11765||HK|Classic|T|Orphan|... at 67%

1 574aa, >MXAN_6941||HK|Classic|T|Orphan|... at 67%

2 604aa, >A176_5460||HK|Classic|T||... *

>Cluster 346

0 601aa, >A2cp1_1239||HK|Classic|T|Orphan|... *

1 463aa, >Adeh_1111||HK|Classic|T|Orphan|... at 95%

2 601aa, >AnaeK_1171||HK|Classic|T|Orphan|... at 98%

>Cluster 347

0 594aa, >A2cp1_2171||HK|Classic|T|Pair|... *

1 594aa, >Adeh_1763||HK|Classic|T|Pair|... at 92%

2 594aa, >AnaeK_2077||HK|Classic|T|Pair|... at 94%

>Cluster 348

0 526aa, >LILAB_34920||HK|Classic|T|Orphan|... at 87%

1 578aa, >MXAN_5483||HK|Classic|T|Orphan|... *

2 553aa, >A176_7617||HK|Classic|T||... at 82%

>Cluster 349$

0 576aa, >LILAB_28370||HK|CheA|T|Pair|... *

1 572aa, >COCOR_05511||HK|CheA|T|Orphan|... at 72%

2 558aa, >Anae109_0647||HK|CheA|T|Orphan|... at 50%

>Cluster 350

0 515aa, >LILAB_05590||HK|Classic|T|Orphan|... at 88%

1 569aa, >MXAN_0643||HK|Classic|T|Orphan|... *

2 515aa, >A176_1266||HK|Classic|T||... at 85%

>Cluster 351^

0 552aa, >LILAB_27495||HK|Classic|T|Orphan|... at 65%

1 551aa, >COCOR_01905||HK|Classic|T|Orphan|... at 66%

2 563aa, >COCOR_04361||HK|Classic|T|Orphan|... *

>Cluster 352

0 524aa, >LILAB_09100||HK|Hybrid|RT|Pair|... at 90%

1 551aa, >MXAN_7444||HK|Hybrid|RT|Pair|... *

2 514aa, >A176_3768||HK|Hybrid|RT||... at 89%

>Cluster 353

0 547aa, >A2cp1_0036||HK|Classic|T|Orphan|... *

1 469aa, >Adeh_0021||HK|Classic|T|Orphan|... at 81%

2 465aa, >AnaeK_0023||HK|Classic|T|Orphan|... at 96%

>Cluster 354

0 546aa, >LILAB_28980||HK|Classic|T|Pair|... at 86%

1 526aa, >MXAN_4165||HK|Classic|T|Pair|... at 85%

2 547aa, >A176_4024||HK|Classic|T||... *

>Cluster 355

0 539aa, >LILAB_09570||HK|Classic|T|Complex|... at 84%

1 543aa, >MXAN_7368||HK|Classic|T|Orphan|... *

2 540aa, >A176_3699||HK|Classic|T||... at 83%

>Cluster 356

0 535aa, >LILAB_32870||HK|Classic|T|Orphan|... *

1 535aa, >MXAN_4988||HK|Classic|T|Orphan|... at 90%

2 535aa, >A176_6634||HK|Classic|T||... at 82%

>Cluster 357

0 473aa, >LILAB_10780||HK|Classic|T|Orphan|... at 86%

1 533aa, >MXAN_7123||HK|Classic|T|Orphan|... *

2 474aa, >A176_5659||HK|Classic|T||... at 81%

>Cluster 358£

0 530aa, >A2cp1_0943||HK|Classic|T|Pair|... *

1 530aa, >AnaeK_0940||HK|Classic|T|Pair|... at 96%

2 522aa, >Anae109_3103||HK|Classic|T|Complex|... at 61%

>Cluster 359

0 527aa, >A2cp1_1579||RR|unclassified|R|Orphan|... at 91%

1 528aa, >Adeh_2380||RR|unclassified|R|Orphan|... *

2 527aa, >AnaeK_1484||RR|unclassified|R|Orphan|... at 91%

>Cluster 360

0 522aa, >A2cp1_2392||HK|Classic|T|Orphan|... at 99%

1 523aa, >Adeh_1560||HK|Classic|T|Orphan|... at 95%

2 524aa, >AnaeK_2304||HK|Classic|T|Orphan|... *

>Cluster 361

0 521aa, >LILAB_21570||HK|Hybrid|TR|Pair|... *

1 521aa, >MXAN_2763||HK|Hybrid|TR|Pair|... at 89%

2 521aa, >A176_0211||HK|Hybrid|TR||... at 85%

>Cluster 362

0 512aa, >A2cp1_3135||HK|Hybrid|TR|Complex|... *

1 512aa, >Adeh_2943||HK|Hybrid|TR|Complex|... at 83%

2 512aa, >AnaeK_3029||HK|Hybrid|TR|Complex|... at 91%

>Cluster 363

0 507aa, >Adeh_0367||HK|Classic|T|Orphan|... at 81%

1 509aa, >AnaeK_0395||HK|Classic|T|Orphan|... *

2 403aa, >Anae109_4211||HK|Classic|T|Orphan|... at 53%

>Cluster 364

0 502aa, >LILAB_30885||HK|Classic|T|Pair|... at 56%

1 508aa, >STAUR_5453||HK|Classic|T|Pair|... *

2 477aa, >A176_1900||HK|Classic|T||... at 54%

>Cluster 365

0 502aa, >LILAB_26590||HK|Classic|T|Pair|... *

1 500aa, >COCOR_04327||HK|Classic|T|Pair|... at 62%

2 482aa, >A176_6203||HK|Classic|T||... at 73%

>Cluster 366

0 501aa, >LILAB_09120||HK|Classic|T|Pair|... *

1 501aa, >MXAN_7439||HK|Classic|T|Pair|... at 89%

2 500aa, >A176_3763||HK|Classic|T||... at 86%

>Cluster 367

0 492aa, >LILAB_11250||HK|Classic|T|Orphan|... *

1 492aa, >MXAN_7027||HK|Classic|T|Orphan|... at 86%

2 470aa, >A176_5558||HK|Classic|T||... at 79%

>Cluster 368

0 484aa, >A2cp1_3870||RR|NtrC|R|Pair|... *

1 484aa, >Adeh_3729||RR|NtrC|R|Pair|... at 95%

2 484aa, >AnaeK_3786||RR|NtrC|R|Pair|... at 99%

>Cluster 369&

0 325aa, >LILAB_00195||HK|Classic|T|Orphan|... at 87%

1 472aa, >COCOR_06745||HK|Classic|T|Orphan|... *

2 419aa, >STAUR_2397||HK|Classic|T|Orphan|... at 86%

>Cluster 370

0 472aa, >A2cp1_0108||HK|Classic|T|Orphan|... *

1 472aa, >Adeh_0090||HK|Classic|T|Orphan|... at 88%

2 472aa, >AnaeK_0097||HK|Classic|T|Orphan|... at 98%

>Cluster 371

0 451aa, >A2cp1_2498||HK|Classic|T|Complex|... at 94%

1 471aa, >Adeh_1451||HK|Classic|T|Pair|... *

2 451aa, >AnaeK_2411||HK|Classic|T|Pair|... at 94%

>Cluster 372

0 459aa, >A2cp1_3996||HK|Classic|T|Pair|... at 96%

1 470aa, >Adeh_3855||HK|Classic|T|Pair|... *

2 460aa, >AnaeK_3918||HK|Classic|T|Pair|... at 89%

>Cluster 373

0 464aa, >LILAB_16305||HK|Classic|T|Pair|... *

1 463aa, >MXAN_5996||HK|Classic|T|Pair|... at 87%

2 463aa, >A176_1083||HK|Classic|T||... at 86%

>Cluster 374

0 439aa, >LILAB_05675||HK|Classic|T|Orphan|... at 52%

1 464aa, >COCOR_00539||HK|Classic|T|Orphan|... *

2 442aa, >A176_1281||HK|Classic|T||... at 51%

>Cluster 375

0 463aa, >LILAB_13020||HK|Classic|T|Orphan|... *

1 462aa, >MXAN_6688||HK|Classic|T|Orphan|... at 91%

2 463aa, >A176_2228||HK|Classic|T||... at 87%

>Cluster 376£

0 460aa, >Adeh_1992||RR|NtrC|R|Pair|... at 98%

1 461aa, >AnaeK_1886||RR|NtrC|R|Complex|... *

2 459aa, >Anae109_1847||RR|NtrC|R|Complex|... at 83%

>Cluster 377

0 459aa, >LILAB_15320||HK|Classic|T|Pair|... at 82%

1 427aa, >MXAN_6223||HK|Classic|T|Pair|... at 86%

2 460aa, >A176_2288||HK|Classic|T||... *

>Cluster 378

0 446aa, >LILAB_25015||HK|Classic|T|Pair|... at 87%

1 452aa, >MXAN_3451||HK|Classic|T|Pair|... at 88%

2 453aa, >A176_5032||HK|Classic|T||... *

>Cluster 379

0 449aa, >Adeh_1276||RR|NtrC|R|Pair|... at 80%

1 449aa, >AnaeK_2585||RR|NtrC|R|Pair|... at 81%

2 452aa, >Anae109_2492||RR|NtrC|R|Pair|... *

>Cluster 380

0 451aa, >LILAB_09115||RR|NtrC|R|Pair|... *

1 451aa, >MXAN_7440|Nla24|RR|NtrC|R|Pair|... at 96%

2 451aa, >A176_3764||RR|NtrC|R||... at 94%

>Cluster 381

0 431aa, >LILAB_29150||HK|Classic|T|Pair|... at 83%

1 444aa, >MXAN_4203||HK|Classic|T|Pair|... *

2 439aa, >A176_7236||HK|Classic|T||... at 62%

>Cluster 382

0 439aa, >LILAB_16335||HK|Classic|T|Pair|... at 80%

1 444aa, >MXAN_5990||HK|Classic|T|Pair|... *

2 431aa, >A176_1076||HK|Classic|T||... at 74%

>Cluster 383

0 413aa, >A2cp1_1044||HK|Classic|T|Pair|... at 82%

1 444aa, >Adeh_0983||HK|Classic|T|Pair|... *

2 421aa, >Anae109_3632||HK|Classic|T|Pair|... at 63%

>Cluster 384

0 431aa, >A2cp1_2775||HK|Classic|T|Complex|... *

1 431aa, >Adeh_1191||HK|Classic|T|Complex|... at 92%

2 431aa, >AnaeK_2680||HK|Classic|T|Complex|... at 99%

>Cluster 385

0 408aa, >LILAB_29115||HK|Classic|T|Pair|... at 89%

1 429aa, >MXAN_4197||HK|Classic|T|Pair|... *

2 413aa, >A176_4059||HK|Classic|T||... at 84%

>Cluster 386

0 394aa, >LILAB_11435||HK|Classic|T|Complex|... at 80%

1 419aa, >MXAN_6994||HK|Classic|T|Complex|... *

2 401aa, >A176_5517||HK|Classic|T||... at 70%

>Cluster 387

0 386aa, >LILAB_11550||HK|Classic|T|Orphan|... at 94%

1 411aa, >MXAN_6971||HK|Classic|T|Orphan|... *

2 386aa, >A176_5489||HK|Classic|T||... at 92%

>Cluster 388

0 388aa, >MXAN_1679||HK|Classic|T|Pair|... at 62%

1 405aa, >COCOR_04590||HK|Classic|T|Pair|... *

2 394aa, >A176_0092||HK|Classic|T||... at 60%

>Cluster 389

0 398aa, >MXAN_5082||HK|Classic|T|Pair|... *

1 371aa, >COCOR_02407||HK|Classic|T|Pair|... at 62%

2 373aa, >A176_6543||HK|Classic|T||... at 76%

>Cluster 390

0 384aa, >A2cp1_0195||HK|Classic|T|Orphan|... at 92%

1 389aa, >Adeh_0175||HK|Classic|T|Orphan|... *

2 383aa, >AnaeK_0184||HK|Classic|T|Orphan|... at 91%

>Cluster 391

0 385aa, >A2cp1_2585||RR|FrzZ|RR|Complex|... at 97%

1 386aa, >Adeh_1368||RR|FrzZ|RR|Complex|... *

2 385aa, >AnaeK_2489||RR|FrzZ|RR|Complex|... at 96%

>Cluster 392

0 383aa, >A2cp1_0535||HK|Classic|T|Complex|... *

1 383aa, >Adeh_0506||HK|Classic|T|Complex|... at 95%

2 383aa, >AnaeK_0539||HK|Classic|T|Complex|... at 99%

>Cluster 393

0 373aa, >LILAB_04815||HK|Hybrid|RT|Complex|... at 79%

1 378aa, >STAUR_7449||HK|Hybrid|RT|Complex|... *

2 375aa, >A176_4334||HK|Hybrid|RT||... at 77%

>Cluster 394

0 371aa, >COCOR_05434||HK|Hybrid|RT|Pair|... at 76%

1 373aa, >STAUR_3022||HK|Hybrid|RT|Pair|... *

2 372aa, >A176_6691||HK|Hybrid|RT||... at 81%

>Cluster 395

0 363aa, >A2cp1_4405||HK|Classic|T|Pair|... *

1 363aa, >AnaeK_4382||HK|Classic|T|Pair|... at 99%

2 351aa, >Anae109_4398||HK|Classic|T|Pair|... at 74%

>Cluster 396

0 348aa, >LILAB_06795||HK|Classic|T|Pair|... at 74%

1 345aa, >COCOR_05884||HK|Classic|T|Pair|... at 56%

2 351aa, >A176_1532||HK|Classic|T||... *

>Cluster 397

0 350aa, >A2cp1_2914||RR|CheB|R|Complex|... *

1 350aa, >Adeh_2730||RR|CheB|R|Complex|... at 92%

2 350aa, >AnaeK_2822||RR|CheB|R|Complex|... at 98%

>Cluster 398

0 332aa, >LILAB_21130||PP|HisKa|H|Complex|... *

1 290aa, >MXAN_2687||PP|HisKa|H|Complex|... at 82%

2 240aa, >A176_0129||PP|HisKa|H||... at 83%

>Cluster 399^

0 237aa, >A2cp1_0565||HK|Classic|T|Orphan|... at 59%

1 322aa, >Anae109_1027||HK|Classic|T|Orphan|... *

2 233aa, >Anae109_2976||HK|Classic|T|Orphan|... at 68%

>Cluster 400

0 256aa, >MXAN_5083||RR|LytTR|R|Pair|... *

1 256aa, >COCOR_02406||RR|LytTR|R|Pair|... at 74%

2 256aa, >A176_6542||RR|LytTR|R||... at 85%

>Cluster 401

0 242aa, >LILAB_06390||HK|Classic|T|Complex|... *

1 242aa, >MXAN_0461|RedE|HK|Classic|T|Complex|... at 92%

2 242aa, >A176_1435||HK|Classic|T||... at 87%

>Cluster 402

0 229aa, >LILAB_11370||RR|unclassified|R|Complex|... at 55%

1 242aa, >COCOR_00393||RR|CheY|R|Complex|... *

2 229aa, >A176_5532||RR|CheY|R||... at 57%

>Cluster 403

0 240aa, >LILAB_06800||RR|LytTR|R|Pair|... at 90%

1 236aa, >COCOR_05885||RR|LytTR|R|Pair|... at 84%

2 242aa, >A176_1533||RR|LytTR|R||... *

>Cluster 404£

0 239aa, >A2cp1_1973||HK|Classic|T|Pair|... *

1 239aa, >AnaeK_1888||HK|Classic|T|Complex|... at 97%

2 238aa, >Anae109_1849||PP|HisKa|H|Complex|... at 58%

>Cluster 405

0 231aa, >LILAB_35015||RR|unclassified|R|Orphan|... *

1 231aa, >MXAN_5505||RR|unclassified|R|Orphan|... at 94%

2 231aa, >A176_7641||RR|CheY|R||... at 87%

>Cluster 406

0 224aa, >MXAN_1680||RR|OmpR|R|Pair|... *

1 222aa, >COCOR_04589||RR|OmpR|R|Pair|... at 77%

2 224aa, >A176_0091||RR|OmpR|R||... at 88%

>Cluster 407

0 210aa, >A2cp1_2172||RR|NarL|R|Pair|... *

1 210aa, >Adeh_1762||RR|NarL|R|Pair|... at 94%

2 210aa, >AnaeK_2078||RR|NarL|R|Pair|... at 94%

>Cluster 408

0 199aa, >PPSIR1_00650||RR|Xre|R||... *

1 141aa, >PPSIR1_01292||RR|Xre|R||... at 82%

2 198aa, >Hoch_5158||RR|Xre|R|Orphan|... at 61%

>Cluster 409

0 176aa, >LILAB_29145||RR|unclassified|R|Pair|... at 75%

1 187aa, >MXAN_4202||RR|unclassified|R|Pair|... at 71%

2 192aa, >A176_7237||RR|CheY|R||... *

>Cluster 410

0 165aa, >A2cp1_0033||RR|CheY|R|Orphan|... at 73%

1 163aa, >Adeh_0017||RR|CheY|R|Orphan|... at 77%

2 174aa, >AnaeK_0019||RR|CheY|R|Orphan|... *

>Cluster 411

0 142aa, >LILAB_11845||RR|CheY|R|Orphan|... at 85%

1 164aa, >MXAN_6926||RR|CheY|R|Orphan|... *

2 142aa, >A176_5441||RR|CheY|R||... at 85%

>Cluster 412

0 117aa, >LILAB_15745||RR|CheY|R|Orphan|... at 88%

1 150aa, >MXAN_6104||RR|CheY|R|Orphan|... *

2 117aa, >A176_3441||RR|CheY|R||... at 84%

>Cluster 413

0 143aa, >COCOR_04615||RR|CheY|R|Complex|... *

1 140aa, >sce7813||RR|CheY|R|Orphan|... at 72%

2 138aa, >sce8714||RR|CheY|R|Complex|... at 68%

>Cluster 414

0 138aa, >A2cp1_1045||RR|CheY|R|Pair|... at 81%

1 139aa, >Adeh_0984||RR|CheY|R|Pair|... *

2 127aa, >Anae109_3631||RR|CheY|R|Pair|... at 66%

>Cluster 415

0 138aa, >A2cp1_3195||RR|CheY|R|Orphan|... *

1 135aa, >Adeh_2995||RR|CheY|R|Orphan|... at 89%

2 138aa, >AnaeK_3094||RR|CheY|R|Orphan|... at 98%

>Cluster 416

0 138aa, >A2cp1_4036||RR|CheY|R|Orphan|... *

1 138aa, >Adeh_3891||RR|CheY|R|Orphan|... at 99%

2 138aa, >AnaeK_3995||RR|CheY|R|Orphan|... at 94%

>Cluster 417

0 134aa, >LILAB_11220||RR|CheY|R|Orphan|... *

1 134aa, >MXAN_7033||RR|CheY|R|Orphan|... at 93%

2 133aa, >A176_5565||RR|CheY|R||... at 78%

>Cluster 418

0 132aa, >A2cp1_1413||RR|CheY|R|Orphan|... *

1 132aa, >Adeh_2538||RR|CheY|R|Orphan|... at 96%

2 132aa, >AnaeK_1311||RR|CheY|R|Orphan|... at 98%

>Cluster 419

0 115aa, >A2cp1_3674||RR|CheY|R|Orphan|... at 92%

1 129aa, >Adeh_3518||RR|CheY|R|Orphan|... *

2 115aa, >AnaeK_3609||RR|CheY|R|Orphan|... at 93%

>Cluster 420

0 128aa, >LILAB_11625||RR|CheY|R|Complex|... *

1 128aa, >MXAN_6956|DotR|RR|CheY|R|Complex|... at 92%

2 128aa, >A176_5472||RR|CheY|R||... at 85%

>Cluster 421

0 124aa, >A2cp1_1356||RR|CheY|R|Orphan|... at 92%

1 126aa, >Adeh_2593||RR|CheY|R|Orphan|... *

2 124aa, >AnaeK_1255||RR|CheY|R|Orphan|... at 91%

>Cluster 422$

0 125aa, >COCOR_07542||RR|CheY|R|Complex|... *

1 122aa, >STAUR_0302||RR|CheY|R|Pair|... at 50%

2 119aa, >A176_2140||RR|CheY|R||... at 68%

>Cluster 423

0 122aa, >A2cp1_2575||RR|CheY|R|Pair|... *

1 122aa, >Adeh_1378||RR|CheY|R|Pair|... at 96%

2 122aa, >AnaeK_2479||RR|CheY|R|Pair|... at 99%

>Cluster 424

0 2003aa, >sce3989||HK|Hybrid|TR|Complex|... at 67%

1 2073aa, >sce6827||HK|Hybrid|TR|Complex|... *

>Cluster 425

0 1833aa, >LILAB_01590||RR|unclassified|R|Orphan|... at 67%

1 1850aa, >MXAN_1429||RR|unclassified|R|Orphan|... *

>Cluster 426

0 1798aa, >STAUR_7988||HK|Classic|T|Orphan|... at 54%

1 1816aa, >sce5838||HK|Classic|T|Orphan|... *

>Cluster 427^

0 1788aa, >STAUR_1273||HK|Classic|T|Orphan|... *

1 1787aa, >STAUR_4462||HK|Classic|T|Orphan|... at 61%

>Cluster 428

0 1676aa, >sce5107||HK|Hybrid|TRRR|Complex|... *

1 1444aa, >sce7807||HK|Hybrid|TRH|Complex|... at 51%

>Cluster 429

0 1652aa, >COCOR_06966||HK|Classic|T|Orphan|... *

1 1579aa, >STAUR_1458||HK|Classic|T|Orphan|... at 67%

>Cluster 430

0 1143aa, >sce0206||HK|Hybrid|TRT|Complex|... at 50%

1 1307aa, >sce0713||HK|Hybrid|TRT|Complex|... *

>Cluster 431&

0 1144aa, >COCOR_07711||HK|Hybrid|TRT|Complex|... *

1 972aa, >STAUR_3875||HK|Hybrid|TRT|Complex|... at 50%

>Cluster 432

0 1137aa, >COCOR_00081||HK|Hybrid|TRR|Complex|... *

1 1126aa, >STAUR_7016||HK|Hybrid|TRR|Complex|... at 62%

>Cluster 433

0 1117aa, >LILAB_09565||HK|Hybrid|TRT|Complex|... at 60%

1 1124aa, >COCOR_03762||HK|Hybrid|TRT|Complex|... *

>Cluster 434

0 1000aa, >COCOR_05180||HK|Hybrid|TRT|Complex|... *

1 975aa, >STAUR_1171||HK|Hybrid|TRT|Complex|... at 61%

>Cluster 435

0 459aa, >Anae109_2452||HK|Hybrid|TR|Pair|... at 50%

1 961aa, >sce4365||HK|Hybrid|TR|Pair|... *

>Cluster 436

0 902aa, >Adeh_0892||HK|Classic|T|Pair|... at 51%

1 910aa, >sce1355||HK|Classic|T|Pair|... *

>Cluster 437

0 250aa, >COCOR_04616||HK|Classic|T|Complex|... at 53%

1 883aa, >sce5100||HK|Hybrid|RT|Pair|... *

>Cluster 438

0 815aa, >LILAB_06940||HK|Classic|T|Orphan|... at 82%

1 869aa, >MXAN_0347||HK|Classic|T|Orphan|... *

>Cluster 439

0 865aa, >COCOR_00375||HK|Classic|T|Orphan|... *

1 736aa, >A176_5561||HK|Classic|T||... at 50%

>Cluster 440

0 844aa, >A2cp1_1756||HK|Hybrid|TR|Pair|... *

1 844aa, >AnaeK_1685||HK|Hybrid|TR|Pair|... at 97%

>Cluster 441$^

0 781aa, >STAUR_0493||HK|Hybrid|TR|Complex|... at 53%

1 798aa, >STAUR_2886||HK|Hybrid|TR|Pair|... *

>Cluster 442^

0 664aa, >Anae109_1040||HK|Hybrid|TR|Pair|... at 51%

1 764aa, >Anae109_2356||HK|Hybrid|TR|Pair|... *

>Cluster 443

0 761aa, >A2cp1_2484||HK|Hybrid|TR|Complex|... *

1 761aa, >AnaeK_2396||HK|Hybrid|TR|Complex|... at 97%

>Cluster 444

0 749aa, >COCOR_02638||HK|CheA|T|Pair|... *

1 653aa, >STAUR_5607||HK|CheA|T|Pair|... at 70%

>Cluster 445

0 724aa, >LILAB_23455||HK|Hybrid|TR|Pair|... at 81%

1 737aa, >A176_6800||HK|Hybrid|TR||... *

>Cluster 446$

0 731aa, >COCOR_07249||HK|Classic|T|Orphan|... *

1 685aa, >STAUR_4907||HK|Classic|T|Complex|... at 57%

>Cluster 447

0 698aa, >COCOR_00527||HK|Classic|T|Orphan|... at 60%

1 723aa, >A176_1304||HK|Classic|T||... *

>Cluster 448

0 657aa, >COCOR_07649||HK|Classic|T|Orphan|... at 50%

1 700aa, >A176_3662||HK|Classic|T||... *

>Cluster 449&

0 570aa, >COCOR_03444||HK|Classic|T|Orphan|... at 64%

1 698aa, >STAUR_3203||HK|Classic|T|Orphan|... *

>Cluster 450

0 647aa, >COCOR_00552||HK|Classic|T|Orphan|... at 55%

1 693aa, >STAUR_7668||HK|Classic|T|Orphan|... *

>Cluster 451

0 685aa, >LILAB_11585||HK|CheA|T|Complex|... at 51%

1 688aa, >sce2657||HK|CheA|T|Complex|... *

>Cluster 452

0 625aa, >A2cp1_1219||RR|unclassified|R|Orphan|... at 98%

1 626aa, >AnaeK_1151||RR|unclassified|R|Orphan|... *

>Cluster 453

0 583aa, >LILAB_34530||RR|PleD|R|Orphan|... at 63%

1 600aa, >A176_7518||RR|PleD|R||... *

>Cluster 454

0 597aa, >COCOR_04626||HK|Classic|T|Orphan|... *

1 537aa, >sce0793||HK|Classic|T|Pair|... at 58%

>Cluster 455

0 562aa, >sce1226||HK|CheA|T|Orphan|... at 61%

1 572aa, >sce2203||HK|CheA|T|Orphan|... *

>Cluster 456$&

0 417aa, >COCOR_00607||HK|Classic|T|Pair|... at 50%

1 555aa, >STAUR_7563||HK|Hybrid|TR|Complex|... *

>Cluster 457

0 545aa, >LILAB_07360||HK|Hybrid|RT|Complex|... at 60%

1 554aa, >COCOR_07786||HK|Hybrid|RT|Complex|... *

>Cluster 458£

0 542aa, >A2cp1_3376||HK|Classic|T|Complex|... *

1 408aa, >Adeh_3180||HK|Classic|T|Orphan|... at 93%

>Cluster 459

0 503aa, >sce3265||HK|Classic|T|Pair|... at 52%

1 539aa, >sce7809||HK|Classic|T|Complex|... *

>Cluster 460

0 538aa, >PPSIR1_40075||HK|Hybrid|TR||... *

1 512aa, >PPSIR1_40430||HK|Hybrid|TR||... at 50%

>Cluster 461

0 531aa, >MXAN_0706||HK|Classic|T|Orphan|... *

1 514aa, >A176_7419||HK|Classic|T||... at 72%

>Cluster 462

0 527aa, >COCOR_00133||HK|Hybrid|RT|Complex|... *

1 504aa, >STAUR_0462||HK|Hybrid|RT|Complex|... at 65%

>Cluster 463

0 513aa, >PPSIR1_29598||RR|NtrC|R||... *

1 481aa, >sce2560||RR|NtrC|R|Orphan|... at 51%

>Cluster 464

0 487aa, >PPSIR1_29031||RR|NtrC|R||... at 52%

1 503aa, >sce3597||RR|NtrC|R|Complex|... *

>Cluster 465

0 478aa, >COCOR_07820||HK|Classic|T|Pair|... *

1 478aa, >STAUR_7043||HK|Classic|T|Pair|... at 53%

>Cluster 466

0 470aa, >A2cp1_3328||RR|unclassified|R|Orphan|... *

1 470aa, >AnaeK_3241||RR|unclassified|R|Orphan|... at 97%

>Cluster 467

0 458aa, >LILAB_21150||HK|Hybrid|TR|Pair|... *

1 328aa, >A176_0134||HK|Classic|T||... at 59%

>Cluster 468^

0 454aa, >STAUR_1775||HK|Hybrid|RT|Pair|... *

1 442aa, >STAUR_5256||HK|Hybrid|RT|Pair|... at 55%

>Cluster 469

0 454aa, >A2cp1_2095||HK|Classic|T|Orphan|... *

1 454aa, >AnaeK_2010||HK|Classic|T|Orphan|... at 97%

>Cluster 470

0 451aa, >MXAN_3812||HK|Classic|T|Pair|... *

1 445aa, >A176_5279||HK|Classic|T||... at 75%

>Cluster 471$^

0 440aa, >STAUR_1837||HK|Classic|T|Orphan|... at 51%

1 441aa, >STAUR_4550||HK|Classic|T|Pair|... *

>Cluster 472

0 435aa, >COCOR_07567||HK|Classic|T|Pair|... at 50%

1 439aa, >STAUR_1186||HK|Classic|T|Pair|... *

>Cluster 473

0 436aa, >Adeh_3134||RR|unclassified|R|Orphan|... *

1 408aa, >AnaeK_1417||RR|unclassified|R|Orphan|... at 64%

>Cluster 474

0 405aa, >COCOR_00331||HK|Classic|T|Orphan|... at 66%

1 411aa, >A176_2319||HK|Classic|T||... *

>Cluster 475

0 401aa, >Adeh_3532||HK|Classic|T|Orphan|... *

1 401aa, >AnaeK_3620||HK|Classic|T|Orphan|... at 90%

>Cluster 476

0 387aa, >COCOR_03127||HK|Classic|T|Pair|... *

1 360aa, >STAUR_6622||HK|Classic|T|Pair|... at 55%

>Cluster 477^

0 355aa, >Anae109_0470||RR|CheB|R|Complex|... at 63%

1 370aa, >Anae109_3532||RR|CheB|R|Complex|... *

>Cluster 478$

0 354aa, >COCOR_04887||RR|CheB|R|Orphan|... at 54%

1 364aa, >STAUR_5660||RR|CheB|R|Pair|... *

>Cluster 479

0 349aa, >STAUR_0490||RR|CheB|R|Complex|... at 51%

1 352aa, >Anae109_2366||RR|CheB|R|Complex|... *

>Cluster 480$

0 275aa, >LILAB_34980||RR|unclassified|R|Orphan|... at 65%

1 294aa, >STAUR_4548||RR|unclassified|R|Pair|... *

>Cluster 481

0 235aa, >Hoch_4665||RR|OmpR|R|Complex|... at 57%

1 270aa, >sce8615||RR|OmpR|R|Orphan|... *

>Cluster 482

0 256aa, >sce5166||RR|FrzZ|RR|Complex|... *

1 244aa, >sce8799||RR|FrzZ|RR|Complex|... at 66%

>Cluster 483

0 243aa, >sce5168||HK|Classic|T|Complex|... at 55%

1 244aa, >sce8797||HK|Classic|T|Complex|... *

>Cluster 484

0 238aa, >STAUR_1948||HK|Classic|T|Orphan|... *

1 223aa, >A176_0197||HK|Classic|T||... at 59%

>Cluster 485

0 220aa, >PPSIR1_37479||RR|NarL|R||... at 66%

1 237aa, >PPSIR1_40730||RR|NarL|R||... *

>Cluster 486

0 229aa, >PPSIR1_03308||RR|OmpR|R||... at 51%

1 231aa, >PPSIR1_20099||RR|OmpR|R||... *

>Cluster 487

0 224aa, >A176_7251||RR|OmpR|R||... *

1 224aa, >A176_1830||RR|OmpR|R||... at 89%

>Cluster 488

0 215aa, >COCOR_03128||RR|NarL|R|Pair|... *

1 213aa, >STAUR_6623||RR|NarL|R|Pair|... at 78%

>Cluster 489

0 154aa, >COCOR_00132||RR|CheY|R|Complex|... *

1 152aa, >STAUR_0461||RR|CheY|R|Complex|... at 80%

>Cluster 490

0 140aa, >A2cp1_1515||RR|CheY|R|Pair|... *

1 130aa, >Adeh_2443||RR|CheY|R|Pair|... at 90%

>Cluster 491

0 136aa, >LILAB_29460||RR|CheY|R|Complex|... at 86%

1 137aa, >MXAN_4260||RR|CheY|R|Complex|... *

>Cluster 492

0 132aa, >Adeh_1968||RR|PrrA|R|Orphan|... *

1 129aa, >AnaeK_1910||RR|PrrA|R|Orphan|... at 93%

>Cluster 493

0 123aa, >sce1728||RR|CheY|R|Complex|... at 56%

1 126aa, >sce2654||RR|CheY|R|Complex|... *

>Cluster 494

0 111aa, >PPSIR1_09006||RR|CheY|R||... at 70%

1 125aa, >sce1763||RR|CheY|R|Orphan|... *

>Cluster 495

0 109aa, >PPSIR1_02698||RR|CheY|R||... at 59%

1 124aa, >PPSIR1_27178||RR|CheY|R||... *

>Cluster 496^

0 122aa, >STAUR_4571||RR|CheY|R|Complex|... at 53%

1 123aa, >STAUR_4572||RR|CheY|R|Complex|... *

>Cluster 497

0 121aa, >LILAB_10485||RR|CheY|R|Pair|... *

1 121aa, >MXAN_7178||RR|CheY|R|Pair|... at 90%

>Cluster 498

0 2543aa, >Hoch_5788||HK|Classic|T|Orphan|... *

>Cluster 499

0 2468aa, >Hoch_3278||HK|Hybrid|TR|Pair|... *

>Cluster 500

0 2031aa, >Hoch_3427||HK|Hybrid|TR|Pair|... *

>Cluster 501

0 2015aa, >COCOR_01331||RR|CheY|R|Orphan|... *

>Cluster 502

0 1984aa, >sce1494||HK|Classic|T|Orphan|... *

>Cluster 503

0 1971aa, >PPSIR1_38666||HK|Classic|T||... *

>Cluster 504

0 1939aa, >PPSIR1_23409||HK|Hybrid|TR||... *

>Cluster 505

0 1928aa, >PPSIR1_07113||HK|Classic|T||... *

>Cluster 506

0 1922aa, >Hoch_2181||HK|Hybrid|TR|Pair|... *

>Cluster 507

0 1920aa, >PPSIR1_30175||HK|Classic|T||... *

>Cluster 508

0 1909aa, >PPSIR1_27003||HK|Classic|T||... *

>Cluster 509

0 1909aa, >PPSIR1_28333||HK|Classic|T||... *

>Cluster 510

0 1908aa, >PPSIR1_03133||HK|Classic|T||... *

>Cluster 511

0 1901aa, >PPSIR1_27198||HK|Classic|T||... *

>Cluster 512

0 1898aa, >PPSIR1_02606||HK|Classic|T||... *

>Cluster 513

0 1896aa, >PPSIR1_41259||HK|Classic|T||... *

>Cluster 514

0 1891aa, >PPSIR1_13033||HK|Classic|T||... *

>Cluster 515

0 1889aa, >PPSIR1_04348||HK|Classic|T||... *

>Cluster 516

0 1888aa, >PPSIR1_38861||HK|Classic|T||... *

>Cluster 517

0 1885aa, >Hoch_6208||HK|Hybrid|TR|Pair|... *

>Cluster 518

0 1885aa, >Hoch_6696||HK|Classic|T|Complex|... *

>Cluster 519

0 1874aa, >PPSIR1_33174||HK|Classic|T||... *

>Cluster 520

0 1873aa, >PPSIR1_14950||HK|Classic|T||... *

>Cluster 521

0 1850aa, >sce1859||HK|Classic|T|Orphan|... *

>Cluster 522

0 1839aa, >PPSIR1_20654||HK|Classic|T||... *

>Cluster 523

0 1838aa, >sce7702||RR|CheY|R|Orphan|... *

>Cluster 524

0 1832aa, >Hoch_1945||HK|Classic|T|Orphan|... *

>Cluster 525

0 1824aa, >PPSIR1_39520||HK|Classic|T||... *

>Cluster 526

0 1815aa, >STAUR_4560||HK|Classic|T|Orphan|... *

>Cluster 527

0 1813aa, >sce2676||HK|Classic|T|Complex|... *

>Cluster 528

0 1806aa, >sce7688||HK|Classic|T|Orphan|... *

>Cluster 529

0 1797aa, >Hoch_2277||HK|Classic|T|Complex|... *

>Cluster 530

0 1782aa, >sce8887||HK|Classic|T|Pair|... *

>Cluster 531

0 1781aa, >STAUR_0028||HK|Classic|T|Pair|... *

>Cluster 532

0 1780aa, >Hoch_4622||HK|Classic|T|Pair|... *

>Cluster 533

0 1769aa, >PPSIR1_41849||HK|Classic|T||... *

>Cluster 534

0 1759aa, >Hoch_1404||HK|Hybrid|TR|Pair|... *

>Cluster 535

0 1757aa, >STAUR_1364||HK|Classic|T|Orphan|... *

>Cluster 536

0 1716aa, >sce6844||HK|Classic|T|Orphan|... *

>Cluster 537

0 1698aa, >Hoch_5252||HK|Hybrid|TR|Pair|... *

>Cluster 538

0 1693aa, >sce1256||HK|Classic|T|Orphan|... *

>Cluster 539

0 1632aa, >STAUR_2229||RR|CheY|R|Pair|... *

>Cluster 540

0 1521aa, >PPSIR1_15240||HK|Hybrid|TR||... *

>Cluster 541

0 1453aa, >Hoch_4639||HK|Hybrid|TR|Pair|... *

>Cluster 542

0 1443aa, >STAUR_5119||RR|CheY|R|Orphan|... *

>Cluster 543

0 1420aa, >STAUR_7112||HK|Hybrid|TR|Complex|... *

>Cluster 544

0 1406aa, >Hoch_3141||HK|Hybrid|TRTR|Complex|... *

>Cluster 545

0 1360aa, >sce4190||HK|Hybrid|TR|Orphan|... *

>Cluster 546

0 1348aa, >Hoch_3250||HK|Hybrid|TR|Complex|... *

>Cluster 547

0 1311aa, >STAUR_7959||HK|Hybrid|TRTR|Complex|... *

>Cluster 548

0 1268aa, >COCOR_04260||HK|Hybrid|TRT|Complex|... *

>Cluster 549

0 1266aa, >COCOR_05524||HK|Hybrid|TRTR|Complex|... *

>Cluster 550

0 1244aa, >sce8150||RR|unclassified|HRR|Complex|... *

>Cluster 551

0 1212aa, >STAUR_7083||HK|Hybrid|TR|Pair|... *

>Cluster 552

0 1171aa, >sce3507||HK|Hybrid|TRTR|Complex|... *

>Cluster 553

0 1168aa, >COCOR_04660||HK|Classic|T|Pair|... *

>Cluster 554

0 1152aa, >sce0531||HK|Hybrid|TR|Orphan|... *

>Cluster 555

0 1142aa, >STAUR_0388||HK|Hybrid|TR|Pair|... *

>Cluster 556

0 1139aa, >COCOR_00131||HK|Hybrid|RT|Complex|... *

>Cluster 557

0 1131aa, >Hoch_0125||RR|unclassified|R|Orphan|... *

>Cluster 558

0 1131aa, >Hoch_3814||HK|Hybrid|TRR|Complex|... *

>Cluster 559

0 1122aa, >PPSIR1_35712||PP|HPt|H||... *

>Cluster 560

0 1111aa, >Anae109_1854||HK|Hybrid|RTRR|Complex|... *

>Cluster 561

0 1099aa, >sce5864||RR|unclassified|R|Orphan|... *

>Cluster 562

0 1088aa, >Adeh_3529||HK|Hybrid|TR|Pair|... *

>Cluster 563

0 1080aa, >MXAN_6150||HK|Classic|T|Pair|... *

>Cluster 564

0 1062aa, >STAUR_6076||HK|Unorthodox|TRRH|Complex|... *

>Cluster 565

0 1051aa, >sce2653||HK|Hybrid|TRT|Complex|... *

>Cluster 566

0 1029aa, >sce3649||RR|unclassified|R|Orphan|... *

>Cluster 567

0 1020aa, >STAUR_3168||HK|Hybrid|TRT|Complex|... *

>Cluster 568

0 1013aa, >PPSIR1_04388||HK|Classic|T||... *

>Cluster 569

0 1002aa, >STAUR_8145||HK|Hybrid|TRT|Complex|... *

>Cluster 570

0 1002aa, >sce2727||HK|Classic|T|Orphan|... *

>Cluster 571

0 998aa, >STAUR_7504||HK|Classic|T|Pair|... *

>Cluster 572

0 998aa, >Hoch_0467||RR|unclassified|R|Orphan|... *

>Cluster 573

0 991aa, >STAUR_7606||HK|Classic|T|Orphan|... *

>Cluster 574

0 990aa, >Hoch_5807||HK|Hybrid|TR|Pair|... *

>Cluster 575

0 984aa, >STAUR_1283||HK|Classic|T|Orphan|... *

>Cluster 576

0 979aa, >Anae109_2663||HK|Hybrid|TRT|Complex|... *

>Cluster 577

0 969aa, >Anae109_4250||HK|Classic|T|Complex|... *

>Cluster 578

0 965aa, >COCOR_07543||HK|Classic|T|Complex|... *

>Cluster 579

0 965aa, >Hoch_1794||HK|Unorthodox|TRH|Complex|... *

>Cluster 580

0 961aa, >A2cp1_4187||PP|HisKa|H|Orphan|... *

>Cluster 581

0 959aa, >COCOR_00594||HK|Classic|T|Orphan|... *

>Cluster 582

0 956aa, >STAUR_1397||HK|Hybrid|TR|Pair|... *

>Cluster 583

0 955aa, >sce5571||HK|Hybrid|TR|Complex|... *

>Cluster 584

0 951aa, >PPSIR1_24589||RR|CheY|R||... *

>Cluster 585

0 949aa, >sce1346||HK|Hybrid|TRRH|Complex|... *

>Cluster 586

0 947aa, >sce8715||HK|Hybrid|TR|Complex|... *

>Cluster 587

0 939aa, >sce1939||HK|Hybrid|TR|Complex|... *

>Cluster 588

0 931aa, >PPSIR1_32809||RR|unclassified|R||... *

>Cluster 589

0 929aa, >sce0801||HK|Classic|T|Pair|... *

>Cluster 590

0 928aa, >Hoch_2220||HK|Unorthodox|TRH|Complex|... *

>Cluster 591

0 927aa, >Anae109_2030||HK|Classic|T|Pair|... *

>Cluster 592

0 923aa, >Anae109_1771||HK|Hybrid|TR|Pair|... *

>Cluster 593

0 922aa, >STAUR_7809||HK|Classic|T|Orphan|... *

>Cluster 594

0 922aa, >sce7159||HK|Hybrid|TR|Pair|... *

>Cluster 595

0 920aa, >PPSIR1_08566||HK|Hybrid|TR||... *

>Cluster 596

0 919aa, >Hoch_2389||HK|Classic|T|Orphan|... *

>Cluster 597

0 919aa, >sce7776||HK|Hybrid|TRRH|Complex|... *

>Cluster 598

0 919aa, >sce8495||HK|Hybrid|TR|Pair|... *

>Cluster 599

0 918aa, >sce0624||HK|Hybrid|TR|Complex|... *

>Cluster 600

0 917aa, >Anae109_0027||HK|Hybrid|TR|Complex|... *

>Cluster 601

0 912aa, >Anae109_3912||HK|Classic|T|Orphan|... *

>Cluster 602

0 910aa, >Hoch_5342||RR|unclassified|R|Orphan|... *

>Cluster 603

0 906aa, >Hoch_6593||HK|Hybrid|TR|Pair|... *

>Cluster 604

0 899aa, >STAUR_4649||HK|Classic|T|Orphan|... *

>Cluster 605

0 885aa, >Hoch_5560||HK|Hybrid|TR|Complex|... *

>Cluster 606

0 885aa, >sce2655||HK|Hybrid|RTR|Complex|... *

>Cluster 607

0 876aa, >Anae109_2904||HK|Hybrid|TR|Pair|... *

>Cluster 608

0 876aa, >Hoch_2554||HK|Unorthodox|TRRH|Complex|... *

>Cluster 609

0 875aa, >Hoch_6888||RR|unclassified|R|Orphan|... *

>Cluster 610

0 865aa, >Hoch_6547||HK|Hybrid|TR|Complex|... *

>Cluster 611

0 864aa, >Anae109_3017||HK|Hybrid|TR|Pair|... *

>Cluster 612

0 859aa, >Hoch_3157||HK|Hybrid|TR|Pair|... *

>Cluster 613

0 859aa, >sce0817||HK|Hybrid|TRT|Complex|... *

>Cluster 614

0 855aa, >COCOR_06126||HK|Hybrid|TRT|Complex|... *

>Cluster 615

0 855aa, >STAUR_1880||HK|Hybrid|RT|Pair|... *

>Cluster 616

0 850aa, >LILAB_12350||PP|HisKa|H|Orphan|... *

>Cluster 617

0 850aa, >STAUR_7795||HK|Classic|T|Orphan|... *

>Cluster 618

0 850aa, >sce6042||HK|Classic|T|Orphan|... *

>Cluster 619

0 849aa, >COCOR_04617||HK|Hybrid|RTR|Complex|... *

>Cluster 620

0 846aa, >sce0186||HK|Classic|T|Orphan|... *

>Cluster 621

0 846aa, >sce8086||HK|Classic|T|Orphan|... *

>Cluster 622

0 837aa, >Hoch_5118||HK|Hybrid|TRT|Complex|... *

>Cluster 623

0 835aa, >Hoch_4029||HK|Hybrid|TRR|Complex|... *

>Cluster 624

0 834aa, >STAUR_7568||HK|Hybrid|RTR|Complex|... *

>Cluster 625

0 833aa, >STAUR_2444||HK|Classic|T|Orphan|... *

>Cluster 626

0 831aa, >STAUR_2227||HK|Classic|T|Pair|... *

>Cluster 627

0 830aa, >Anae109_0469||HK|CheA|TR|Complex|... *

>Cluster 628

0 830aa, >Hoch_5275||HK|Hybrid|TR|Pair|... *

>Cluster 629

0 823aa, >Anae109_2543||HK|Hybrid|TR|Pair|... *

>Cluster 630

0 816aa, >Anae109_0539||HK|Hybrid|TRT|Complex|... *

>Cluster 631

0 814aa, >sce3207||HK|Classic|T|Pair|... *

>Cluster 632

0 812aa, >STAUR_4443||HK|Classic|T|Orphan|... *

>Cluster 633^

0 808aa, >Anae109_3533||HK|CheA|TR|Complex|... *

>Cluster 634

0 805aa, >COCOR_00357||HK|Hybrid|TR|Pair|... *

>Cluster 635

0 805aa, >COCOR_01662||HK|Classic|T|Orphan|... *

>Cluster 636

0 804aa, >COCOR_04614||HK|Classic|T|Complex|... *

>Cluster 637

0 800aa, >COCOR_06733||HK|Classic|T|Orphan|... *

>Cluster 638

0 799aa, >Anae109_2297||HK|CheA|TR|Complex|... *

>Cluster 639

0 798aa, >Hoch_3782||HK|Classic|T|Orphan|... *

>Cluster 640

0 797aa, >COCOR_03260||HK|Classic|T|Orphan|... *

>Cluster 641

0 795aa, >PPSIR1_08966||HK|CheA|T||... *

>Cluster 642

0 794aa, >sce2761||HK|Classic|T|Orphan|... *

>Cluster 643

0 791aa, >Hoch_2563||PP|HisKa|H|Complex|... *

>Cluster 644

0 790aa, >Hoch_6787||HK|CheA|TR|Complex|... *

>Cluster 645

0 787aa, >sce2791||HK|Classic|T|Pair|... *

>Cluster 646

0 786aa, >Hoch_5915||HK|Hybrid|TR|Pair|... *

>Cluster 647

0 785aa, >sce0195||HK|Classic|T|Orphan|... *

>Cluster 648

0 783aa, >PPSIR1_08237||HK|Classic|T||... *

>Cluster 649

0 779aa, >sce0929||HK|Hybrid|TR|Pair|... *

>Cluster 650

0 776aa, >PPSIR1_09640||HK|Hybrid|TR||... *

>Cluster 651

0 774aa, >Hoch_4724||HK|Classic|T|Complex|... *

>Cluster 652

0 773aa, >Hoch_4048||HK|Classic|T|Orphan|... *

>Cluster 653

0 771aa, >PPSIR1_03623||HK|Hybrid|TR||... *

>Cluster 654

0 765aa, >Hoch_3203||HK|Hybrid|TR|Pair|... *

>Cluster 655

0 763aa, >STAUR_5925||HK|Classic|T|Orphan|... *

>Cluster 656

0 761aa, >LILAB_09595||HK|Hybrid|TRR|Complex|... *

>Cluster 657

0 760aa, >STAUR_0500||HK|Classic|T|Orphan|... *

>Cluster 658

0 760aa, >STAUR_8015||HK|Classic|T|Complex|... *

>Cluster 659

0 760aa, >sce0412||RR|RpfG|R|Orphan|... *

>Cluster 660

0 757aa, >STAUR_5807||HK|Classic|T|Pair|... *

>Cluster 661

0 756aa, >Hoch_3253||HK|CheA|TR|Complex|... *

>Cluster 662

0 754aa, >sce1594||HK|CheA|T|Pair|... *

>Cluster 663

0 752aa, >STAUR_0751||HK|Classic|T|Orphan|... *

>Cluster 664

0 752aa, >Hoch_5559||HK|Classic|T|Complex|... *

>Cluster 665

0 743aa, >PPSIR1_02491||HK|Hybrid|TR||... *

>Cluster 666

0 742aa, >PPSIR1_29603||HK|Classic|T||... *

>Cluster 667

0 742aa, >sce8135||HK|Hybrid|TR|Pair|... *

>Cluster 668

0 741aa, >sce1396||HK|Classic|T|Pair|... *

>Cluster 669

0 740aa, >sce0428||HK|Classic|T|Pair|... *

>Cluster 670

0 739aa, >PPSIR1_31378||HK|Hybrid|TR||... *

>Cluster 671

0 739aa, >sce4548||HK|Hybrid|TR|Pair|... *

>Cluster 672

0 738aa, >sce2461||HK|Classic|T|Orphan|... *

>Cluster 673

0 737aa, >PPSIR1_03968||RR|CheY|R||... *

>Cluster 674

0 734aa, >Anae109_2365||HK|Hybrid|RRRT|Complex|... *

>Cluster 675

0 732aa, >sce3940||HK|Hybrid|TR|Pair|... *

>Cluster 676

0 730aa, >sce0777||HK|CheA|TR|Pair|... *

>Cluster 677

0 729aa, >STAUR_0106||HK|Hybrid|TR|Pair|... *

>Cluster 678

0 729aa, >PPSIR1_23399||HK|Classic|T||... *

>Cluster 679

0 729aa, >sce7806||HK|Hybrid|RT|Complex|... *

>Cluster 680

0 725aa, >Anae109_3795||HK|Hybrid|TR|Orphan|... *

>Cluster 681

0 724aa, >STAUR_4406||HK|Classic|T|Orphan|... *

>Cluster 682

0 722aa, >COCOR_04882||HK|CheA|T|Orphan|... *

>Cluster 683

0 719aa, >PPSIR1_21484||HK|Hybrid|TR||... *

>Cluster 684

0 718aa, >Anae109_0803||HK|Hybrid|TR|Complex|... *

>Cluster 685

0 717aa, >Hoch_5872||HK|Classic|T|Pair|... *

>Cluster 686

0 716aa, >MXAN_3606||HK|Classic|T|Pair|... *

>Cluster 687

0 716aa, >Anae109_2235||HK|Hybrid|RT|Complex|... *

>Cluster 688

0 715aa, >sce8125||HK|Hybrid|RT|Pair|... *

>Cluster 689

0 714aa, >STAUR_7897||HK|Hybrid|TR|Pair|... *

>Cluster 690

0 714aa, >sce6826||HK|Hybrid|RTR|Complex|... *

>Cluster 691

0 713aa, >sce1260||HK|Hybrid|RTR|Complex|... *

>Cluster 692

0 711aa, >sce0589||HK|Hybrid|TR|Orphan|... *

>Cluster 693

0 707aa, >Hoch_2230||HK|Hybrid|TR|Pair|... *

>Cluster 694

0 707aa, >sce2236||HK|Classic|T|Orphan|... *

>Cluster 695

0 706aa, >COCOR_07355||HK|Hybrid|TR|Pair|... *

>Cluster 696

0 706aa, >STAUR_8326||HK|Classic|T|Orphan|... *

>Cluster 697

0 704aa, >PPSIR1_18507||HK|Classic|T||... *

>Cluster 698

0 703aa, >Anae109_1386A||HK|Classic|T|Pair|... *

>Cluster 699

0 700aa, >sce2679||HK|Hybrid|TR|Complex|... *

>Cluster 700

0 700aa, >sce5484||HK|Classic|T|Pair|... *

>Cluster 701

0 699aa, >Hoch_5280||HK|Classic|T|Orphan|... *

>Cluster 702

0 697aa, >STAUR_0409||RR|unclassified|HRR|Complex|... *

>Cluster 703

0 697aa, >STAUR_0489||HK|CheA|TR|Complex|... *

>Cluster 704

0 695aa, >sce7931||HK|Classic|T|Complex|... *

>Cluster 705

0 692aa, >sce5356||HK|Hybrid|RT|Pair|... *

>Cluster 706

0 691aa, >PPSIR1_10775||HK|Hybrid|TR||... *

>Cluster 707

0 688aa, >Anae109_2367||HK|CheA|TR|Complex|... *

>Cluster 708

0 688aa, >Hoch_6389||HK|Hybrid|TR|Pair|... *

>Cluster 709

0 687aa, >Hoch_4621||RR|CyC-C|R|Pair|... *

>Cluster 710

0 687aa, >Hoch_4861||HK|Classic|T|Orphan|... *

>Cluster 711

0 686aa, >PPSIR1_30554||HK|Hybrid|TR||... *

>Cluster 712

0 683aa, >STAUR_7155||HK|Hybrid|TR|Pair|... *

>Cluster 713

0 683aa, >A176_2141||HK|Classic|T||... *

>Cluster 714

0 682aa, >STAUR_1669||HK|Classic|T|Orphan|... *

>Cluster 715

0 681aa, >PPSIR1_23404||HK|Hybrid|RTR||... *

>Cluster 716

0 679aa, >AnaeK_3949||HK|Classic|T|Orphan|... *

>Cluster 717

0 679aa, >PPSIR1_22951||HK|Classic|T||... *

>Cluster 718

0 678aa, >Hoch_4576||HK|Hybrid|TR|Pair|... *

>Cluster 719

0 677aa, >Hoch_5080||HK|Classic|T|Orphan|... *

>Cluster 720

0 674aa, >Anae109_4251||HK|Classic|T|Complex|... *

>Cluster 721

0 672aa, >PPSIR1_40839||HK|Hybrid|TR||... *

>Cluster 722

0 672aa, >Hoch_1709||RR|unclassified|R|Orphan|... *

>Cluster 723

0 671aa, >sce1189||HK|Classic|T|Pair|... *

>Cluster 724

0 669aa, >sce4957||HK|Hybrid|TR|Complex|... *

>Cluster 725

0 667aa, >LILAB_26115||HK|Classic|T|Orphan|... *

>Cluster 726

0 667aa, >COCOR_07394||HK|Hybrid|TR|Pair|... *

>Cluster 727

0 667aa, >sce7217||HK|Classic|T|Complex|... *

>Cluster 728

0 665aa, >STAUR_2755||HK|Classic|T|Orphan|... *

>Cluster 729

0 663aa, >Anae109_0804||HK|Hybrid|RT|Complex|... *

>Cluster 730

0 660aa, >STAUR_8136||HK|Classic|T|Orphan|... *

>Cluster 731

0 660aa, >Hoch_2685||HK|Hybrid|TR|Pair|... *

>Cluster 732

0 659aa, >Anae109_0529||HK|Hybrid|TR|Pair|... *

>Cluster 733

0 657aa, >PPSIR1_12318||HK|Hybrid|TR||... *

>Cluster 734

0 654aa, >STAUR_2158||HK|Classic|T|Orphan|... *

>Cluster 735

0 654aa, >PPSIR1_42266||HK|Hybrid|TR||... *

>Cluster 736

0 653aa, >PPSIR1_22956||HK|Classic|T||... *

>Cluster 737

0 652aa, >sce0962||HK|Classic|T|Orphan|... *

>Cluster 738

0 651aa, >COCOR_05286||HK|Hybrid|TR|Pair|... *

>Cluster 739

0 651aa, >sce8990||HK|Classic|T|Orphan|... *

>Cluster 740

0 650aa, >Hoch_1933||HK|Hybrid|TR|Pair|... *

>Cluster 741

0 648aa, >STAUR_0448||HK|Hybrid|RTR|Complex|... *

>Cluster 742

0 647aa, >STAUR_4227||HK|Hybrid|TR|Pair|... *

>Cluster 743

0 647aa, >STAUR_5194||HK|Classic|T|Orphan|... *

>Cluster 744

0 647aa, >Hoch_0223||HK|Hybrid|RTR|Complex|... *

>Cluster 745

0 646aa, >A176_0342||HK|Classic|T||... *

>Cluster 746

0 644aa, >Hoch_4645||HK|Hybrid|RTR|Complex|... *

>Cluster 747

0 643aa, >Hoch_5362||HK|Hybrid|RT|Pair|... *

>Cluster 748

0 641aa, >COCOR_00395||HK|Classic|T|Complex|... *

>Cluster 749

0 639aa, >PPSIR1_01047||HK|Hybrid|TR||... *

>Cluster 750

0 639aa, >sce7215||HK|Classic|T|Complex|... *

>Cluster 751

0 639aa, >sce8828||HK|Hybrid|TR|Pair|... *

>Cluster 752

0 637aa, >sce2678||HK|Classic|T|Complex|... *

>Cluster 753

0 636aa, >COCOR_01853||HK|Classic|T|Pair|... *

>Cluster 754

0 636aa, >sce0723||HK|Hybrid|RTR|Complex|... *

>Cluster 755

0 636aa, >sce3080||HK|Classic|T|Orphan|... *

>Cluster 756

0 635aa, >PPSIR1_04853||HK|Hybrid|TR||... *

>Cluster 757

0 634aa, >STAUR_1486||HK|Hybrid|RT|Pair|... *

>Cluster 758

0 633aa, >Hoch_2174||RR|Unclassified|HR|Complex|... *

>Cluster 759

0 631aa, >A176_5734||HK|Hybrid|RT||... *

>Cluster 760

0 628aa, >Hoch_1359||HK|Classic|T|Pair|... *

>Cluster 761

0 628aa, >sce8154||RR|PleD|R|Complex|... *

>Cluster 762

0 627aa, >STAUR_6568||HK|Classic|T|Pair|... *

>Cluster 763

0 626aa, >MXAN_7366||PP|HisKa|H|Complex|... *

>Cluster 764

0 626aa, >PPSIR1_19849||HK|Hybrid|TR||... *

>Cluster 765

0 623aa, >STAUR_0301||HK|Classic|T|Pair|... *

>Cluster 766

0 623aa, >Anae109_2929||HK|Hybrid|TR|Pair|... *

>Cluster 767

0 623aa, >sce0350||HK|Classic|T|Orphan|... *

>Cluster 768

0 619aa, >sce4309||HK|Hybrid|TRH|Complex|... *

>Cluster 769

0 617aa, >STAUR_1167||HK|Classic|T|Complex|... *

>Cluster 770

0 615aa, >Anae109_0966||HK|Classic|T|Pair|... *

>Cluster 771

0 615aa, >PPSIR1_08147||HK|Classic|T||... *

>Cluster 772

0 614aa, >Hoch_3516||HK|Hybrid|TR|Pair|... *

>Cluster 773

0 613aa, >sce9207||RR|unclassified|R|Orphan|... *

>Cluster 774

0 612aa, >STAUR_0728||HK|Classic|T|Pair|... *

>Cluster 775

0 610aa, >Hoch_4438||HK|Classic|T|Pair|... *

>Cluster 776

0 610aa, >sce1383||HK|Classic|T|Orphan|... *

>Cluster 777

0 604aa, >sce2974||RR|NtrC|R|Orphan|... *

>Cluster 778

0 602aa, >PPSIR1_28303||HK|Classic|T||... *

>Cluster 779

0 602aa, >PPSIR1_31903||HK|Classic|T||... *

>Cluster 780

0 602aa, >Hoch_1970||HK|Hybrid|TR|Pair|... *

>Cluster 781

0 602aa, >Hoch_5658||HK|Classic|T|Orphan|... *

>Cluster 782

0 602aa, >Hoch_5853||HK|Classic|T|Pair|... *

>Cluster 783

0 601aa, >sce1690||HK|Classic|T|Complex|... *

>Cluster 784

0 600aa, >Anae109_1855||HK|Classic|T|Complex|... *

>Cluster 785

0 597aa, >Hoch_4278||HK|Classic|T|Pair|... *

>Cluster 786

0 597aa, >sce3423||HK|Classic|T|Orphan|... *

>Cluster 787

0 596aa, >PPSIR1_35832||HK|Hybrid|TR||... *

>Cluster 788

0 595aa, >sce9291||HK|Classic|T|Orphan||... *

>Cluster 789

0 593aa, >Hoch_5165||HK|Hybrid|RTR|Complex|... *

>Cluster 790

0 590aa, >PPSIR1_07480||HK|Classic|T||... *

>Cluster 791

0 586aa, >Adeh_1706A||HK|Classic|T|Orphan|... *

>Cluster 792

0 586aa, >Hoch_5107||RR|unclassified|R|Orphan|... *

>Cluster 793

0 586aa, >sce5774||HK|Classic|T|Orphan|... *

>Cluster 794

0 586aa, >sce7406||HK|Classic|T|Orphan|... *

>Cluster 795

0 584aa, >PPSIR1_40914||HK|Hybrid|TR||... *

>Cluster 796

0 584aa, >sce3998||RR|CyC-C|R|Orphan|... *

>Cluster 797

0 583aa, >PPSIR1_17495||HK|Classic|T||... *

>Cluster 798

0 582aa, >Hoch_4825||HK|Classic|T|Orphan|... *

>Cluster 799

0 574aa, >PPSIR1_04383||RR|unclassified|R||... *

>Cluster 800

0 573aa, >PPSIR1_31808||HK|Hybrid|TR||... *

>Cluster 801

0 572aa, >Hoch_2075||HK|Hybrid|RTR|Complex|... *

>Cluster 802

0 571aa, >STAUR_7221||RR|unclassified|R|Complex|... *

>Cluster 803

0 571aa, >STAUR_7890||HK|Hybrid|RT|Pair|... *

>Cluster 804

0 571aa, >Hoch_6003||HK|Classic|T|Pair|... *

>Cluster 805

0 569aa, >PPSIR1_37179||HK|Hybrid|TR||... *

>Cluster 806

0 566aa, >STAUR_2931||HK|Classic|T|Orphan|... *

>Cluster 807

0 566aa, >PPSIR1_40005||HK|Classic|T||... *

>Cluster 808

0 564aa, >COCOR_04772||HK|Classic|T|Orphan|... *

>Cluster 809

0 563aa, >sce0516||HK|Classic|T|Orphan|... *

>Cluster 810

0 563aa, >sce1653||RR|VieB|R|Orphan|... *

>Cluster 811

0 561aa, >sce1938||RR|unclassified|R|Complex|... *

>Cluster 812

0 559aa, >Hoch_2564||HK|Classic|T|Complex|... *

>Cluster 813

0 558aa, >PPSIR1_31658||HK|Classic|T||... *

>Cluster 814

0 558aa, >sce7929||HK|Classic|T|Complex|... *

>Cluster 815

0 557aa, >Anae109_1033||HK|Classic|T|Orphan|... *

>Cluster 816

0 556aa, >STAUR_8016||HK|Hybrid|RT|Complex|... *

>Cluster 817

0 555aa, >Anae109_3354||HK|Classic|T|Pair|... *

>Cluster 818

0 554aa, >COCOR_06970||HK|Classic|T|Orphan|... *

>Cluster 819

0 553aa, >sce8434||HK|Hybrid|TR|Complex|... *

>Cluster 820

0 552aa, >COCOR_01048||HK|Classic|T|Pair|... *

>Cluster 821

0 551aa, >Anae109_4054||HK|Hybrid|TR|Pair|... *

>Cluster 822

0 550aa, >sce8153||RR|unclassified|R|Complex|... *

>Cluster 823

0 549aa, >STAUR_2826||HK|Classic|T|Orphan|... *

>Cluster 824

0 549aa, >Anae109_3005||HK|Classic|T|Orphan|... *

>Cluster 825

0 549aa, >sce1173||HK|Hybrid|RT|Complex|... *

>Cluster 826

0 549aa, >sce1742||HK|Hybrid|TR|Pair|... *

>Cluster 827

0 548aa, >Hoch_1875||HK|Classic|T|Pair|... *

>Cluster 828

0 547aa, >sce8644||HK|Classic|T|Orphan|... *

>Cluster 829

0 546aa, >MXAN_5340||RR|PleD|R|Orphan|... *

>Cluster 830

0 545aa, >sce3598||HK|Hybrid|TR|Complex|... *

>Cluster 831

0 545aa, >sce8713||HK|Hybrid|RT|Complex|... *

>Cluster 832

0 544aa, >COCOR_07556||HK|Hybrid|RT|Complex|... *

>Cluster 833

0 542aa, >STAUR_6693||HK|Classic|T|Pair|... *

>Cluster 834

0 540aa, >Hoch_3122||HK|Hybrid|TR|Pair|... *

>Cluster 835

0 540aa, >Hoch_5571||HK|Classic|T|Orphan|... *

>Cluster 836

0 540aa, >Hoch_5638||HK|Classic|T|Orphan|... *

>Cluster 837

0 539aa, >sce6012||HK|Classic|T|Complex|... *

>Cluster 838

0 538aa, >Hoch_0267||HK|Classic|T|Pair|... *

>Cluster 839

0 537aa, >Anae109_1597||HK|Hybrid|TR|Pair|... *

>Cluster 840

0 536aa, >sce2792||RR|NtrC|R|Pair|... *

>Cluster 841

0 534aa, >sce0385||HK|Classic|T|Orphan|... *

>Cluster 842

0 532aa, >Hoch_6114||RR|unclassified|R|Orphan|... *

>Cluster 843

0 532aa, >Hoch_6820||HK|Classic|T|Orphan|... *

>Cluster 844

0 531aa, >sce7876||HK|Classic|T|Orphan|... *

>Cluster 845

0 530aa, >COCOR_07586||HK|Classic|T|Orphan|... *

>Cluster 846

0 530aa, >STAUR_4763||HK|Classic|T|Orphan|... *

>Cluster 847

0 528aa, >MXAN_3601||HK|Classic|T|Orphan|... *

>Cluster 848

0 528aa, >COCOR_07701||HK|Classic|T|Pair|... *

>Cluster 849

0 526aa, >COCOR_07951||HK|Classic|T|Complex|... *

>Cluster 850

0 526aa, >Anae109_4186||HK|Classic|T|Orphan|... *

>Cluster 851

0 526aa, >Hoch_4444||RR|RpfG|R|Orphan|... *

>Cluster 852

0 526aa, >sce8798||HK|Classic|T|Complex|... *

>Cluster 853

0 524aa, >Hoch_5521||HK|Hybrid|RT|Complex|... *

>Cluster 854

0 522aa, >Anae109_2005||HK|Hybrid|TR|Pair|... *

>Cluster 855

0 521aa, >STAUR_8013||HK|Hybrid|RT|Complex|... *

>Cluster 856

0 520aa, >sce7214||HK|Classic|T|Complex|... *

>Cluster 857

0 519aa, >Anae109_1492||HK|Hybrid|TR|Pair|... *

>Cluster 858

0 519aa, >Hoch_6238||HK|Hybrid|RTR|Complex|... *

>Cluster 859

0 518aa, >Anae109_2560||HK|Classic|T|Orphan|... *

>Cluster 860

0 517aa, >sce3063||HK|Hybrid|TR|Pair|... *

>Cluster 861

0 516aa, >Hoch_5988||HK|Classic|T|Pair|... *

>Cluster 862

0 516aa, >sce2506||HK|Classic|T|Pair|... *

>Cluster 863

0 516aa, >sce2546||RR|RpfG|R|Orphan|... *

>Cluster 864

0 516aa, >sce4877||HK|Classic|T|Pair|... *

>Cluster 865

0 515aa, >PPSIR1_16500||HK|Classic|T||... *

>Cluster 866

0 515aa, >Hoch_0050||RR|NtrC|R|Orphan|... *

>Cluster 867

0 514aa, >COCOR_07540||HK|Hybrid|RT|Complex|... *

>Cluster 868

0 514aa, >STAUR_8087||HK|Classic|T|Pair|... *

>Cluster 869

0 514aa, >Anae109_4365||HK|Classic|T|Complex|... *

>Cluster 870

0 514aa, >Hoch_4666||HK|Classic|T|Complex|... *

>Cluster 871

0 512aa, >sce7761||HK|Classic|T|Pair|... *

>Cluster 872

0 511aa, >PPSIR1_04723||HK|Classic|T||... *

>Cluster 873

0 511aa, >sce5167||HK|Classic|T|Complex|... *

>Cluster 874

0 511aa, >sce8402||HK|Classic|T|Orphan|... *

>Cluster 875

0 510aa, >PPSIR1_21804||HK|Classic|T||... *

>Cluster 876

0 510aa, >Hoch_5744||HK|Hybrid|TR|Pair|... *

>Cluster 877

0 509aa, >PPSIR1_22586||HK|Classic|T||... *

>Cluster 878

0 509aa, >Hoch_1847||HK|Classic|T|Pair|... *

>Cluster 879

0 509aa, >sce7800||HK|Classic|T|Pair|... *

>Cluster 880

0 508aa, >STAUR_0581||HK|Classic|T|Orphan|... *

>Cluster 881

0 507aa, >Hoch_4844||HK|Hybrid|TR|Pair|... *

>Cluster 882

0 506aa, >sce7107||HK|Classic|T|Orphan|... *

>Cluster 883

0 505aa, >PPSIR1_20094||HK|Classic|T||... *

>Cluster 884

0 504aa, >sce1800||HK|Hybrid|RT|Pair|... *

>Cluster 885

0 503aa, >PPSIR1_13860||HK|Classic|T||... *

>Cluster 886

0 503aa, >PPSIR1_36954||HK|Classic|T||... *

>Cluster 887

0 503aa, >sce6354||HK|Classic|T|Pair|... *

>Cluster 888

0 502aa, >STAUR_7219||HK|Classic|T|Complex|... *

>Cluster 889

0 502aa, >Anae109_2142||HK|Classic|T|Orphan|... *

>Cluster 890

0 501aa, >STAUR_0010||HK|Classic|T|Pair|... *

>Cluster 891

0 501aa, >Anae109_2823||HK|Classic|T|Orphan|... *

>Cluster 892

0 501aa, >Hoch_4280||RR|NtrC|R|Pair|... *

>Cluster 893

0 500aa, >sce2313||RR|unclassified|R|Complex|... *

>Cluster 894

0 499aa, >STAUR_5537||HK|Classic|T|Pair|... *

>Cluster 895

0 499aa, >Anae109_1487||RR|unclassified|R|Orphan|... *

>Cluster 896

0 499aa, >Hoch_3001||HK|Classic|T|Pair|... *

>Cluster 897

0 498aa, >COCOR_02143||HK|Classic|T|Pair|... *

>Cluster 898

0 498aa, >Hoch_5029||HK|Hybrid|TR|Pair|... *

>Cluster 899

0 498aa, >sce2947||HK|Classic|T|Pair|... *

>Cluster 900

0 498aa, >sce8108||HK|Classic|T|Pair|... *

>Cluster 901

0 496aa, >COCOR_00378||HK|Classic|T|Orphan|... *

>Cluster 902

0 496aa, >Hoch_3303||HK|Classic|T|Pair|... *

>Cluster 903

0 495aa, >PPSIR1_07538||HK|Classic|T||... *

>Cluster 904

0 495aa, >PPSIR1_22094||HK|Classic|T||... *

>Cluster 905

0 495aa, >sce3208||RR|NtrC|R|Pair|... *

>Cluster 906

0 494aa, >sce6174||RR|PrrA|R|Orphan|... *

>Cluster 907

0 494aa, >sce6249||HK|Classic|T|Orphan|... *

>Cluster 908

0 493aa, >Hoch_0247||RR|NtrC|R|Orphan|... *

>Cluster 909

0 492aa, >PPSIR1_22776||PP|HPt|H||... *

>Cluster 910

0 491aa, >MXAN_3419||HK|Classic|T|Pair|... *

>Cluster 911

0 491aa, >STAUR_7541||HK|Classic|T|Orphan|... *

>Cluster 912

0 491aa, >sce6964||HK|Classic|T|Complex|... *

>Cluster 913

0 490aa, >MXAN_5211||HK|Classic|T|Pair|... *

>Cluster 914

0 490aa, >sce2050||HK|Classic|T|Complex|... *

>Cluster 915

0 490aa, >sce7596||HK|Classic|T|Pair|... *

>Cluster 916

0 488aa, >STAUR_8018||HK|Classic|T|Complex|... *

>Cluster 917

0 488aa, >AnaeK_3250||HK|Classic|T|Pair|... *

>Cluster 918

0 488aa, >Anae109_0446||HK|Classic|T|Orphan|... *

>Cluster 919

0 488aa, >Hoch_0286||RR|NtrC|R|Complex|... *

>Cluster 920

0 487aa, >STAUR_0798||HK|Classic|T|Orphan|... *

>Cluster 921

0 487aa, >sce2848||RR|NtrC|R|Complex|... *

>Cluster 922

0 486aa, >PPSIR1_23114||RR|NtrC|R||... *

>Cluster 923

0 486aa, >PPSIR1_30746||HK|Hybrid|TR||... *

>Cluster 924

0 485aa, >Anae109_3068||HK|Classic|T|Pair|... *

>Cluster 925

0 484aa, >Hoch_5873||RR|NtrC|R|Pair|... *

>Cluster 926

0 484aa, >sce0947||HK|Classic|T|Orphan|... *

>Cluster 927

0 480aa, >Hoch_2279||HK|Classic|T|Complex|... *

>Cluster 928

0 478aa, >STAUR_7553||HK|Classic|T|Orphan|... *

>Cluster 929

0 477aa, >PPSIR1_26578||RR|NtrC|R||... *

>Cluster 930

0 475aa, >STAUR_4794||RR|CheY|R|Orphan|... *

>Cluster 931

0 475aa, >STAUR_7113||HK|Classic|T|Complex|... *

>Cluster 932

0 475aa, >sce8043||HK|Classic|T|Orphan|... *

>Cluster 933

0 472aa, >Hoch_3302||RR|NtrC|R|Pair|... *

>Cluster 934

0 471aa, >sce1418||HK|Classic|T|Pair|... *

>Cluster 935

0 471aa, >sce8107||RR|NtrC|R|Pair|... *

>Cluster 936

0 470aa, >Hoch_1979||RR|PleD|R|Orphan|... *

>Cluster 937

0 469aa, >A2cp1_3137||HK|Classic|T|Complex|... *

>Cluster 938

0 469aa, >sce1137||HK|Classic|T|Orphan|... *

>Cluster 939

0 469aa, >sce8837||RR|NtrC|R|Pair|... *

>Cluster 940

0 468aa, >PPSIR1_09415||HK|Classic|T||... *

>Cluster 941

0 467aa, >COCOR_03494||HK|Classic|T|Orphan|... *

>Cluster 942

0 466aa, >Hoch_5854||RR|NtrC|R|Pair|... *

>Cluster 943

0 465aa, >AnaeK_3251||RR|NtrC|R|Pair|... *

>Cluster 944

0 465aa, >Hoch_6828||HK|Classic|T|Pair|... *

>Cluster 945

0 463aa, >STAUR_1216||HK|Classic|T|Orphan|... *

>Cluster 946

0 463aa, >STAUR_5783||RR|CheY|R|Orphan|... *

>Cluster 947

0 463aa, >PPSIR1_07073||HK|Classic|T||... *

>Cluster 948

0 462aa, >Anae109_1870||HK|Classic|T|Orphan|... *

>Cluster 949

0 462aa, >Hoch_5338||RR|NtrC|R|Orphan|... *

>Cluster 950

0 461aa, >STAUR_1276||HK|Classic|T|Orphan|... *

>Cluster 951

0 461aa, >STAUR_4617||HK|Classic|T|Pair|... *

>Cluster 952

0 461aa, >Anae109_0106||HK|Classic|T|Orphan|... *

>Cluster 953

0 461aa, >sce8268||RR|unclassified|R|Orphan|... *

>Cluster 954

0 460aa, >Anae109_0030||HK|Classic|T|Orphan|... *

>Cluster 955

0 460aa, >Hoch_5051||RR|NtrC|R|Orphan|... *

>Cluster 956

0 458aa, >sce2554||HK|Classic|T|Pair|... *

>Cluster 957

0 457aa, >STAUR_0773||HK|Classic|T|Pair|... *

>Cluster 958

0 457aa, >sce3264||RR|NtrC|R|Pair|... *

>Cluster 959

0 456aa, >PPSIR1_42119||HK|Classic|T||... *

>Cluster 960

0 456aa, >sce3287||HK|Classic|T|Pair|... *

>Cluster 961

0 452aa, >PPSIR1_33209||HK|Classic|T||... *

>Cluster 962

0 452aa, >sce8836||HK|Classic|T|Pair|... *

>Cluster 963

0 451aa, >Hoch_5844||HK|Classic|T|Pair|... *

>Cluster 964

0 451aa, >sce4025||HK|Classic|T|Complex|... *

>Cluster 965

0 451aa, >sce4827||HK|Classic|T|Pair|... *

>Cluster 966

0 450aa, >STAUR_3095||HK|Classic|T|Pair|... *

>Cluster 967

0 449aa, >COCOR_05932||HK|Classic|T|Pair|... *

>Cluster 968

0 449aa, >STAUR_5209||HK|Classic|T|Orphan|... *

>Cluster 969

0 449aa, >Hoch_6695||RR|Unclassified|RH|Complex|... *

>Cluster 970

0 448aa, >PPSIR1_33109||HK|Classic|T||... *

>Cluster 971

0 448aa, >Hoch_5447||HK|Classic|T|Pair|... *

>Cluster 972

0 447aa, >PPSIR1_17300||RR|unclassified|R||... *

>Cluster 973

0 446aa, >LILAB_08870||RR|unclassified|R|Orphan|... *

>Cluster 974

0 444aa, >Hoch_3340||HK|Classic|T|Pair|... *

>Cluster 975

0 443aa, >STAUR_1481||HK|Classic|T|Complex|... *

>Cluster 976

0 442aa, >PPSIR1_08891||PP|HisKa|H||... *

>Cluster 977

0 440aa, >STAUR_7786||HK|Classic|T|Complex|... *

>Cluster 978

0 440aa, >Hoch_3223||HK|Classic|T|Pair|... *

>Cluster 979

0 438aa, >sce3704||HK|Hybrid|RT|Pair|... *

>Cluster 980

0 437aa, >COCOR_00919||HK|Classic|T|Pair|... *

>Cluster 981

0 437aa, >Anae109_3161||HK|Classic|T|Orphan|... *

>Cluster 982

0 435aa, >COCOR_01758||HK|Classic|T|Orphan|... *

>Cluster 983

0 433aa, >sce8052||HK|Classic|T|Pair|... *

>Cluster 984

0 432aa, >PPSIR1_02818||RR|CheY|R||... *

>Cluster 985

0 431aa, >STAUR_0866||HK|Classic|T|Pair|... *

>Cluster 986

0 431aa, >STAUR_5904||HK|Classic|T|Pair|... *

>Cluster 987

0 431aa, >Anae109_1863||RR|unclassified|R|Orphan|... *

>Cluster 988

0 430aa, >A176_0859||HK|Classic|T||... *

>Cluster 989

0 430aa, >sce0932||HK|Classic|T|Orphan|... *

>Cluster 990

0 430aa, >sce7298||RR|unclassified|R|Orphan|... *

>Cluster 991

0 428aa, >STAUR_1902||HK|Classic|T|Orphan|... *

>Cluster 992

0 425aa, >STAUR_6644||HK|Classic|T|Orphan|... *

>Cluster 993

0 423aa, >Hoch_5522||HK|Classic|T|Complex|... *

>Cluster 994

0 422aa, >PPSIR1_16305||HK|Classic|T||... *

>Cluster 995

0 422aa, >PPSIR1_40560||HK|Classic|T||... *

>Cluster 996

0 421aa, >Hoch_4643||HK|Classic|T|Pair|... *

>Cluster 997

0 420aa, >Hoch_5544||HK|Classic|T|Pair|... *

>Cluster 998

0 419aa, >STAUR_0037||HK|Hybrid|RT|Pair|... *

>Cluster 999

0 419aa, >Hoch_5897||RR|unclassified|R|Orphan|... *

>Cluster 1000

0 418aa, >Hoch_3251||HK|Hybrid|RT|Complex|... *

>Cluster 1001

0 417aa, >sce1474||HK|Classic|T|Orphan|... *

>Cluster 1002

0 414aa, >sce2312||HK|Hybrid|RT|Complex|... *

>Cluster 1003

0 412aa, >Hoch_3476||RR|unclassified|R|Orphan|... *

>Cluster 1004

0 411aa, >Hoch_0285||HK|Hybrid|RT|Complex|... *

>Cluster 1005

0 410aa, >Hoch_3494||RR|unclassified|R|Orphan|... *

>Cluster 1006

0 410aa, >sce8707||HK|Hybrid|TR|Pair|... *

>Cluster 1007

0 409aa, >STAUR_7903||HK|Classic|T|Orphan|... *

>Cluster 1008

0 408aa, >Hoch_5143||HK|Hybrid|RT|Complex|... *

>Cluster 1009

0 408aa, >Hoch_5359||HK|Classic|T|Orphan|... *

>Cluster 1010

0 407aa, >STAUR_7046||HK|Classic|T|Orphan|... *

>Cluster 1011

0 407aa, >Adeh_0563||RR|unclassified|R|Complex|... *

>Cluster 1012

0 406aa, >COCOR_07893||HK|Classic|T|Orphan|... *

>Cluster 1013

0 406aa, >Hoch_2262||HK|Hybrid|RT|Pair|... *

>Cluster 1014

0 403aa, >COCOR_04661||RR|CheY|R|Pair|... *

>Cluster 1015

0 402aa, >STAUR_7487||HK|Classic|T|Orphan|... *

>Cluster 1016

0 402aa, >PPSIR1_34113||RR|VieA|R||... *

>Cluster 1017

0 402aa, >Hoch_5778||HK|Classic|TR|Pair|... *

>Cluster 1018

0 400aa, >Anae109_2387||HK|Classic|T|Orphan|... *

>Cluster 1019

0 399aa, >sce7218||HK|Classic|T|Complex|... *

>Cluster 1020

0 397aa, >STAUR_6078||HK|Hybrid|RT|Complex|... *

>Cluster 1021

0 397aa, >PPSIR1_38781||HK|Classic|T||... *

>Cluster 1022

0 397aa, >Hoch_2558||HK|Classic|T|Orphan|... *

>Cluster 1023

0 397aa, >Hoch_6548||HK|Hybrid|RT|Complex|... *

>Cluster 1024

0 396aa, >sce4026||HK|Hybrid|RT|Complex|... *

>Cluster 1025

0 395aa, >Hoch_2175||HK|Hybrid|RT|Complex|... *

>Cluster 1026

0 394aa, >Anae109_1538||HK|Classic|T|Orphan|... *

>Cluster 1027

0 394aa, >PPSIR1_42109||HK|Hybrid|RT||... *

>Cluster 1028

0 392aa, >STAUR_0844||HK|Classic|T|Orphan|... *

>Cluster 1029

0 392aa, >Hoch_6726||HK|Classic|T|Pair|... *

>Cluster 1030

0 391aa, >sce2847||RR|PleD|R|Complex|... *

>Cluster 1031

0 390aa, >Anae109_2934||HK|Classic|T|Orphan|... *

>Cluster 1032

0 389aa, >Hoch_6827||RR|unclassified|R|Pair|... *

>Cluster 1033

0 389aa, >sce8433||RR|CyC-C|R|Complex|... *

>Cluster 1034

0 387aa, >Anae109_3228||HK|Classic|T|Orphan|... *

>Cluster 1035

0 385aa, >sce5432||HK|Classic|T|Pair|... *

>Cluster 1036

0 384aa, >A176_7252||HK|Classic|T||... *

>Cluster 1037

0 382aa, >STAUR_4293||HK|Classic|T|Orphan|... *

>Cluster 1038

0 382aa, >PPSIR1_24879||HK|Classic|T||... *

>Cluster 1039

0 382aa, >sce2261||HK|Classic|T|Orphan|... *

>Cluster 1040

0 381aa, >STAUR_1483||HK|Classic|T|Complex|... *

>Cluster 1041

0 381aa, >Hoch_1901||RR|CyC-C|R|Orphan|... *

>Cluster 1042

0 380aa, >sce5815||HK|Classic|T|Complex|... *

>Cluster 1043

0 379aa, >PPSIR1_27323||HK|Classic|T||... *

>Cluster 1044

0 377aa, >PPSIR1_34143||RR|CheY|R||... *

>Cluster 1045

0 376aa, >Hoch_4668||HK|Classic|T|Complex|... *

>Cluster 1046

0 376aa, >sce4956||HK|Hybrid|RT|Complex|... *

>Cluster 1047

0 374aa, >PPSIR1_20329||HK|Classic|T||... *

>Cluster 1048

0 374aa, >Hoch_4800||HK|Classic|T|Pair|... *

>Cluster 1049

0 374aa, >Hoch_6704||RR|unclassified|R|Orphan|... *

>Cluster 1050

0 373aa, >COCOR_03825||RR|CheY|R|Orphan|... *

>Cluster 1051

0 373aa, >STAUR_6628||HK|Classic|T|Orphan|... *

>Cluster 1052

0 373aa, >PPSIR1_19949||HK|Classic|T||... *

>Cluster 1053

0 373aa, >A176_4969||HK|Classic|T||... *

>Cluster 1054

0 372aa, >PPSIR1_30474||RR|unclassified|R||... *

>Cluster 1055

0 372aa, >A176_6988||HK|Classic|T||... *

>Cluster 1056

0 368aa, >Hoch_4726||HK|Hybrid|RT|Complex|... *

>Cluster 1057

0 367aa, >COCOR_01809||HK|Classic|T|Pair|... *

>Cluster 1058

0 367aa, >Anae109_2099||HK|Classic|T|Orphan|... *

>Cluster 1059

0 366aa, >STAUR_4092||HK|Classic|T|Orphan|... *

>Cluster 1060

0 366aa, >Hoch_2452||HK|Classic|T|Orphan|... *

>Cluster 1061

0 365aa, >STAUR_0491||RR|FrzZ|RR|Complex|... *

>Cluster 1062

0 361aa, >STAUR_8301||HK|Classic|T|Pair|... *

>Cluster 1063

0 361aa, >PPSIR1_08996||RR|CheB|R||... *

>Cluster 1064

0 361aa, >PPSIR1_16905||HK|Hybrid|TR||... *

>Cluster 1065

0 360aa, >Hoch_4051||RR|PleD|R|Orphan|... *

>Cluster 1066

0 360aa, >sce1599||RR|CheB|R|Orphan|... *

>Cluster 1067

0 359aa, >Adeh_3556A||PP|HisKA|H|Orphan|... *

>Cluster 1068

0 357aa, >PPSIR1_30145||HK|Classic|T||... *

>Cluster 1069

0 356aa, >Hoch_5755||HK|Classic|T|Orphan|... *

>Cluster 1070

0 355aa, >sce5570||RR|unclassified|R|Complex|... *

>Cluster 1071

0 353aa, >PPSIR1_39855||PP|HisKa|H||... *

>Cluster 1072

0 350aa, >COCOR_02284||HK|Classic|T|Pair|... *

>Cluster 1073

0 349aa, >Hoch_3432||RR|unclassified|R|Orphan|... *

>Cluster 1074

0 347aa, >Hoch_3252||RR|CheB|R|Complex|... *

>Cluster 1075

0 347aa, >A176_6830||HK|Classic|T||... *

>Cluster 1076

0 345aa, >STAUR_3806||RR|CheB|R|Pair|... *

>Cluster 1077

0 343aa, >Hoch_6788||RR|CheB|R|Complex|... *

>Cluster 1078

0 342aa, >PPSIR1_03303||HK|Classic|T||... *

>Cluster 1079

0 341aa, >STAUR_3424||PP|HisKa|H|Complex|... *

>Cluster 1080

0 341aa, >sce2435||HK|Classic|T|Pair|... *

>Cluster 1081

0 340aa, >sce5483||RR|unclassified|R|Pair|... *

>Cluster 1082

0 335aa, >sce3988||RR|unclassified|R|Complex|... *

>Cluster 1083

0 332aa, >sce1296||HK|Classic|T|Orphan|... *

>Cluster 1084

0 330aa, >Hoch_1522||HK|Classic|T|Orphan|... *

>Cluster 1085

0 330aa, >sce2953||HK|Classic|T|Complex|... *

>Cluster 1086

0 326aa, >COCOR_05353||PP|HisKa|H|Orphan|... *

>Cluster 1087

0 322aa, >STAUR_6045||RR|PleD|R|Orphan|... *

>Cluster 1088

0 322aa, >PPSIR1_34517||PP|HisKa|H||... *

>Cluster 1089

0 321aa, >Hoch_0553||HK|Classic|T|Pair|... *

>Cluster 1090

0 321aa, >Hoch_6343||HK|Classic|T|Orphan|... *

>Cluster 1091

0 320aa, >PPSIR1_22886||RR|PleD|R||... *

>Cluster 1092

0 317aa, >Hoch_4952||RR|unclassified|R|Orphan|... *

>Cluster 1093

0 317aa, >sce2822||HK|Classic|T|Orphan|... *

>Cluster 1094

0 315aa, >MXAN_2116A||HK|Classic|T|Orphan|... *

>Cluster 1095

0 314aa, >sce1689||RR|unclassified|R|Complex|... *

>Cluster 1096

0 311aa, >PPSIR1_40290||HK|Classic|T||... *

>Cluster 1097

0 309aa, >PPSIR1_08232||RR|MerR|RR||... *

>Cluster 1098

0 307aa, >COCOR_07702||RR|CheY|R|Pair|... *

>Cluster 1099

0 307aa, >sce7775||RR|unclassified|R|Complex|... *

>Cluster 1100

0 306aa, >PPSIR1_14510||RR|PleD|R||... *

>Cluster 1101

0 305aa, >Hoch_5056||RR|unclassified|R|Orphan|... *

>Cluster 1102

0 301aa, >Adeh_0286||RR|PleD|R|Orphan|... *

>Cluster 1103

0 298aa, >PPSIR1_38796||HK|Classic|T||... *

>Cluster 1104

0 295aa, >Hoch_0280||RR|PleD|R|Orphan|... *

>Cluster 1105

0 290aa, >PPSIR1_34357||HK|Classic|T||... *

>Cluster 1106

0 289aa, >PPSIR1_13855||RR|OmpR|R||... *

>Cluster 1107

0 288aa, >Hoch_5217||RR|unclassified|R|Orphan|... *

>Cluster 1108

0 287aa, >PPSIR1_33114||RR|CheY|R||... *

>Cluster 1109

0 286aa, >sce0012||RR|unclassified|R|Orphan|... *

>Cluster 1110

0 286aa, >sce6552||RR|unclassified|R|Orphan|... *

>Cluster 1111

0 281aa, >Hoch_3737||RR|unclassified|R|Orphan|... *

>Cluster 1112

0 280aa, >Hoch_5999||RR|unclassified|R|Orphan|... *

>Cluster 1113

0 278aa, >STAUR_0837||RR|CheY|R|Complex|... *

>Cluster 1114

0 274aa, >Hoch_6434||RR|unclassified|R|Orphan|... *

>Cluster 1115

0 273aa, >STAUR_8302||RR|LytTR|R|Pair|... *

>Cluster 1116

0 273aa, >sce2956||HK|Classic|T|Complex|... *

>Cluster 1117

0 270aa, >A176_6989||RR|LytTR|R||... *

>Cluster 1118

0 268aa, >PPSIR1_37489||HK|Classic|T||... *

>Cluster 1119

0 267aa, >PPSIR1_24884||RR|LytTR|R||... *

>Cluster 1120

0 266aa, >COCOR_01810||RR|LytTR|R|Pair|... *

>Cluster 1121

0 265aa, >Hoch_6446||RR|unclassified|R|Orphan|... *

>Cluster 1122

0 265aa, >sce5180||HK|Classic|T|Orphan|... *

>Cluster 1123

0 265aa, >sce6353||RR|OmpR|R|Pair|... *

>Cluster 1124

0 265aa, >sce7574||RR|unclassified|R|Orphan|... *

>Cluster 1125

0 264aa, >Hoch_6781||RR|unclassified|R|Complex|... *

>Cluster 1126

0 262aa, >STAUR_7856||RR|unclassified|R|Orphan|... *

>Cluster 1127

0 261aa, >COCOR_02285||RR|LytTR|R|Pair|... *

>Cluster 1128

0 261aa, >sce3611||HK|Classic|T|Orphan|... *

>Cluster 1129

0 260aa, >A176_6829||RR|LytTR|R||... *

>Cluster 1130

0 260aa, >sce5907||RR|unclassified|R|Orphan|... *

>Cluster 1131

0 259aa, >Adeh_1336||RR|unclassified|R|Orphan|... *

>Cluster 1132

0 258aa, >COCOR_04577||RR|CheY|R|Orphan|... *

>Cluster 1133

0 258aa, >Hoch_5622||HK|Classic|T|Orphan|... *

>Cluster 1134

0 258aa, >Hoch_6783||RR|unclassified|HR|Complex|... *

>Cluster 1135

0 257aa, >PPSIR1_08981||RR|CheY|R||... *

>Cluster 1136

0 257aa, >sce4826||RR|OmpR|R|Pair|... *

>Cluster 1137

0 256aa, >sce3288||RR|OmpR|R|Pair|... *

>Cluster 1138

0 254aa, >sce5995||RR|unclassified|R|Orphan|... *

>Cluster 1139

0 252aa, >sce5814||RR|FrzZ|RR|Complex|... *

>Cluster 1140

0 247aa, >A176_4968||RR|LytTR|R||... *

>Cluster 1141

0 244aa, >PPSIR1_36212||RR|CheY|R||... *

>Cluster 1142

0 243aa, >PPSIR1_26363||RR|OmpR|R||... *

>Cluster 1143

0 243aa, >PPSIR1_28308||RR|OmpR|R||... *

>Cluster 1144

0 242aa, >PPSIR1_12358||RR|unclassified|R||... *

>Cluster 1145

0 242aa, >sce5431||RR|NarL|R|Pair|... *

>Cluster 1146

0 241aa, >sce2434||RR|OmpR|R|Pair|... *

>Cluster 1147

0 240aa, >LILAB_13195||RR|unclassified|R|Orphan|... *

>Cluster 1148

0 240aa, >STAUR_5903||RR|OmpR|R|Pair|... *

>Cluster 1149

0 240aa, >PPSIR1_07350||RR|NarL|R||... *

>Cluster 1150

0 240aa, >Hoch_4801||RR|LytTR|R|Pair|... *

>Cluster 1151

0 239aa, >PPSIR1_16495||RR|OmpR|R||... *

>Cluster 1152

0 239aa, >PPSIR1_20324||RR|LytTR|R||... *

>Cluster 1153

0 239aa, >PPSIR1_38786||RR|FrzZ|RR||... *

>Cluster 1154

0 239aa, >Hoch_4589||RR|unclassified|R|Orphan|... *

>Cluster 1155

0 238aa, >PPSIR1_12363||HK|Classic|H||... *

>Cluster 1156

0 238aa, >PPSIR1_39850||RR|LytTR|R||... *

>Cluster 1157

0 237aa, >Hoch_6727||RR|unclassified|R|Pair|... *

>Cluster 1158

0 236aa, >PPSIR1_19644||RR|NarL|R||... *

>Cluster 1159

0 235aa, >sce5073||RR|OmpR|R|Orphan|... *

>Cluster 1160

0 234aa, >Hoch_1874||RR|OmpR|R|Pair|... *

>Cluster 1161

0 233aa, >sce2555||RR|OmpR|R|Pair|... *

>Cluster 1162

0 233aa, >sce8119||RR|NarL|R|Complex|... *

>Cluster 1163

0 232aa, >Anae109_3355||RR|NarL|R|Pair|... *

>Cluster 1164

0 231aa, >STAUR_7969||HK|Classic|T|Complex|... *

>Cluster 1165

0 230aa, >PPSIR1_24924||RR|OmpR|R||... *

>Cluster 1166

0 230aa, >sce8722||HK|Classic|T|Complex|... *

>Cluster 1167

0 229aa, >sce6516||HK|Classic|T|Orphan|... *

>Cluster 1168

0 228aa, >STAUR_3096||RR|OmpR|R|Pair|... *

>Cluster 1169

0 226aa, >PPSIR1_15845||RR|NarL|R||... *

>Cluster 1170

0 226aa, >Hoch_0552||RR|OmpR|R|Pair|... *

>Cluster 1171

0 224aa, >MXAN_5212||RR|OmpR|R|Pair|... *

>Cluster 1172

0 224aa, >sce7035||RR|unclassified|R|Orphan|... *

>Cluster 1173

0 223aa, >STAUR_4238||RR|CheY|R|Orphan|... *

>Cluster 1174

0 221aa, >Hoch_1974||RR|PrrA|R|Orphan|... *

>Cluster 1175

0 220aa, >PPSIR1_26131||RR|NarL|R||... *

>Cluster 1176

0 218aa, >LILAB_21510||HK|Classic|T|Orphan|... *

>Cluster 1177

0 218aa, >MXAN_6149||RR|NarL|R|Pair|... *

>Cluster 1178

0 218aa, >A176_0860||RR|OmpR|R||... *

>Cluster 1179

0 217aa, >PPSIR1_12598||RR|unclassified|R||... *

>Cluster 1180

0 214aa, >sce3169||RR|unclassified|R|Orphan|... *

>Cluster 1181

0 212aa, >Anae109_1385||RR|NarL|R|Pair|... *

>Cluster 1182

0 212aa, >Hoch_1846||RR|NarL|R|Pair|... *

>Cluster 1183

0 212aa, >sce3965||RR|NarL|R|Orphan|... *

>Cluster 1184

0 210aa, >Hoch_2214||PP|HisKa|H|Orphan|... *

>Cluster 1185

0 209aa, >sce3742||HK|Classic|T|Orphan|... *

>Cluster 1186

0 207aa, >STAUR_0563||RR|unclassified|R|Orphan|... *

>Cluster 1187

0 206aa, >sce0031||RR|unclassified|R|Orphan|... *

>Cluster 1188

0 204aa, >COCOR_01854||RR|NarL|R|Pair|... *

>Cluster 1189

0 203aa, >sce8796||RR|unclassified|R|Complex|... *

>Cluster 1190

0 200aa, >STAUR_7505||RR|NarL|R|Pair|... *

>Cluster 1191

0 198aa, >Anae109_1603||RR|unclassified|R|Orphan|... *

>Cluster 1192

0 195aa, >Hoch_5845||RR|PrrA|R|Pair|... *

>Cluster 1193

0 195aa, >sce3788||RR|unclassified|R|Orphan|... *

>Cluster 1194

0 193aa, >MXAN_3605||RR|unclassified|R|Pair|... *

>Cluster 1195

0 193aa, >sce1417||RR|PrrA|R|Pair|... *

>Cluster 1196

0 191aa, >sce1178||RR|unclassified|R|Orphan|... *

>Cluster 1197

0 185aa, >sce7597||RR|PrrA|R|Pair|... *

>Cluster 1198

0 178aa, >Hoch_3339||RR|unclassified|R|Pair|... *

>Cluster 1199

0 175aa, >sce1593||RR|CheY|R|Pair|... *

>Cluster 1200

0 174aa, >Anae109_2391||RR|CheY|R|Orphan|... *

>Cluster 1201

0 172aa, >PPSIR1_19749||RR|CheY|R||... *

>Cluster 1202

0 171aa, >PPSIR1_16230||RR|CheY|R||... *

>Cluster 1203

0 170aa, >STAUR_4538||RR|CheY|R|Complex|... *

>Cluster 1204

0 169aa, >Anae109_1593||RR|unclassified|R|Orphan|... *

>Cluster 1205

0 167aa, >sce6164||RR|CheY|R|Orphan|... *

>Cluster 1206

0 165aa, >STAUR_5806||RR|CheY|R|Pair|... *

>Cluster 1207

0 165aa, >sce2677||RR|CheY|R|Complex|... *

>Cluster 1208

0 162aa, >STAUR_8017||RR|CheY|R|Complex|... *

>Cluster 1209

0 161aa, >COCOR_07878||RR|CheY|R|Orphan|... *

>Cluster 1210

0 161aa, >sce2424||RR|CheY|R|Orphan|... *

>Cluster 1211

0 159aa, >COCOR_07541||RR|CheY|R|Complex|... *

>Cluster 1212

0 158aa, >Hoch_1802||RR|CheY|R|Orphan|... *

>Cluster 1213

0 157aa, >sce6408||RR|CheY|R|Orphan|... *

>Cluster 1214

0 156aa, >sce6967||RR|CheY|R|Complex|... *

>Cluster 1215

0 156aa, >sce8072||RR|CheY|R|Orphan|... *

>Cluster 1216

0 155aa, >LILAB_07365||RR|CheY|R|Complex|... *

>Cluster 1217

0 155aa, >STAUR_3844||RR|CheY|R|Orphan|... *

>Cluster 1218

0 155aa, >sce7099||RR|CheY|R|Orphan|... *

>Cluster 1219

0 154aa, >sce2955||RR|CheY|R|Complex|... *

>Cluster 1220

0 153aa, >sce1345||RR|CheY|R|Complex|... *

>Cluster 1221

0 153aa, >sce4841||RR|CheY|R|Orphan|... *

>Cluster 1222

0 152aa, >STAUR_1112||RR|CheY|R|Orphan|... *

>Cluster 1223

0 152aa, >PPSIR1_22591||RR|CheY|R||... *

>Cluster 1224

0 151aa, >STAUR_0447||RR|CheY|R|Complex|... *

>Cluster 1225

0 151aa, >STAUR_4270||RR|CheY|R|Orphan|... *

>Cluster 1226

0 151aa, >Anae109_4252||RR|CheY|R|Complex|... *

>Cluster 1227

0 150aa, >STAUR_8014||RR|CheY|R|Complex|... *

>Cluster 1228

0 150aa, >Hoch_5144||RR|CheY|R|Complex|... *

>Cluster 1229

0 148aa, >Hoch_1357||RR|CheY|R|Pair|... *

>Cluster 1230

0 148aa, >sce3849||RR|CheY|R|Orphan|... *

>Cluster 1231

0 147aa, >STAUR_5659||PP|HPt|H|Pair|... *

>Cluster 1232

0 146aa, >COCOR_07528||RR|CheY|R|Complex|... *

>Cluster 1233

0 146aa, >sce6013||RR|CheY|R|Complex|... *

>Cluster 1234

0 146aa, >sce7808||RR|CheY|R|Complex|... *

>Cluster 1235

0 145aa, >Hoch_4440||RR|CheY|R|Pair|... *

>Cluster 1236

0 144aa, >Hoch_3002||RR|CheY|R|Pair|... *

>Cluster 1237

0 143aa, >STAUR_7973||RR|CheY|R|Pair|... *

>Cluster 1238

0 143aa, >Anae109_1305||RR|CheY|R|Orphan|... *

>Cluster 1239

0 142aa, >Anae109_3365||RR|CheY|R|Orphan|... *

>Cluster 1240

0 142aa, >PPSIR1_35007||RR|CheY|R||... *

>Cluster 1241

0 141aa, >PPSIR1_28776||RR|CheY|R||... *

>Cluster 1242

0 140aa, >Anae109_3069||RR|CheY|R|Pair|... *

>Cluster 1243

0 139aa, >PPSIR1_08961||RR|CheY|R||... *

>Cluster 1244

0 138aa, >STAUR_4296||RR|CheY|R|Orphan|... *

>Cluster 1245

0 138aa, >Hoch_4725||RR|CheY|R|Complex|... *

>Cluster 1246

0 138aa, >Hoch_5796||RR|CheY|R|Orphan|... *

>Cluster 1247

0 137aa, >Adeh_0564||RR|CheY|R|Complex|... *

>Cluster 1248

0 137aa, >sce8120||RR|CheY|R|Complex|... *

>Cluster 1249

0 136aa, >COCOR_03616||RR|CheY|R|Complex|... *

>Cluster 1250

0 136aa, >STAUR_6800||RR|CheY|R|Orphan|... *

>Cluster 1251

0 136aa, >PPSIR1_27443||RR|CheY|R||... *

>Cluster 1252

0 136aa, >PPSIR1_42114||RR|CheY|R||... *

>Cluster 1253

0 136aa, >sce1397||RR|unclassified|R|Pair|... *

>Cluster 1254

0 136aa, >sce6152||PP|HisKa|H|Orphan|... *

>Cluster 1255

0 136aa, >sce8458||RR|CheY|R|Orphan|... *

>Cluster 1256

0 135aa, >COCOR_08066||RR|CheY|R|Orphan|... *

>Cluster 1257

0 134aa, >PPSIR1_09311||RR|CheY|R||... *

>Cluster 1258

0 134aa, >Hoch_4809||RR|CheY|R|Orphan|... *

>Cluster 1259

0 134aa, >sce1727||RR|CheY|R|Complex|... *

>Cluster 1260

0 132aa, >COCOR_02339||RR|CheY|R|Complex|... *

>Cluster 1261

0 132aa, >COCOR_06453||RR|CheY|R|Orphan|... *

>Cluster 1262

0 130aa, >LILAB_07890||RR|unclassified|R|Orphan|... *

>Cluster 1263

0 130aa, >COCOR_08023||RR|CheY|R|Orphan|... *

>Cluster 1264

0 130aa, >STAUR_7220||RR|CheY|R|Complex|... *

>Cluster 1265

0 130aa, >Hoch_5448||RR|CheY|R|Pair|... *

>Cluster 1266

0 130aa, >Hoch_6071||RR|CheY|R|Orphan|... *

>Cluster 1267

0 129aa, >STAUR_5250||RR|CheY|R|Orphan|... *

>Cluster 1268

0 129aa, >Hoch_6782||RR|CheY|R|Complex|... *

>Cluster 1269

0 129aa, >Hoch_6875||RR|CheY|R|Orphan|... *

>Cluster 1270

0 128aa, >PPSIR1_17510||RR|CheY|R||... *

>Cluster 1271

0 128aa, >Hoch_1916||RR|CheY|R|Orphan|... *

>Cluster 1272

0 128aa, >Hoch_2281||RR|CheY|R|Complex|... *

>Cluster 1273

0 127aa, >STAUR_8253||RR|CheY|R|Orphan|... *

>Cluster 1274

0 127aa, >Anae109_1496||RR|CheY|R|Pair|... *

>Cluster 1275

0 127aa, >sce1174||RR|CheY|R|Complex|... *

>Cluster 1276

0 127aa, >sce3027||RR|CheY|R|Orphan|... *

>Cluster 1277

0 126aa, >COCOR_05191||RR|CheY|R|Orphan|... *

>Cluster 1278

0 125aa, >sce0803||RR|CheY|R|Pair|... *

>Cluster 1279

0 125aa, >sce1190||RR|CheY|R|Pair|... *

>Cluster 1280

0 125aa, >sce1650||RR|CheY|R|Complex|... *

>Cluster 1281

0 124aa, >Anae109_2029||RR|CheY|R|Pair|... *

>Cluster 1282

0 124aa, >Anae109_3277||RR|CheY|R|Orphan|... *

>Cluster 1283

0 124aa, >sce2051||RR|CheY|R|Complex|... *

>Cluster 1284

0 124aa, >sce7762||RR|CheY|R|Pair|... *

>Cluster 1285

0 124aa, >sce8606||RR|CheY|R|Orphan|... *

>Cluster 1286

0 123aa, >STAUR_0620||RR|CheY|R|Orphan|... *

>Cluster 1287

0 123aa, >PPSIR1_21809||RR|CheY|R||... *

>Cluster 1288

0 123aa, >sce8723||RR|CheY|R|Complex|... *

>Cluster 1289

0 121aa, >COCOR_00236||RR|CheY|R|Pair|... *

>Cluster 1290

0 121aa, >PPSIR1_10760||RR|CheY|R||... *

>Cluster 1291

0 120aa, >COCOR_07481||RR|CheY|R|Orphan|... *

>Cluster 1292

0 120aa, >Anae109_2954||RR|unclassified|R|Orphan|... *

>Cluster 1293

0 119aa, >sce6153||HK|Classic|T|Orphan|... *

>Cluster 1294

0 118aa, >Hoch_4816||RR|CheY|R|Orphan|... *

>Cluster 1295

0 118aa, >sce8800||RR|CheY|R|Complex|... *

>Cluster 1296

0 117aa, >sce2505||RR|CheY|R|Pair|... *

>Cluster 1297

0 117aa, >sce8556||RR|CheY|R|Orphan|... *

>Cluster 1298

0 116aa, >STAUR_6735||RR|CheY|R|Orphan|... *

>Cluster 1299

0 116aa, >Anae109_3105A||RR|unclassified|R|Complex|... *

>Cluster 1300

0 115aa, >Adeh_0512||RR|CheY|R|Orphan|... *

>Cluster 1301

0 111aa, >MXAN_0799||PP|HisKa|H|Orphan|... *

>Cluster 1302

0 111aa, >Hoch_0490||RR|CheY|R|Orphan|... *

>Cluster 1303

0 111aa, >Hoch_5142||PP|HPt|H|Complex|... *

>Cluster 1304

0 105aa, >Hoch_6780||RR|CheY|R|Complex|... *

>Cluster 1305

0 103aa, >sce0780||RR|CheY|R|Orphan|... *

>Cluster 1306

0 102aa, >Hoch_3757||RR|CheY|R|Orphan|... *

>Cluster 1307

0 67aa, >PPSIR1_38791||RR|CheY|R||... *

CD-HIT output for myxobacterial TCS genes, with clustering cut-off 50%.

Clusters exhibiting changes in gene organisation between *Mx*, *Mf*, *Cc* and *Sa* or between *Ad1*, *AdC*, *AK* and *AF* are indicated with a ‘$’ or an ‘£’ respectively.

Clusters exhibiting changes in protein size between *Mx*, *Mf*, *Cc* and *Sa* are indicated with a ‘&’.

Clusters containing duplications of Mx, Mf, Cc, Sa, Ad1, AdC, AK or AF genes are indicated with a ^
