# Supplementary material for: Genome-wide analysis of myxobacterial two-component systems: genome relatedness and evolutionary changes
Source: BMC Genomics. 2015 Oct 13;16:780. doi: 10.1186/s12864-015-2018-y (PMC4603909; doi:10.1186/s12864-015-2018-y)
Supplement: Additional file 4: Table S1. — Intricate multi-gene TCS foci. Each row shows all myxobacterial orthologues of intricate multi-gene foci, including non-intricate orthologues, arranged by organism. Foci in particular genomes are denoted by the number from the locus tag of the first protein within the focus. ‘//’ refers to an unspecified number (2 or more) of non-TCS genes. (PDF 39 kb) [file 12864_2015_2018_MOESM4_ESM.pdf]

Organisation	*Mx*	*Mf*	*Cc*	*Sa*	*Ad1*	*AdC*	*AK*	*AF*	*Sc*	*Ho*
TRR,RTR	0230	07550	07813	0289						
T,RR,T,R	*redC*/0459	06400		7971						
RTRR,T	4445	30360	03338	4801						
RT,R,RT<br>R,RT			00131	 0461						
T,RTR<br>RT			04616	 5100						
TRT,TRT<br>TRTTRT			01454	8144<br>6958						
RTR,TRRR<br>TRRR,RT,TRT<br>TRRR,RT<br>TRT	 <br>6735	 <br>12790	 <br>07311<br>05180	 1169						2075
TR,R,T	6953	11650	7521	0871						
T,R,TRR,RHRR<br>R,TRR,RHRR	 7364		07950							
T,R,RT		03710	00946	7006						
T,R,RT		04825		7451						
RRT,R,T<br>RT,//,R,T	6966	11575	 07558/07539	 0856/0859						
T,RR,RT	4246	29365			1329	2626	2530	2438		
TR,R,T<br>TR,R					2773	1193	2678	 2297		
R,RT,T,R,RT				08017						
R,TRRH									1345	
TRT,R									2653	
RT,TR									4956	
TR,RTR									6827	
TRRH,R									7776	
T,R,TRH,RT									7809	
TR,R,RT									8715	

R,T,T,RR,R<br>T,T,RR									8796<br>5168	
TR,R,RRRT										2367
RTRR,T										1854
TR,RT										0803
TRT,TR										0539

Additional Table 1: Intricate multi-gene TCS foci. Each row shows all myxobacterial orthologues of intricate multi-gene foci, including non-intricate orthologues, arranged by organism. Foci in particular genomes are denoted by the number from the locus tag of the first protein within the focus. ‘//’ refers to an unspecified number (2 or more) of non-TCS genes.
