# Supplementary material for: Genome-wide analysis of myxobacterial two-component systems: genome relatedness and evolutionary changes
Source: BMC Genomics. 2015 Oct 13;16:780. doi: 10.1186/s12864-015-2018-y (PMC4603909; doi:10.1186/s12864-015-2018-y)
Supplement: Additional file 5: Table S2. — Evidence of horizontal gene transfer (HGT) amongst cystobacterial TCS proteins. Singletons (TCS lacking orthologues) from comparisons between Ad1/AdC/AK/AF, and between Mx/Mf/Cc/Sa, are presented alongside the number of singletons acquired by HGT (as evidenced by highest-scoring BLAST hits to non-myxobacteria outside the organism’s own genus). Also tabulated are the number of TCS genes found in foci where every TCS gene was acquired by putative HGT and the number of those which constitute an ‘intact’ TCS (ie containing ≥1 transmitter domain and ≥1 receiver domain. (PDF 456 kb) [file 12864_2015_2018_MOESM5_ESM.pdf]

Organism	TCS Genes	Singletons	Singletons acquired by HGT	Genes found within entire foci acquired by HGT	HGT foci containing ‘intact’ TCS.
Ad1	188	2	1	1	0
AdC	187	8	6	6	1
AK	191	3	3	3	2
AF	207	65	24	20	15
Sa	338	124	22	18	13
Cc	306	56	12	9	5
Mx	282	13	7	7	6
Mf	288	10	2	1	0
TOTAL	1987	281	77	65	42

Additional Table 2. Evidence of horizontal gene transfer (HGT) amongst cystobacterial TCS proteins. Singletons (TCS lacking orthologues) from comparisons between *Ad1/AdC/AK/AF*, and between *Mx/Mf/Cc/Sa*, are presented alongside the number of singletons acquired by HGT (as evidenced by highest-scoring BLAST hits to non-myxobacteria outside the organism’s own genus). Also tabulated are the number of TCS genes found in foci where every TCS gene was acquired by putative HGT and the number of those which constitute an ‘intact’ TCS (ie containing ≥1 transmitter domain and ≥1 receiver domain.
